# Statistical modelling of citation exchange between statistics journals

**DOI:** 10.1111/rssa.12124

**Published:** 2015-11-03

**Authors:** Cristiano Varin, Manuela Cattelan, David Firth

**Affiliations:** ^1^ Università Ca' FoscariVeneziaItaly; ^2^ Università degli Studi di PadovaItaly; ^3^ University of WarwickCoventryUK

**Keywords:** Bradley–Terry model, Citation data, Export score, Impact factor, Journal ranking, Research evaluation, Stigler model

## Abstract

Rankings of scholarly journals based on citation data are often met with scepticism by the scientific community. Part of the scepticism is due to disparity between the common perception of journals’ prestige and their ranking based on citation counts. A more serious concern is the inappropriate use of journal rankings to evaluate the scientific influence of researchers. The paper focuses on analysis of the table of cross‐citations among a selection of statistics journals. Data are collected from the *Web of Science* database published by Thomson Reuters. Our results suggest that modelling the exchange of citations between journals is useful to highlight the most prestigious journals, but also that journal citation data are characterized by considerable heterogeneity, which needs to be properly summarized. Inferential conclusions require care to avoid potential overinterpretation of insignificant differences between journal ratings. Comparison with published ratings of institutions from the UK's research assessment exercise shows strong correlation at aggregate level between assessed research quality and journal citation ‘export scores’ within the discipline of statistics.

## 1 Introduction

The problem of ranking scholarly journals has arisen partly as an economic matter. When the number of scientific journals started to increase, librarians were faced with decisions about which journal subscriptions should consume their limited economic resources; a natural response was to be guided by the relative importance of different journals according to a published or otherwise agreed ranking. Gross and Gross (1927) proposed the counting of citations received by journals as a direct measure of their importance. Garfield (1955) suggested that the number of citations received should be normalized by the number of citable items published by a journal. This idea is at the origin of the *impact factor*, which is the best‐known index for ranking journals. Published since the 1960s, the impact factor is ‘an average citation rate per published article’ (Garfield, 1972).

The impact factor of the journals where scholars publish has also been employed—improperly, many might argue—in appointing to academic positions, in awarding research grants and in ranking universities and their departments. The ‘San Francisco declaration on research assessment’ (http://am.ascb.org/dora, 2013) and the Institute of Electrical and Electronics Engineers position statement on ‘Appropriate use of bibliometric indicators for the assessment of journals, research proposals, and individuals’ (Institute of Electrical and Electronics Engineers Board of Directors, 2013) are just two of the most recent authoritative standpoints regarding the risks of automatic, metric‐based evaluations of scholars. Typically, only a small fraction of all published articles accounts for most of the citations that are received by a journal (Seglen, 1997). Single authors should ideally be evaluated on the basis of their own outputs and not through citations of other papers that have appeared in the journals where their papers have been published (Seglen, 1997; Adler *et al*., 2009; Silverman, 2009). As stated in a recent *Science* editorial about impact factor distortions (Alberts, 2013),

‘… the leaders of the scientific enterprise must accept full responsibility for thoughtfully analyzing the scientific contributions of other researchers. To do so in a meaningful way requires the actual reading of a small selected set of each researcher's publications, a task that must not be passed by default to journal editors’.

Indicators derived from citations received by papers written by a particular author (e.g. Bornmann and Marx (2014)) can be a useful complement for evaluation of trends and patterns of that author's impact, but not a substitute for the reading of papers.

Journal rankings based on the impact factor often differ substantially from common perceptions of journal prestige (Theoharakis and Skordia, 2003; Arnold and Fowler, 2011). Various causes of such discrepancy have been pointed out. First, there is the phenomenon that more ‘applied’ journals tend to receive citations from other scientific fields more often than do journals that publish theoretical work. This may be related to uncounted ‘indirect citations’ arising when methodology that is developed in a theoretical journal is then popularized by papers published in applied journals accessible to a wider audience and thus receiving more citations than the original source (Journal‐Ranking.com, 2007; Putirka *et al*., 2013). Second is the short time period that is used for computation of the impact factor, which can be completely inappropriate for some fields, in particular for mathematics and statistics (van Nierop, 2009; Arnold and Fowler, 2011). Finally, there is the risk of manipulation, whereby authors might be asked by journal editors to add irrelevant citations to other papers published in their journal (Sevinc, 2004; Frandsen, 2007; Archambault and Larivière, 2009; Arnold and Fowler, 2011). According to a large survey published in *Science* (Wilhite and Fong, 2012), about 20% of academics in social science and business fields have been asked to ‘pad their papers with superfluous references to get published’ (van Noorden, 2012). The survey data also suggest that junior faculty members are more likely to be pressured to cite superfluous papers. Recently, Thomson Reuters has started to publish the impact factor both with and without journal self‐citations, thereby allowing evaluation of the contribution of self‐citations to the impact factor calculation. Moreover, Thomson Reuters has occasionally excluded journals with an excessive self‐citation rate from the ‘Journal citation reports’ (JCRs).

Given these criticisms, it is not surprising that the impact factor and other ‘quantitative’ journal rankings have given rise to substantial scepticism about the value of citation data. Several proposals have been developed in the bibliometric literature to overcome the weaknesses of the impact factor; examples include the *article influence score* (Bergstrom, 2007; West, 2010), the *H‐index* for journals (Braun *et al*., 2006; Pratelli *et al*., 2012), the *source‐normalized impact per paper* index (Waltman *et al*., 2013) and methods based on percentile rank classes (Marx and Bornmann, 2013).

The aforementioned *Science* editorial (Alberts, 2013) reports that

‘… in some nations, publication in a journal with an impact factor below 5.0 is officially of zero value’.

In the latest edition (2013) of the JCR, the only journal with an impact factor larger than 5 in the category ‘Statistics and probability’ was the *Journal of the Royal Statistical Society*, Series B, with impact factor 5.721. The category ‘Mathematics’ achieved still lower impact factors, with the highest value there in 2013 being 3.08 for *Communications on Pure and Applied Mathematics*. Several bibliometric indicators have been developed, or adjusted, to allow for cross‐field comparisons, e.g. Leydesdorff *et al*. (2013) and Waltman and Van Eck (2013), and could be considered to alleviate unfair comparisons. However, our opinion is that comparisons between different research fields will rarely make sense, and that such comparisons should be avoided. Research fields differ very widely, e.g. in terms of the frequency of publication, the typical number of authors per paper and the typical number of citations made in a paper, as well as in the sizes of their research communities. Journal homogeneity is a minimal prerequisite for a meaningful statistical analysis of citation data (Lehmann *et al*., 2009).

Journal citation data are unavoidably characterized by substantial variability (e.g. Amin and Mabe (2000)). A clear illustration of this variability, suggested by the Associate Editor for this paper, comes from an early editorial of *Briefings in Bioinformatics* (Bishop and Bird, 2007) announcing that this new journal had received an impact factor of 24.37. However, the editors noted that a very large fraction of the journal's citations came from a single paper; if that paper were to be dropped, then the journal's impact factor would decrease to about 4. The variability of the impact factor is inherently related to the heavy‐tailed distribution of citation counts. Averaged indicators such as the impact factor are clearly unsuitable for summarizing highly skew distributions. Nevertheless, quantification of uncertainty is typically lacking in published rankings of journals. A recent exception is Chen *et al*. (2014) who employed a bootstrap estimator for the variability of journal impact factors. Also the source‐normalized impact per paper indicator that was published by Leiden University's Centre for Science and Technology Studies based on theElsevier Scopus database, and available on line at www.journalindicators.com, is accompanied by a ‘stability interval’ computed via a bootstrap method. See also Hall and Miller (2009, 2010) and references therein for more details on statistical assessment of the authority of rankings.

The impact factor was developed to identify which journals have the greatest influence on subsequent research. The other metrics that are mentioned in this paper originated as possible improvements on the impact factor, with the same aim. Palacios‐Huerta and Volij (2004) listed a set of properties that a ranking method which measures the intellectual influence of journals, by using citation counts, should satisfy. However, the list of all desirable features of a ranking method might reasonably be extended to include features other than citations, depending on the purpose of the ranking. For example, when librarians decide which journals to take, they should consider the influence of a journal in one or more research fields, but they may also take into account its cost effectiveness. The Web site www.journalprices.com, which is maintained by Professor Ted Bergstrom and Professor Preston McAfee, ranks journals according to their price per article, price per citation and a composite index.

A researcher when deciding where to submit a paper most probably considers each candidate journal's record of publishing papers on similar topics, and the importance of the journal in the research field; but he or she may also consider the speed of the reviewing process, the typical time between acceptance and publication of the paper, possible page charges, and the likely effect on his or her own career. Certain institutions and national evaluation agencies publish rankings of journals which are used to evaluate researcher performance and to inform the hiring of new faculty members. For various economics and management‐related disciplines, the ‘*Journal quality list*’, which is compiled by Professor Anne‐Wil Harzing and is available at www.harzing.com/jql.htm, combines more than 20 different rankings made by universities or evaluation agencies in various countries. Such rankings typically are based on bibliometric indices, expert surveys or a mix of both.

Modern technologies have fostered the rise of alternative metrics such as ‘webometrics’ based on citations on the Internet or numbers of downloads of articles. Recently, interest has moved from Web citation analysis to social media usage analysis. In some disciplines the focus is now towards broader measurement of research impact through the use of Web‐based quantities such as citations in social media sites, newspapers, government policy documents and blogs. This is mainly implemented at the level of individual articles (see for example the Altmetric service (Adie and Roe, 2013) which is available at www.altmetric.com), but the analysis may also be made at journal level. Along with the advantages of timeliness, availability of data and consideration of different sources, such measures also have certain drawbacks related to data quality, possible bias and data manipulation (Bornmann, 2014).

A primary purpose of the present paper is to illustrate the risks of overinterpretation of insignificant differences between journal ratings. In particular, we focus on the analysis of the exchange of citations between a relatively homogeneous list of journals. Following Stigler (1994), we model the table of cross‐citations between journals in the same field by using a Bradley–Terry model (Bradley and Terry, 1952) and thereby derive a ranking of the journals’ ability to ‘export intellectual influence’ (Stigler, 1994). Although the Stigler approach has desirable properties and is sufficiently simple to be promoted also outside the statistics community, there have been rather few published examples of application of this model since its first appearance; Stigler *et al*. (1995) and Liner and Amin (2004) are two notable examples of its application to the journals of economics.

We pay particular attention to methods that summarize the uncertainty in a ranking produced through the Stigler model‐based approach. Our focus on properly accounting for ‘model‐based uncertainty in making comparisons’ is close in spirit to Goldstein and Spiegelhalter (1996). We propose to fit the Stigler model with the quasi‐likelihood method (Wedderburn, 1974) to account for interdependence between the citations exchanged between pairs of journals, and to summarize estimation uncertainty by using quasi‐variances (Firth and de Menezes, 2005). We also suggest the use of the ranking lasso penalty (Masarotto and Varin, 2012) when fitting the Stigler model, to combine the benefits of shrinkage with an enhanced interpretation arising from automatic presentational grouping of journals with similar merits.

The paper is organized as follows. Section 2 describes the data collected from the *Web of Science* database compiled by Thomson Reuters; then, as preliminary background to the paper's main content on journal rankings, Section 3 illustrates the use of cluster analysis to identify groups of statistics journals sharing similar aims and types of content. Section 4 provides a brief summary of journal rankings published by Thomson Reuters in the JCRs. Section 5 discusses the Stigler method and applies it to the table of cross‐citations between statistics journals. Section 6 compares journal ratings based on citation data with results from the UK research assessment exercise, and Section 7 collects some concluding remarks.

The citation data set and the computer code used for the analyses written in the R language (R Core Team, 2015) are available from

http://wileyonlinelibrary.com/journal/rss-datasets

## 2 The *Web of Science* database

The database that was used for our analyses is the 2010 edition of the *Web of Science* that was produced by Thomson Reuters. The citation data contained in the database are employed to compile the JCRs, whose science edition summarizes citation exchange between more than 8000 journals in science and technology. Within the JCR, scholarly journals are grouped into 171 overlapping subject categories. In particular, in 2010 the ‘Statistics and probability’ category comprised 110 journals. The choice of the journals that are encompassed in this category is to some extent arbitrary. The Scopus database, which is the main commercial competitor of the *Web of Science*, in 2010 included in its statistics and probability category 105 journals, but only about two‐thirds of them were classified in the same category within the *Web of Science*. The statistics and probability category contains also journals related to fields such as econometrics, chemistry, computational biology, engineering and psychometrics.

A severe criticism of the impact factor relates to the time period that is used for its calculation. The standard version of the impact factor considers citations received to articles published in the previous 2 years. This period is too short to reach the peak of citations of an article, especially in mathematical disciplines (Hall, 2009). van Nierop (2009) found that articles published in statistics journals typically reach the peak of their citations more than 3 years after publication; as reported by the JCR, the median age of the articles cited in this category is more than 10 years. Thomson Reuters acknowledges this issue and computes a second version of the impact factor using citations to papers published in the previous 5 years. Recent published alternatives to the impact factor, to be discussed in Section 4, also count citations to articles that appeared in the previous 5 years. The present paper considers citations of articles published in the previous 10 years, to capture the influence, over a more substantial period, of work published in statistical journals.

A key requirement for the methods that are described here, as well as in our view for any sensible analysis of citation data, is that the journals jointly analysed should be as homogeneous as possible. Accordingly, analyses are conducted on a subset of the journals from the statistics and probability category, among which there is a relatively high level of citation exchange. The selection is obtained by discarding journals in probability, econometrics, computational biology, chemometrics and engineering, and other journals that are not sufficiently related to the majority of the journals in the selection. Furthermore, journals recently established, and thus lacking a record of 10 years of citable items, also are dropped. The final selection consists of the 47 journals that are listed in Table 1. Obviously, the methods that are discussed in this paper can be similarly applied to other selections motivated by different purposes. For example, a statistician who is interested in applications to economics might consider a different selection with journals of econometrics and statistical methodology, discarding instead journals oriented towards biomedical applications.

**Table 1:** List of selected statistics journals, with abbreviations used in the paper

*Journal name*	*Abbreviation*
*American Statistician*	AmS
*Annals of the Institute of Statistical Mathematics*	AISM
*Annals of Statistics*	AoS
*Australian and New Zealand Journal of Statistics*	ANZS
*Bernoulli*	Bern
*Biometrical Journal*	BioJ
*Biometrics*	Bcs
*Biometrika*	Bka
*Biostatistics*	Biost
*Canadian Journal of Statistics*	CJS
*Communications in Statistics*—*Simulation and*	CSSC
*Computation*	
*Communications in Statistics*—*Theory and Methods*	CSTM
*Computational Statistics*	CmpSt
*Computational Statistics and Data Analysis*	CSDA
*Environmental and Ecological Statistics*	EES
*Environmetrics*	Envr
*International Statistical Review*	ISR
*Journal of Agricultural, Biological and*	JABES
*Environmental Statistics*	
*Journal of the American Statistical Association*	JASA
*Journal of Applied Statistics*	JAS
*Journal of Biopharmaceutical Statistics*	JBS
*Journal of Computational and Graphical Statistics*	JCGS
*Journal of Multivariate Analysis*	JMA
*Journal of Nonparametric Statistics*	JNS
*Journal of the Royal Statistical Society*, Series A	JRSS‐A
*Journal of the Royal Statistical Society*, Series B	JRSS‐B
*Journal of the Royal Statistical Society*, Series C	JRSS‐C
*Journal of Statistical Computation and Simulation*	JSCS
*Journal of Statistical Planning and Inference*	JSPI
*Journal of Statistical Software*	JSS
*Journal of Time Series Analysis*	JTSA
*Lifetime Data Analysis*	LDA
*Metrika*	Mtka
*Scandinavian Journal of Statistics*	SJS
*Stata Journal*	StataJ
*Statistical Methods in Medical Research*	SMMR
*Statistical Modelling*	StMod
*Statistica Neerlandica*	StNee
*Statistical Papers*	StPap
*Statistical Science*	StSci
*Statistica Sinica*	StSin
*Statistics*	Stats
*Statistics and Computing*	StCmp
*Statistics in Medicine*	StMed
*Statistics and Probability Letters*	SPL
*Technometrics*	Tech
*Test*	Test

The JCR database supplies detailed information about the citations that are exchanged between pairs of journals through the *cited journal table* and the *citing journal table*. The cited journal table for journal *i* contains the number of times that articles published in journal *j* during 2010 cite articles published in journal *i* in previous years. Similarly, the citing journal table for journal *i* contains the number of times that articles published in journal *j* in previous years were cited in journal *i* during 2010. Both of the tables contain some very modest loss of information. In fact, all journals that cite journal *i* are listed in the cited journal table for journal *i* only if the number of citing journals is less than 25. Otherwise, the cited journal table reports only those journals that cite journal *i* at least twice in *all past years*, thus counting also citations to papers that were published earlier than the decade 2001–2010 considered here. Remaining journals that cite journal *i* only once in all past years are collected in the category ‘all others’. Information on journals cited only once is similarly treated in the citing journal table.

Cited and citing journal tables allow construction of the cross‐citation matrix $C=(c_{ij})$, where $c_{ij}$ is the number of citations from articles published in journal *j* in 2010 to papers published in journal *i* in the chosen time window (*i*=1,…,*n*). In our analyses, *n*=47, the number of selected statistics journals, and the time window is the previous 10 years. In the rest of this section we provide summary information about citations made and received by each statistics journal at aggregate level, whereas Sections 3 and 5 discuss statistical analyses derived from citations exchanged by pairs of journals.

**Figure 1:**
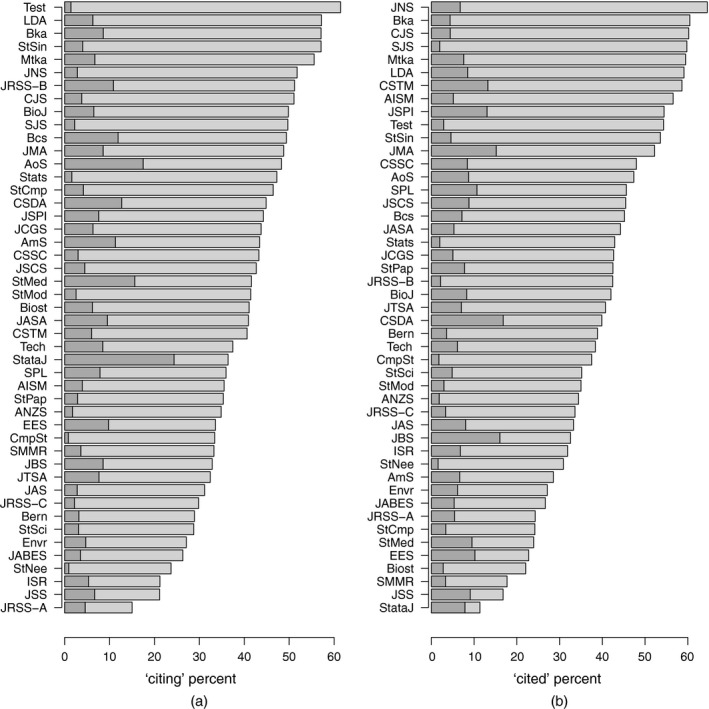
Bar plots of (a) citations made and (b) citations received for the statistics journals selected, as listed in Table 2 based on the 2010 JCR: for each journal, the bar displays the percentage of self‐citations (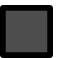) and the percentage of citations made or received to or from other statistics journals in the list (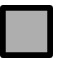)

Table 2 shows the citations made by papers published in each statistics journal in 2010 to papers published in other journals in the decade 2001–2010, as well as the citations that the papers published in each statistics journal in 2001–2010 received from papers published in other journals in 2010. The same information is visualized in the bar plots of Fig. 1. Citations made and received are classified into three categories, namely journal self‐citations from a paper published in a journal to another paper in the same journal, citations to or from journals in the list of selected statistics journals and citations to or from journals not in the selection.

**Table 2:** Citations made, Citing, and received, Cited, in 2010 to or from articles published in 2001–2010†

*Journal*	*Citing*			*Cited*		
	*Total*	*Self*	*Stat*	*Total*	*Self*	*Stat*
AmS	380	0.11	0.43	648	0.07	0.29
AISM	459	0.04	0.36	350	0.05	0.57
AoS	1663	0.17	0.48	3335	0.09	0.47
ANZS	284	0.02	0.35	270	0.02	0.34
Bern	692	0.03	0.29	615	0.04	0.39
BioJ	845	0.07	0.50	664	0.08	0.42
Bcs	1606	0.12	0.49	2669	0.07	0.45
Bka	872	0.09	0.57	1713	0.04	0.60
Biost	874	0.06	0.41	1948	0.03	0.22
CJS	419	0.04	0.51	362	0.04	0.60
CSSC	966	0.03	0.43	344	0.08	0.48
CSTM	1580	0.06	0.41	718	0.13	0.59
CmpSt	371	0.01	0.33	168	0.02	0.38
CSDA	3820	0.13	0.45	2891	0.17	0.40
EES	399	0.10	0.34	382	0.10	0.23
Envr	657	0.05	0.27	505	0.06	0.27
ISR	377	0.05	0.21	295	0.07	0.32
JABES	456	0.04	0.26	300	0.05	0.27
JASA	2434	0.10	0.41	4389	0.05	0.44
JAS	1248	0.03	0.31	436	0.08	0.33
JBS	1132	0.09	0.33	605	0.16	0.33
JCGS	697	0.06	0.44	870	0.05	0.43
JMA	2167	0.09	0.49	1225	0.15	0.52
JNS	562	0.03	0.52	237	0.07	0.65
JRSS‐A	852	0.05	0.15	716	0.05	0.24
JRSS‐B	506	0.11	0.51	2554	0.02	0.42
JRSS‐C	731	0.02	0.30	479	0.03	0.34
JSCS	736	0.04	0.43	374	0.09	0.45
JSPI	3019	0.08	0.44	1756	0.13	0.54
JSS	1361	0.07	0.21	1001	0.09	0.17
JTSA	327	0.08	0.32	356	0.07	0.41
LDA	334	0.06	0.57	247	0.09	0.59
Mtka	297	0.07	0.56	264	0.08	0.59
SJS	493	0.02	0.50	562	0.02	0.60
StataJ	316	0.24	0.36	977	0.08	0.11
SMMR	746	0.04	0.33	813	0.03	0.18
StMod	275	0.03	0.41	237	0.03	0.35
StNee	325	0.01	0.24	191	0.02	0.31
StPap	518	0.03	0.35	193	0.08	0.42
StSci	1454	0.03	0.29	924	0.05	0.35
StSin	1070	0.04	0.57	935	0.05	0.54
Stats	311	0.02	0.47	254	0.02	0.43
StCmp	575	0.04	0.46	710	0.03	0.24
StMed	4022	0.16	0.42	6602	0.10	0.24
SPL	1828	0.08	0.36	1348	0.11	0.46
Tech	494	0.09	0.37	688	0.06	0.38
Test	498	0.01	0.61	243	0.03	0.54

†Columns are total citations, Total, proportion of citations that are journal self‐citations, Self, and proportion of citations that are to or from statistics journals, Stat, including journal self‐citations. Journal abbreviations are as in Table 1.

The total numbers of citations reported in the second and fifth columns of Table 2 include citations given or received by all journals included in the *Web of Science* database, not only those in the field of statistics. The totals are influenced by journals' sizes and by the citation patterns of other categories to which journals are related. The number of references to articles published in 2001–2010 ranges from 275 for citations made in *Statistical Modelling*, which has a small size publishing around 350–400 pages per year, to 4022 for *Statistics in Medicine*, which is a large journal with size ranging from 3500 to 6000 pages annually in the period examined. The number of citations from a journal to articles in the same journal is quite variable and ranges from 0.8% of all citations for *Computational Statistics* to 24% for *Stata Journal*. On average, 6% of the references in a journal are to articles appearing in the same journal and 40% of references are addressed to journals in the list, including journal self‐citations. The *Journal of the Royal Statistical Society*, Series A, has the lowest percentage of citations to other journals in the list, at only 10%. Had we kept the whole ‘Statistics and probability’ category of the JCR, that percentage would have risen, by just 2 points to 12%; most of the references appearing in the *Journal of the Royal Statistical Society*, Series A, are to journals outside the statistics and probability category.

The number of citations received ranges from 168 for *Computational Statistics* to 6602 for *Statistics in Medicine*. Clearly, the numbers are influenced by the size of the journal. For example, the small number of citations received by *Computational Statistics* relates to only around 700 pages published per year by that journal. The citations received are influenced also by the citation patterns of other subject categories. In particular, the number of citations that are received by a journal oriented towards medical applications benefits from communication with a large field including many high impact journals. For example, around 75% of the citations received by *Statistics in Medicine* came from journals outside the list of statistics journals, mostly from medical journals. On average, 7% of the citations received by journals in the list came from the same journal and 40% were from journals in the list.

As stated already, the statistics journals on which we focus have been selected from the statistics and probability category of the JCR, with the aim of retaining those which communicate more. The median fraction of citations from journals discarded from our selection to journals in the list is only 4%, whereas the median fraction of citations received by non‐selected journals from journals in the list is 7%. An important example of an excluded journal is *Econometrica*, which was ranked in leading positions by all the published citation indices. *Econometrica* had only about 2% of its references addressed to other journals in our list, and received only 5% of its citations from journals within our list.

## 3 Clustering journals

Statistics journals have different stated objectives, and different types of content. Some journals emphasize applications and modelling, whereas others focus on theoretical and mathematical developments, or deal with computational and algorithmic aspects of statistical analysis. Applied journals are often targeted to particular areas, such as statistics for medical applications, or for environmental sciences. Therefore, it is quite natural to consider whether the cross‐citation matrix **C** allows the identification of groups of journals with similar aims and types of content. Clustering of scholarly journals has been extensively discussed in the bibliometric literature and a variety of clustering methods have been considered. Examples include the hill climbing method (Carpenter and Narin, 1973), *k*‐means (Boyack *et al*., 2005) and methods based on graph theory (Leydesdorff, 2004; Liu *et al*., 2012).

Consider the total number $t_{ij}$ of citations exchanged between journals *i* and *j*,

(1) $$t_{ij}=\left\{\begin{array}{cc} c_{ij}+c_{ji}, & \;\text{for}\;i\neq j,\\ c_{ii}, & \;\text{for}\;i=j. \end{array}\right.$$

**Figure 2:**
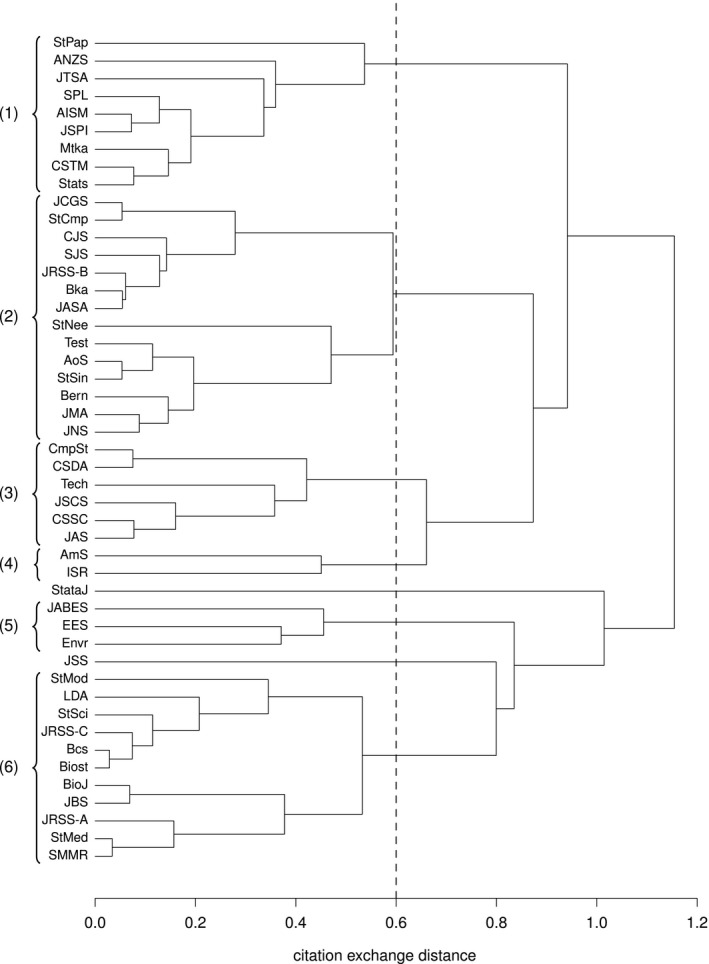
Dendrogram of complete‐linkage hierarchical cluster analysis: clusters obtained by cutting the dendrogram at distance 0.6

Among various possibilities—see, for example, Boyack *et al*. (2005)—the distance between two journals can be measured by quantity $d_{ij}=1-\rho_{ij}$, where $\rho_{ij}$ is the Pearson correlation coefficient of variables $t_{ik}$ and $t_{jk}$ (*k*=1,…,*n*), i.e.

$$\rho_{ij}=\frac{\sum_{k=1}^{n}\left(t_{ik}-{\overset{¯}{t}}_{i}\right)\left(t_{jk}-{\overset{¯}{t}}_{j}\right)}{\surd \left\{\sum_{k=1}^{n}{\left(t_{ik}-{\overset{¯}{t}}_{i}\right)}^{2}\sum_{k=1}^{n}{\left(t_{jk}-{\overset{¯}{t}}_{j}\right)}^{2}\right\}},$$

with ${\overset{¯}{t}}_{i}=\Sigma_{k=1}^{n}t_{ik}/n$. Among the many available clustering algorithms, we consider a hierarchical agglomerative cluster analysis with complete linkage (Kaufman and Rousseeuw, 1990). The clustering process is visualized through the dendrogram in Fig. 2. Visual inspection of the dendrogram suggests cutting it at distance 0.6, thereby obtaining eight clusters, two of which are singletons. The clusters identified are grouped in brackets in Fig. 2.

We comment first on the groups and later on the singletons, following the order of the journals in Fig. 2. The first group, (1), includes a large number of general journals concerned with theory and methods of statistics, but also with applications. Among others, the group includes the *Journal of Time Series Analysis*, the *Journal of Statistical Planning and Inference* and *Annals of the Institute of Statistical Mathematics*.

The second group, (2), contains the leading journals in the development of statistical theory and methods: *Annals of Statistics*,* Biometrika*, the *Journal of the American Statistical Association* and the *Journal of the Royal Statistical Society*, Series B. The group includes also other methodological journals such as *Bernoulli*, the *Scandinavian Journal of Statistics* and *Statistica Sinica*. It is possible to identify some natural subgroups: the *Journal of Computational and Graphical Statistics* and *Statistics and Computing*;* Biometrika*, the *Journal of the Royal Statistical Society*, Series B, and the *Journal of the American Statistical Association*;* Annals of Statistics* and *Statistica Sinica*.

The third group, (3), comprises journals mostly dealing with computational aspects of statistics, such as *Computational Statistics and Data Analysis*,* Communications in Statistics—Simulation and Computation*,* Computational Statistics* and the *Journal of Statistical Computation and Simulation*. Other members of the group with a less direct orientation towards computational methods are *Technometrics* and the *Journal of Applied Statistics*.

The fourth group, (4), includes just two journals both of which publish mainly review articles, namely the *American Statistician* and the *International Statistical Review*.

The fifth group, (5), comprises the three journals specializing in ecological and environmental applications: the *Journal of Agricultural, Biological and Environmental Statistics*,* Environmental and Ecological Statistics* and *Environmetrics*.

The last group, (6), includes various journals emphasizing applications, especially to health sciences and similar areas. It encompasses journals oriented towards biological and medical applications such as *Biometrics* and *Statistics in Medicine*, and also journals publishing papers about more general statistical applications, such as the *Journal of the Royal Statistical Society*, Series A and C. The review journal *Statistical Science* also falls into this group; it is not grouped together with the other two review journals already mentioned. Within the group there are some natural subgroupings: *Statistics in Medicine* with *Statistical Methods in Medical Research*; and *Biometrics* with *Biostatistics*.

Finally, and perhaps not surprisingly, the two singletons are the software‐oriented *Journal of Statistical Software* and *Stata Journal*. The latter is, by some distance, the most remote journal in the list according to the measure of distance that is used here.

## 4 Ranking journals

The Thomson Reuters JCR Web site annually publishes various rating indices, the best‐known being the already mentioned impact factor. Thomson Reuters also publishes the *immediacy index*, which describes the average number of times that an article is cited in the year of its publication. The immediacy index is unsuitable for evaluating statistics journals, but it could be worthy of attention in fields where citations occur very quickly, e.g. some areas of neuroscience and other life sciences.

It is well known in the bibliometric literature that the calculation of the impact factor contains some important inconsistencies (Glänzel and Moed, 2002). The numerator of the impact factor includes citations to all items, whereas the number of citable items in the denominator excludes letters to the editor and editorials; such letters are an important element of some journals, notably medical journals. The inclusion of self‐citations, defined as citations from a journal to articles in the same journal, exposes the impact factor to possible manipulation by editors. Indeed, Sevinc (2004), Frandsen (2007) and Wilhite and Fong (2012) have reported instances where authors were asked to add irrelevant references to their articles, presumably with the aim of increasing the impact factor of the journal. As previously mentioned, recently Thomson Reuters has made available also the impact factor without journal self‐citations. Journal self‐citations can also be a consequence of authors' preferring to cite papers that are published in the same journal instead of equally relevant papers published elsewhere, particularly if they perceive such self‐citation as likely to be welcomed by the journal's editors. Nevertheless, the potential for such behaviour should not lead to the conclusion that self‐citations are always unfair. Many self‐citations are likely to be genuine, especially since scholars often select a journal for submission of their work according to the presence of previously published papers on related topics.

The *eigenfactor score* and the derived *article influence score* (Bergstrom, 2007; West, 2010) have been proposed to overcome the limitations of the impact factor. Both the eigenfactor and the article influence score are computed over a 5‐year time period, with journal self‐citations removed to eliminate possible sources of manipulation. The idea underlying the eigenfactor score is that the importance of a journal relates to the time that is spent by scholars in reading that journal. As stated by Bergstrom (2007), it is possible to imagine that a scholar starts reading an article selected at random. Then, the scholar randomly selects another article from the references of the first paper and reads it. Afterwards, a further article is selected at random from the references that were included in the previous one and the process may go on *ad infinitum*. In such a process, the time that is spent in reading a journal might reasonably be regarded as an indicator of that journal's importance.

Apart from modifications that are needed to account for special cases such as journals that do not cite any other journal, the eigenfactor algorithm is summarized as follows. The eigenfactor is computed from the normalized citation matrix $\tilde{C}=\left({\tilde{c}}_{ij}\right)$, whose elements are the citations $c_{ij}$ from journal *j* to articles published in the previous 5 years in journal *i* divided by the total number of references in *j* in those years, ${\tilde{c}}_{ij}=c_{ij}/\Sigma_{i=1}^{n}c_{ij}$. The diagonal elements of $\tilde{C}$ are set to 0, to discard self‐citations. A further ingredient of the eigenfactor is the vector of normalized numbers of articles $a={\left(a_{1},\ldots ,a_{n}\right)}^{T}$, with $a_{i}$ being the number of articles published by journal *i* during the 5‐year period divided by the number of articles published by all journals considered. Let $e^{T}$ be the row vector of 1s, so that ${\mathrm{ae}}^{T}$ is a matrix with all identical columns **a**. Then

$$P=\lambda \tilde{C}+\left(1-\lambda \right){\mathrm{ae}}^{T}$$

is the transition matrix of a Markov process that assigns probability *λ* to a random movement in the journal citation network, and probability 1−*λ* to a random jump to any journal; for jumps of the latter kind, destination journal attractiveness is simply proportional to size.

The damping parameter *λ* is set to 0.85, just as in the PageRank algorithm at the basis of the Google search engine; see Brin and Page (1998). The leading eigenvector ***ψ*** of **P** corresponds to the steady state fraction of time spent reading each journal. The eigenfactor score ${\text{EF}}_{i}$ for journal *i* is defined as ‘the percentage of the total weighted citations that journal *i* receives’, i.e.

$${\text{EF}}_{i}=100\frac{{\left[\tilde{C}\psi \right]}_{i}}{\sum_{i=1}^{n}{\left[\tilde{C}\psi \right]}_{i}},\;i=1,\ldots ,n,$$

where ${\left[x\right]}_{i}$ denotes the *i*th element of vector **x**. See www.eigenfactor.org/methods.pdf for more details of the methodology behind the eigenfactor algorithm.

The eigenfactor ‘measures the total influence of a journal on the scholarly literature’ (Bergstrom, 2007) and thus it depends on the number of articles that are published by a journal. The article influence score ${\text{AI}}_{i}$ of journal *i* is instead a measure of the per‐article citation influence of the journal, obtained by normalizing the eigenfactor as follows:

$${\text{AI}}_{i}=0.01\frac{{\text{EF}}_{i}}{a_{i}},\;i=1,\ldots ,n.$$

Distinctive aspects of the article influence score with respect to the impact factor are

- the use of a formal stochastic model to derive the journal ranking and
- the use of bivariate data—the cross‐citations $c_{ij}$—in contrast with the univariate citation counts that are used by the impact factor.

An appealing feature of the article influence score is that citations are weighted according to the importance of the source, whereas the impact factor counts all citations equally (Franceschet, 2010). Accordingly, the bibliometric literature classifies the article influence score as a measure of journal ‘prestige’ and the impact factor as a measure of journal ‘popularity’ (Bollen *et al*., 2006). Table 3 summarizes some of the main features of the ranking methods that are discussed in this section and also of the Stigler model that will be discussed in Section 5 below.

**Table 3:** Characteristics of the journal rankings derived from the JCR†

*Ranking*	*Citation period*	*Stochastic*	*Data*	*Excludes*	*Global or*
	*(years)*	*model*		*self‐citation*	*local*
II	1	None	Univariate	No	Global
IF	2	None	Univariate	No	Global
IFno	2	None	Univariate	Yes	Global
IF5	5	None	Univariate	No	Global
AI	5	Markov process	Bivariate	Yes	Global
SM	10	Bradley–Terry	Bivariate	Yes	Local

†Rankings are the immediacy index II, impact factor IF, impact factor without self‐citations, IFno, 5‐year impact factor, IF5, article influence score AI and the Stigler model studied in this paper, SM. The ‘Data’ column indicates whether the data used are bivariate cross‐citation counts or only univariate citation counts. ‘Global or local’ relates to whether a ranking is ‘local’ to the main journals of statistics, or ‘global’ in that it is applied across disciplines.

The rankings of the selected statistics journals according to impact factor, impact factor without journal self‐citations, 5‐year impact factor, immediacy index and article influence score are reported in the second to sixth columns of Table 4. The substantial variation between those five rankings is the first aspect that leaps to the eye; these different published measures clearly do not yield a common, unambiguous picture of the journals' relative standings.

**Table 4:**
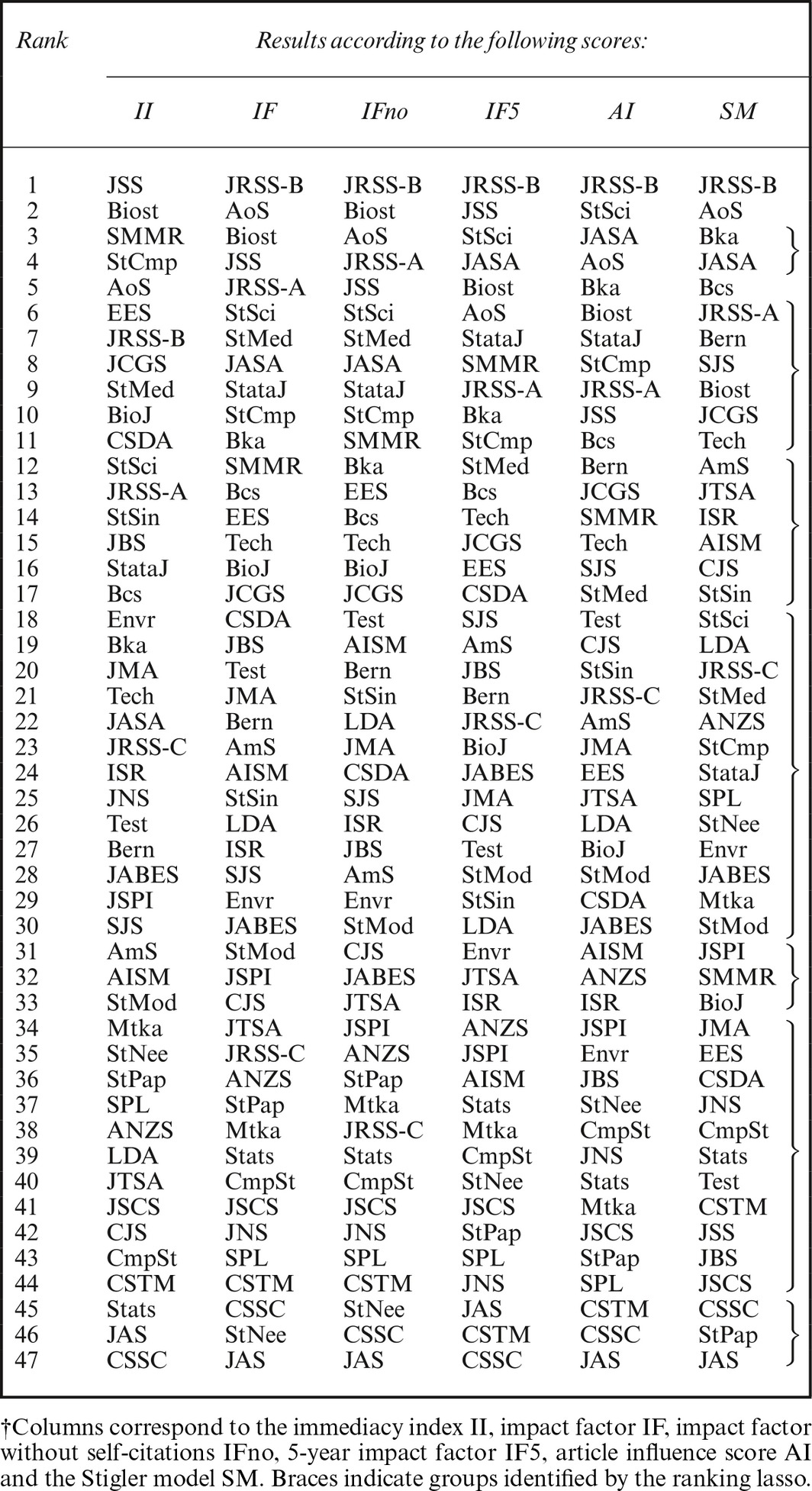
Rankings of selected statistics journals based on the JCR, 2010 edition†

A diffuse opinion within the statistical community is that the four most prestigious statistics journals are (in alphabetic order) *Annals of Statistics*,* Biometrika*, the *Journal of the American Statistical Association* and the *Journal of the Royal Statistical Society*, Series B. See, for example, the survey about how statisticians perceive statistics journals that is described in Theoharakis and Skordia (2003). Accordingly, a minimal requirement for a ranking of acceptable quality is that the four most prestigious journals should occupy prominent positions. Following this criterion, the least satisfactory ranking is, as expected, that based on the immediacy index, which ranks the *Journal of the American Statistical Association* only 22nd and *Biometrika* just a few positions ahead at 19th.

In the three versions of impact factor ranking, the *Journal of the Royal Statistical Society*, Series B, always occupies first position, the *Annals of Statistics* ranges between second and sixth, the *Journal of the American Statistical Association* between fourth and eighth, and *Biometrika* between 10th and 12th. The two software journals have quite high impact factors: the *Journal of Statistical Software* is ranked between second and fifth by the three different impact factor versions, whereas *Stata Journal* is between seventh and ninth. Other journals ranked highly according to the impact factor measures are *Biostatistics* and *Statistical Science*.

Among the indices that are published by Thomson Reuters, the article influence score yields the most satisfactory ranking with respect to the four leading journals mentioned above, all of which stand within the first five positions.

All the indices discussed in this section are constructed by using the complete *Web of Science* database, thus counting citations from journals in other fields as well as citations between statistics and probability journals.

## 5 The Stigler model

Stigler (1994) considered the export of intellectual influence from a journal to determine its importance. The export of influence is measured through the citations that are received by the journal. Stigler assumed that the log‐odds that journal *i* exports to journal *j* rather than vice versa are equal to the difference of the journals' *export scores*,

(2) $$\text{log‐odds}\left(\text{journal}\;\;i\;\;\text{is cited by journal}\;j\right)=\mu_{i}-\mu_{j},$$

where $\mu_{i}$ is the export score of journal *i*. In Stephen Stigler's words ‘the larger the export score, the greater the propensity to export intellectual influence’. The Stigler model is an example of the Bradley–Terry model (Bradley and Terry, 1952; David, 1963; Agresti, 2013) for paired comparison data. According to equation (2), the citation counts $c_{ij}$ are realizations of binomial variables $C_{ij}$ with expected value

(3) $$E\left(C_{ij}\right)=t_{ij}\pi_{ij},$$

where $\pi_{ij}=\exp\left(\mu_{i}-\mu_{j}\right)/\left\{1+\exp\left(\mu_{i}-\mu_{j}\right)\right\}$ and $t_{ij}$ is the total number of citations exchanged between journals *i* and *j*, as defined in equation (1).

The Stigler model has some attractive features.

- *Statistical modelling*: similarly to the eigenfactor and the derived article influence score, the Stigler method is based on stochastic modelling of a matrix of cross‐citation counts. The methods differ regarding the modelling perspective— a Markov process for the eigenfactor *versus* a Bradley–Terry model in the Stigler method—and, perhaps most importantly, the use of formal statistical methods. The Stigler model is calibrated through well‐established statistical fitting methods, such as maximum likelihood or quasi‐likelihood (see Section 5.1), with estimation uncertainty summarized accordingly (Section 5.3). Moreover, Stigler model assumptions are readily checked by the analysis of suitably defined residuals, as described in Section 5.2
- *The size of the journals is not important*. Rankings based on the Stigler model are not affected by the numbers of papers published. As shown by Stigler (1994), page 102, if two journals are merged into a single journal then the odds in favour of that ‘super’ journal against any third journal is a weighted average of the odds for the two separate journals against the third. Normalization for journal size, which is explicit in the definitions of various impact factor and article influence measures, is thus implicit for the Stigler model.
- *Journal self‐citations are not counted*. In contrast with the standard impact factor, rankings based on journal export scores $\mu_{i}$ are not affected by the risk of manipulation through journal self‐citations.
- *Only citations between journals under comparison are counted*. If the Stigler model is applied to the list of 47 statistics journals, then only citations between these journals are counted. Such an application of the Stigler model thus aims unambiguously to measure influence within the research field of statistics, rather than combining that with potential influence on other research fields. As noted in Table 3, this property differentiates the Stigler model from the other ranking indices published by Thomson Reuters, which use citations from all journals in potentially any fields to create a ‘global’ ranking of all scholarly journals. Obviously it would be possible also to recompute more ‘locally’ the various impact factor measures and/or eigenfactor‐based indices, by using only citations exchanged between the journals in a restricted set to be compared.
- *The citing journal is taken into account*. Like the article influence score, the Stigler model measures journals' relative prestige, because it is derived from bivariate citation counts and thus takes into account the source of each citation. The Stigler model decomposes the cross‐citation matrix **C** differently, though; it can be re‐expressed in log‐linear form as the ‘quasi‐symmetry’ model,
  (4) $$E\left(C_{ij}\right)=t_{ij}\text{exp}\left(\alpha_{i}+\beta_{j}\right),$$
  in which the export score for journal *i* is $\mu_{i}=\alpha_{i}-\beta_{i}$.
- *Lack‐of‐fit assessment*: Stigler *et al*. (1995) and Liner and Amin (2004) observed increasing lack of fit of the Stigler model when additional journals that trade little with those already under comparison are included in the analysis. Ritzberger (2008) stated bluntly that the Stigler model ‘suffers from a lack of fit’ and dismissed it—incorrectly, in our view—for that reason. We agree instead with Liner and Amin (2004) who suggested that statistical lack‐of‐fit assessment is another positive feature of the Stigler model that can be used, for example, to identify groups of journals belonging to different research fields, journals which should perhaps not be ranked together. Certainly the existence of principled lack‐of‐fit assessment for the Stigler model should not be a reason to prefer other methods for which no such assessment is available.

See also Table 3 for a comparison of properties of the ranking methods that are considered in this paper.

### 5.1 Model fitting

Maximum likelihood estimation of the vector of journal export scores $\mu ={\left(\mu_{1},\ldots ,\mu_{n}\right)}^{T}$ can be obtained through standard software for fitting generalized linear models. Alternatively, specialized software such as the R package BradleyTerry2 (Turner and Firth, 2012) is available through the Comprehensive R Archive Network repository. Since the Stigler model is specified through pairwise differences of export scores $\mu_{i}-\mu_{j}$, model identification requires a constraint, such as a ‘reference journal’ constraint $\mu_{1}=0$ or the sum constraint $\Sigma_{i=1}^{n}\mu_{i}=0$. Without loss of generality we use the latter constraint in what follows.

Standard maximum likelihood estimation of the Stigler model would assume that citation counts $c_{ij}$ are realizations of independent binomial variables $C_{ij}$. Such an assumption is likely to be inappropriate, since research citations are not independent of one another in practice; see Cattelan (2012) for a general discussion on handling dependence in paired comparison modelling. The presence of dependence between citations can be expected to lead to the well‐known phenomenon of overdispersion. A simple way to deal with overdispersion is provided by the method of quasi‐likelihood (Wedderburn, 1974). Accordingly, we consider a ‘quasi‐Stigler’ model,

(5) $$\begin{array}{cc} & E\left(C_{ij}\right)=t_{ij}\pi_{ij},\\ & \text{var}\left(C_{ij}\right)=\phi t_{ij}\pi_{ij}\left(1-\pi_{ij}\right), \end{array}$$

where *ϕ*>0 is the dispersion parameter. Let **c** be the vector that is obtained by stacking all citation counts $c_{ij}$ in some arbitrary order, and let **t** and ***π*** be the corresponding vectors of totals $t_{ij}$ and expected values $\pi_{ij}$ respectively. Then estimates of the export scores are obtained by solving the quasi‐likelihood estimating equations

(6) $$D^{T}V^{-1}\left(c-t\pi \right)=0,$$

where **D** is the Jacobian of ***π*** with respect to the export scores ***μ***, and **V**=**V**(***μ***) is the diagonal matrix with elements $\text{var}(C_{ij})/\phi$. Under the assumed model (5), quasi‐likelihood estimators are consistent and asymptotically normally distributed with variance–covariance matrix $\phi {\left(D^{T}V^{-1}D\right)}^{-1}$. The dispersion parameter is usually estimated via the squared Pearson residuals as

(7) $$\hat{\phi }=\frac{1}{m-n+1}\sum_{i<j}^{n}\frac{{\left(c_{ij}-t_{ij}{\hat{\pi }}_{ij}\right)}^{2}}{t_{ij}{\hat{\pi }}_{ij}\left(1-{\hat{\pi }}_{ij}\right)},$$

where $\hat{\pi }$ is the vector of estimates ${\hat{\pi }}_{ij}=\exp\left({\hat{\mu }}_{i}-{\hat{\mu }}_{j}\right)/\left\{1+\exp\left({\hat{\mu }}_{i}-{\hat{\mu }}_{j}\right)\right\}$, with ${\hat{\mu }}_{i}$ being the quasi‐likelihood estimate of the export score $\mu_{i}$, and $m=\Sigma_{i<j}1\left(t_{ij}>0\right)$ the number of pairs of journals that exchange citations. Well‐known properties of quasi‐likelihood estimation are robustness against misspecification of the variance matrix **V** and optimality within the class of linear unbiased estimating equations.

The estimate of the dispersion parameter that is obtained here, for the model applied to statistics journal cross‐citations between 2001 and 2010, is $\hat{\phi }=1.76$, indicative of overdispersion. The quasi‐likelihood estimated export scores of the statistics journals are reported in Table 5 and will be discussed later in Section 5.4

**Table 5:** Journal ranking based on the Stigler model using data from the JCR 2010 edition†

*Rank*	*Journal*	*SM*	*QSE*	*SMgrouped*	*Rank*	*Journal*	*SM*	*QSE*	*SMgrouped*
1	JRSS‐B	2.09	0.11	1.87	25	SPL	−0.09	0.09	−0.04
2	AoS	1.38	0.07	1.17	26	StNee	−0.10	0.25	−0.04
3	Bka	1.29	0.08	1.11	27	Envr	−0.11	0.18	−0.04
4	JASA	1.26	0.06	1.11	28	JABES	−0.16	0.23	−0.04
5	Bcs	0.85	0.07	0.65	29	Mtka	−0.18	0.17	−0.04
6	JRSS‐A	0.70	0.19	0.31	30	StMod	−0.22	0.21	−0.04
7	Bern	0.69	0.15	0.31	31	JSPI	−0.33	0.07	−0.31
8	SJS	0.66	0.12	0.31	32	SMMR	−0.35	0.16	−0.31
9	Biost	0.66	0.11	0.31	33	BioJ	−0.40	0.12	−0.31
10	JCGS	0.64	0.12	0.31	34	JMA	−0.45	0.08	−0.36
11	Tech	0.53	0.15	0.31	35	EES	−0.48	0.25	−0.36
12	AmS	0.40	0.18	0.04	36	CSDA	−0.52	0.07	−0.36
13	JTSA	0.37	0.20	0.04	37	JNS	−0.53	0.15	−0.36
14	ISR	0.33	0.25	0.04	38	CmpSt	−0.64	0.22	−0.36
15	AISM	0.32	0.16	0.04	39	Stats	−0.65	0.18	−0.36
16	CJS	0.30	0.14	0.04	40	Test	−0.70	0.15	−0.36
17	StSin	0.29	0.09	0.04	41	CSTM	−0.74	0.10	−0.36
18	StSci	0.11	0.11	−0.04	42	JSS	−0.80	0.19	−0.36
19	LDA	0.10	0.17	−0.04	43	JBS	−0.83	0.16	−0.36
20	JRSS‐C	0.09	0.15	−0.04	44	JSCS	−0.92	0.15	−0.36
21	StMed	0.06	0.07	−0.04	45	CSSC	−1.26	0.14	−0.88
22	ANZS	0.06	0.21	−0.04	46	StPap	−1.35	0.20	−0.88
23	StCmp	0.04	0.15	−0.04	47	JAS	−1.41	0.15	−0.88
24	StataJ	0.02	0.33	−0.04					

†Columns are the quasi‐likelihood estimated Stigler model export scores SM with associated quasi‐standard errors QSE, and estimated export scores after grouping by lasso, SMgrouped.

### 5.2 Model validation

An essential feature of the Stigler model is that the export score of any journal is a constant. In particular, in model (2) the export score of journal *i* is not affected by the identity of the citing journal *j*. Citations that are exchanged between journals can be seen as results of contests between opposing journals and the residuals for contests involving journal *i* should not exhibit any relationship with the corresponding estimated export scores of the ‘opponent’ journals *j*. With this in mind, we define the *journal residual* $r_{i}$ for journal *i* as the standardized regression coefficient derived from the linear regression of Pearson residuals involving journal *i* on the estimated export scores of the corresponding opponent journals. More precisely, the *i*th journal residual is defined here as

$$r_{i}=\frac{\sum_{j=1}^{n}{\hat{\mu }}_{j}r_{ij}}{\surd (\hat{\phi }\sum_{j=1}^{n}{\hat{\mu }}_{j}^{2})},$$

where $r_{ij}$ is the Pearson residual for citations of *i* by *j*,

$$r_{ij}=\frac{c_{ij}-t_{ij}{\hat{\pi }}_{ij}}{\surd \{t_{ij}{\hat{\pi }}_{ij}\left(1-{\hat{\pi }}_{ij}\right)\}}.$$

The journal residual $r_{i}$ indicates the extent to which *i* performs systematically better than predicted by the model either when the opponent *j* is strong, as indicated by a positive‐valued journal residual for *i*, or when the opponent *j* is weak, as indicated by a negative‐valued journal residual for *i*. The journal residuals thus provide a basis for useful diagnostics, targeted specifically at readily interpretable departures from the model assumed.

**Figure 3:**
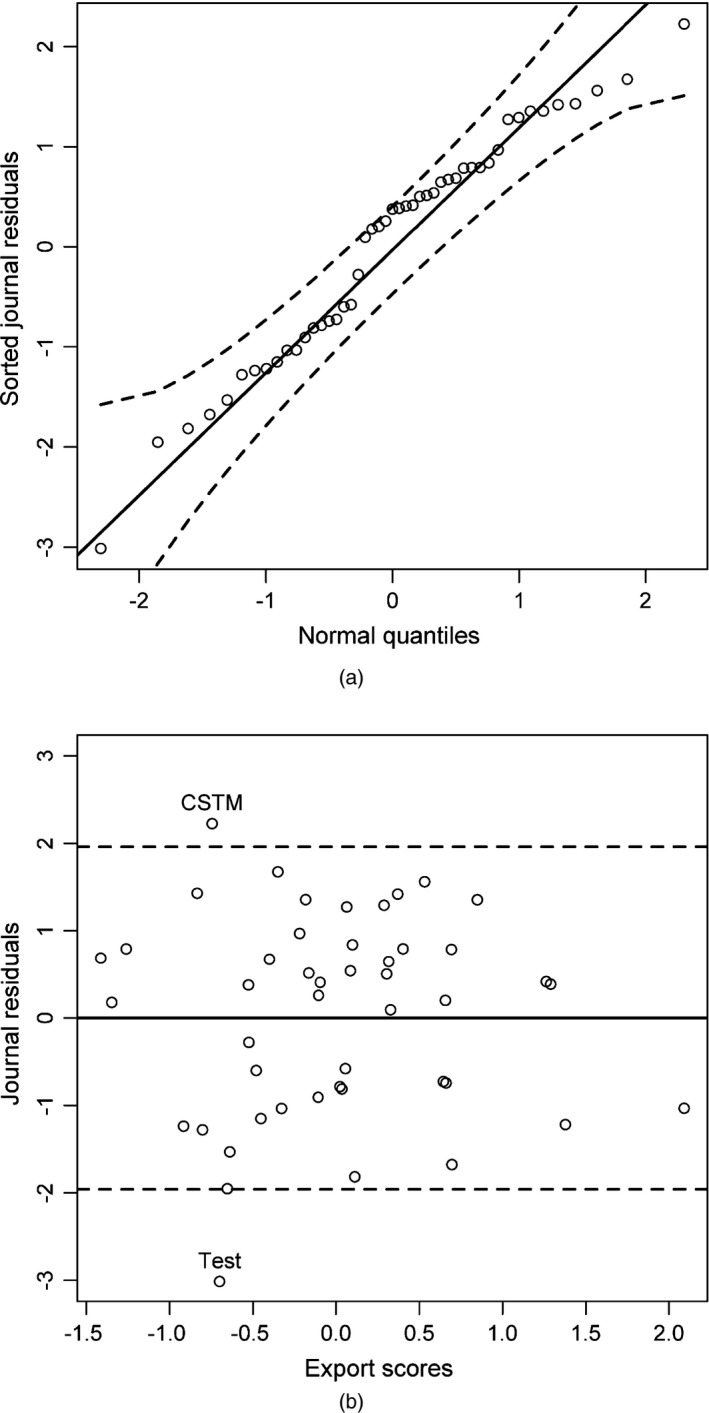
(a) Normal probability plot of journal residuals with 95% simulation envelope and (b) scatter plot of journal residuals *versus* estimated journal export scores

Under the assumed quasi‐Stigler model, journal residuals are approximately realizations of standard normal variables and are unrelated to the export scores. The normal probability plot of the journal residuals displayed in Fig. 3(a) indicates that the normality assumption is indeed approximately satisfied. The scatter plot of the journal residuals $r_{i}$ against estimated export scores ${\hat{\mu }}_{i}$ in Fig. 3(b) shows no clear pattern; there is no evidence of correlation between journal residuals and export scores. As expected on the basis of approximate normality of the residuals, only two journals—i.e. 4.3% of journals—have residuals that are larger in absolute value than 1.96. These journals are *Communications in Statistics—Theory and Methods* ($r_{\mathrm{CSTM}}=2.23$) and *Test* ($r_{\mathrm{Test}}=-3.01$). The overall conclusion from this graphical inspection of journal residuals is that the assumptions of the quasi‐Stigler model appear to be essentially satisfied for the data that are used here.

### 5.3 Estimation uncertainty

Estimation uncertainty is commonly unexplored, and is rarely reported, in relation to the various published journal rankings. Despite this lacuna, many academics have produced vibrant critiques of ‘statistical citation analyses’, although such analyses are actually rather non‐statistical. Recent research in the bibliometric field has suggested that uncertainty in estimated journal ratings might be estimated via bootstrap simulation; see the already mentioned Chen *et al*. (2014) and the ‘stability intervals’ for the source‐normalized impact per paper index. A key advantage of the Stigler model over other ranking methods is straightforward quantification of the uncertainty in journal export scores.

Since the Stigler model is identified through pairwise differences, uncertainty quantification requires the complete variance matrix of $\hat{\mu }$. Routine reporting of such a large variance matrix is impracticable for brevity. A neat solution is provided through the presentational device of quasi‐variances (Firth and de Menezes, 2005), constructed in such a way as to allow approximate calculation of any variance of a difference, $\text{var}({\hat{\mu }}_{i}-{\hat{\mu }}_{j})$, as if ${\hat{\mu }}_{i}$ and ${\hat{\mu }}_{j}$ were independent:

$$\text{var}\left({\hat{\mu }}_{i}-{\hat{\mu }}_{j}\right)≃{\text{qvar}}_{i}+{\text{qvar}}_{j},\text{for all choices of}\;\;i\;\text{and}\;j.$$

Reporting the estimated export scores with their quasi‐variances, then, is an economical way to allow approximate inference on the significance of the difference between any two journals' export scores. The quasi‐variances are computed by minimizing a suitable penalty function of the differences between the true variances, $\text{var}({\hat{\mu }}_{i}-{\hat{\mu }}_{j})$, and their quasi‐variance representations ${\text{qvar}}_{i}+{\text{qvar}}_{j}$. See Firth and de Menezes (2005) for details.

Table 5 reports the estimated journal export scores computed under the sum constraint $\Sigma_{i=1}^{n}\mu_{i}=0$ and the corresponding quasi‐standard errors, defined as the square root of the quasi‐variances. Quasi‐variances are calculated by using the R package qvcalc (Firth, 2012). For illustration, consider testing whether the export score of *Biometrika* is significantly different from that of the *Journal of the American Statistical Association*. The *z*‐test statistic as approximated through the quasi‐variances is

$$z≃\frac{{\hat{\mu }}_{\mathrm{Bka}}-{\hat{\mu }}_{\mathrm{JASA}}}{\surd \left({\text{qvar}}_{\mathrm{Bka}}+{\text{qvar}}_{\mathrm{JASA}}\right)}=\frac{1.29-1.26}{\surd \left(0.08^{2}+0.06^{2}\right)}=0.30.$$

The ‘usual’ variances for those two export scores in the sum‐constrained parameterization are respectively 0.0376 and 0.0344, and the covariance is 0.0312; thus the ‘exact’ value of the *z*‐statistic in this example is

$$z=\frac{1.29-1.26}{\surd \left\{0.0376-2(0.0312)+0.0344\right\}}=0.31,$$

so the approximation based on quasi‐variances is quite accurate. In this case the *z*‐statistic suggests that there is insufficient evidence to rule out the possibility that *Biometrika* and the *Journal of the American Statistical Association* have the same ability to ‘export intellectual influence’ within the 47 statistics journals in the list.

### 5.4 Results

We proceed now with interpretation of the ranking based on the Stigler model. It is reassuring that the four leading statistics journals that were mentioned previously are ranked in the first four positions. The *Journal of the Royal Statistical Society*, Series B, is ranked first with a remarkably larger export score than the second‐ranked journal, the *Annals of Statistics*: the approximate *z*‐statistic for the significance of the difference of their export scores is 5.44. The third position is occupied by *Biometrika*, closely followed by the *Journal of the American Statistical Association*.

The fifth‐ranked journal is *Biometrics*, followed by the *Journal of the Royal Statistical Society*, Series A, *Bernoulli*, the *Scandinavian Journal of Statistics*,* Biostatistics*, the *Journal of Computational and Graphical Statistics* and *Technometrics*.

**Figure 4:**
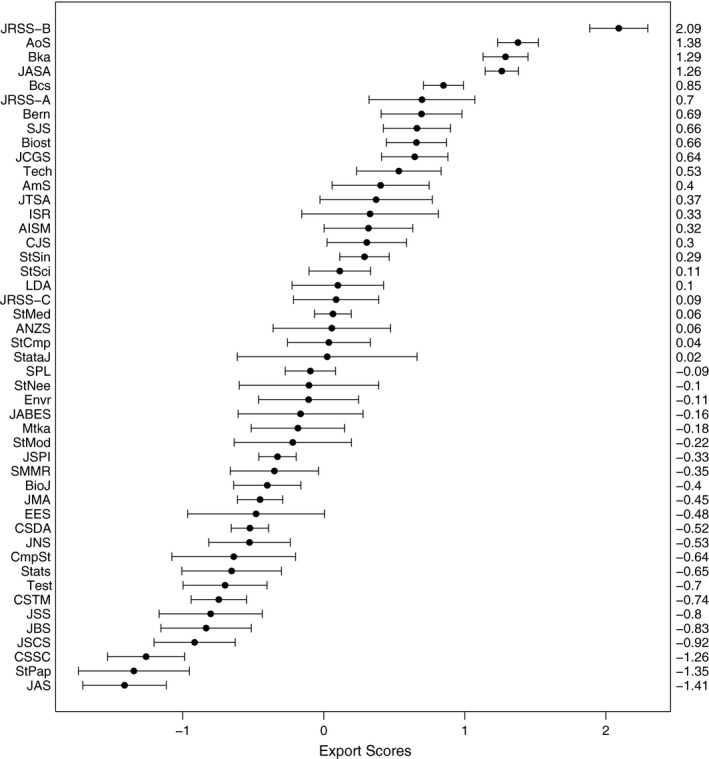
Centipede plot of estimated journal export scores and 95% comparison intervals based on the JCR 2010 edition: the error bar limits are ${\hat{\mu }}_{i}\pm 1.96\;\text{QSE}\left({\hat{\mu }}_{i}\right)$, with the estimated export scores ${\hat{\mu }}_{i}$ marked (•)

The ‘centipede’ plot in Fig. 4 visualizes the estimated export scores along with the 95% comparison intervals with limits ${\hat{\mu }}_{i}\pm 1.96\text{QSE}\left({\hat{\mu }}_{i}\right)$, where ‘QSE’ denotes the quasi‐standard‐error. The centipede plot highlights the outstanding position of the *Journal of the Royal Statistical Society*, Series B, and indeed of the four top journals whose comparison intervals are well separated from those of the remaining journals. However, the most striking general feature is the substantial uncertainty in most of the estimated journal scores. Many of the small differences that appear between the estimated export scores are not statistically significant.

### 5.5 Ranking in groups with lasso

Shrinkage estimation offers notable improvement over standard maximum likelihood estimation when the target is simultaneous estimation of a vector of mean parameters; see, for example, Morris (1983). It seems natural to consider shrinkage estimation also for the Stigler model. Masarotto and Varin (2012) fitted Bradley–Terry models with a lasso‐type penalty (Tibshirani, 1996) which, in our application here, forces journals with close export scores to be estimated at the same level. The method, which is termed the ranking lasso, has the twofold advantages of shrinkage and enhanced interpretation, because it avoids overinterpretation of small differences between estimated journal export scores.

For a given value of a bound parameter *s*⩾0, the ranking lasso method fits the Stigler model by solving the quasi‐likelihood equations (6) with an $L_{1}$‐penalty on all the pairwise differences of export scores, i.e

(8) $$D^{T}V^{-1}\left(c-t\pi \right)=0,\text{subject to}\sum_{i<j}^{n}w_{ij}\left|\mu_{i}-\mu_{j}\right|⩽s\;\text{and}\;\sum_{i=1}^{n}\mu_{i}=0,$$

where the $w_{ij}$ are data‐dependent weights discussed below.

Quasi‐likelihood estimation is obtained for a sufficiently large value of the bound *s*. As *s* decreases to 0, the $L_{1}$‐penalty causes journal export scores that differ little to be estimated at the same value, thus producing a ranking in groups. The ranking lasso method can be interpreted as a generalized version of the fused lasso (Tibshirani *et al*., 2005).

Since quasi‐likelihood estimates coincide with maximum likelihood estimates for the corresponding exponential dispersion model, ranking lasso solutions can be computed as penalized likelihood estimates. Masarotto and Varin (2012) obtained estimates of the adaptive ranking lasso by using an augmented Lagrangian algorithm (Nocedal and Wright, 2006) for a sequence of bounds *s* ranging from complete shrinkage (*s*=0)—i.e. all journals have the same estimated export score—to the quasi‐likelihood solution (*s*=∞).

Many researchers (e.g. Fan and Li (2001) and Zou (2006)) have observed that lasso‐type penalties may be too severe, thus yielding inconsistent estimates of the non‐zero effects. In the ranking lasso context, this means that, if the weights $w_{ij}$ in problem (8) are all identical, then the pairwise differences $\mu_{i}-\mu_{j}$ whose ‘true’ value is non‐zero might not be consistently estimated. Among various possibilities, an effective way to overcome the drawback is to resort to the adaptive lasso method (Zou, 2006), which imposes a heavier penalty on small effects. Accordingly, the adaptive ranking lasso employs weights that are equal to the reciprocal of a consistent estimate of $\mu_{i}-\mu_{j}$, such as $w_{ij}={\left|{\hat{\mu }}_{i}^{\left(\mathrm{QLE}\right)}-{\hat{\mu }}_{j}^{\left(\mathrm{QLE}\right)}\right|}^{-1},$ with ${\hat{\mu }}_{i}^{\left(\mathrm{QLE}\right)}$ being the quasi‐likelihood estimate of the export score for journal *i*.

Lasso tuning parameters are often determined by cross‐validation. Unfortunately, the interjournal ‘tournament’ structure of the data does not allow the identification of internal replication; hence it is not clear how cross‐validation can be applied to citation data. Alternatively, tuning parameters can be determined by minimization of suitable information criteria. The usual Akaike information criterion is not valid with quasi‐likelihood estimation because the likelihood function is formally unspecified. A valid alternative is based on the Takeuchi information criterion TIC (Takeuchi, 1976) which extends the Akaike information criterion when the likelihood function is misspecified. Let $\hat{\mu }\left(s\right)={\left({\hat{\mu }}_{1}\left(s\right),\ldots ,{\hat{\mu }}_{n}\left(s\right)\right)}^{T}$ denote the solution of problem (8) for a given value of the bound *s*. Then the optimal value for *s* is chosen by minimization of

$$\text{TIC}\left(s\right)=-2\hat{l}\left(s\right)+2\text{tr}\{J\left(s\right)I{\left(s\right)}^{-1}\}\;,$$

where $\hat{l}\left(s\right)=l\left\{\hat{\mu }\left(s\right)\right\}$ is the misspecified log‐likelihood of the Stigler model

$$l\left(\mu \right)=\sum_{i<j}^{n}c_{ij}\left(\mu_{i}-\mu_{j}\right)-t_{ij}\ln\left\{1+\exp\left(\mu_{i}-\mu_{j}\right)\right\}$$

computed at $\hat{\mu }\left(s\right)$, ${J\left(s\right)=\text{var}\left\{\nabla l\left(\mu \right)\right\}|}_{\mu =\hat{\mu }\left(s\right)}$ and $I\left(s\right)=-E\left\{\nabla^{2}l\left(\mu \right)\right\}{|}_{\mu =\hat{\mu }\left(s\right)}$. Under the assumed quasi‐Stigler model, **J**(*s*)=*ϕ* **I**(*s*) and the TIC‐statistic reduces to

$$\text{TIC}\left(s\right)=-2\hat{l}\left(s\right)+2\phi p,$$

where *p* is the number of distinct groups formed with bound *s*. The dispersion parameter *ϕ* can be estimated as in equation (7). The effect of overdispersion is inflation of the Akaike information criterion model dimension penalty.

**Figure 5:**
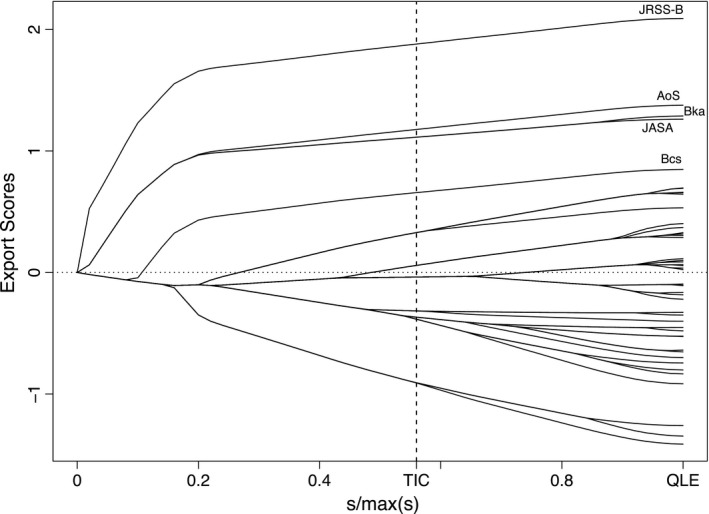
Path plot of adaptive ranking lasso analysis based on the JCR 2010 edition: QLE, quasi‐likelihood estimate, TIC, Takeuchi information criterion

Fig. 5 displays the path plot of the ranking lasso, and Table 5 reports estimated export scores corresponding to the solution identified by TIC. See also Table 4 for a comparison with the Thomson Reuters published rankings. The path plot of Fig. 5 visualizes how the estimates of the export scores vary as the degree of shrinkage decreases, i.e. as the bound *s* increases. The plot confirms the outstanding position of the *Journal of the Royal Statistical Society*, Series B, the leader in the ranking at any level of shrinkage. Also *Annals of Statistics* keeps the second position for about three‐quarters of the path before joining the paths of *Biometrika* and the *Journal of the American Statistical Association*. *Biometrics* is solitary in fifth position for almost the whole of its path. The TIC‐statistic identifies a sparse solution with only 10 groups. According to TIC, the five top journals are followed by a group of six further journals, namely the *Journal of the Royal Statistical Society*, Series A, *Bernoulli*, the *Scandinavian Journal of Statistics*,* Biostatistics*, the *Journal of Computational and Graphical Statistics* and *Technometrics*. However, the main conclusion from this ranking lasso analysis is that many of the estimated journal export scores are not clearly distinguishable from one another.

## 6 Comparison with results from the UK research assessment exercise

### 6.1 Background

In the UK, the quality of the research that is carried out in universities is assessed periodically by the government‐supported funding councils, as a primary basis for future funding allocations. At the time of writing, the most recent such assessment to be completed was the 2008 RAE, full details of which are on line at www.rae.ac.uk. The next such assessment to report, at the end of 2014, will be the similar ‘research excellence framework’. Each unit of assessment is an academic ‘department’, corresponding to a specified research discipline. In the 2008 RAE, ‘Statistics and operational research’ was one of 67 such research disciplines; in contrast the 2014 research excellence framework has only 36 separate discipline areas identified for assessment, and research in statistics will be part of a new and much larger ‘Mathematical sciences’ unit of assessment. The results from the 2008 RAE are therefore likely to provide the last opportunity to make a directly statistics‐focused comparison with journal rankings.

The word ‘department’ in the 2008 RAE refers to a discipline‐specific group of researchers submitted for assessment by a university, or sometimes by two universities together: a department in the 2008 RAE need not be an established academic unit within a university, and indeed many of the 2008 RAE statistics and operational research departments were actually groups of researchers working in university departments of mathematics or other disciplines.

It is often argued that the substantial cost of assessing research outputs through review by a panel of experts, as was done in the 2008 RAE, might be reduced by employing suitable metrics based on citation data. See, for example, Jump (2014). Here we briefly explore this in quite a specific way, through data on journals rather than on the citations that are attracted by individual research papers submitted for assessment.

The comparisons to be made here can also be viewed as exploring an aspect of ‘criterion validity’ of the various journal ranking methods: if highly ranked journals tend to contain high quality research, then there should be evidence through strong correlations, even at the ‘department’ level of aggregation, between expert panel assessments of research quality and journal ranking scores.

### 6.2 Data and methods

We examine only Sub‐panel 22, ‘Statistics and operational research’ of the 2008 RAE. The specific data used here are

- the detailed ‘RA2’ (research outputs) submissions made by departments to the 2008 RAE (these list up to four research outputs per submitted researcher) and
- the published 2008 RAE results on the assessed quality of research outputs, namely the ‘outputs subprofile’ for each department.

From the RA2 data, only research outputs categorized in the 2008 RAE as ‘journal article’ are considered here. For each such article, the journal's name is found in the ‘publisher’ field of the data. A complication is that the name of any given journal can appear in many different ways in the RA2 data, e.g. ‘*Journal of the Royal Statistical Society* B’ and ‘*Journal of the Royal Statistical Society* Series B: Statistical Methodology’, and the International Standard Serial Number codes as entered in the RA2 data are similarly unreliable. Unambiguously resolving all of the many different representations of journal names proved to be the most time‐consuming part of the comparison exercise that is reported here.

The 2008 RAE outputs subprofile for each department gives the assessed percentage of research outputs at each of five quality levels, these being ‘world leading’ (shorthand code ‘4${}^{*}$’), ‘internationally excellent’ (shorthand ‘3${}^{*}$’), then ‘2${}^{*}$’, ‘1${}^{*}$’ and ‘U’ (unclassified). For example, the outputs subprofile for University of Oxford, the highest‐rated statistics and operational research submission in the 2008 RAE, is

$$\left.\begin{array}{ccccc} 4^{*} & 3^{*} & 2^{*} & 1^{*} & U^{*}\\ 37.0 & 49.5 & 11.4 & 2.1 & 0. \end{array}\right.$$

Our focus will be on the fractions at the 4${}^{*}$ and 3${}^{*}$ quality levels, since those are used as the basis for research funding. Specifically, in the comparisons that are made here the RAE ‘score’ used will be the percentage at 4${}^{*}$ plus a third of the percentage at 3${}^{*}$, computed from each department's 2008 RAE outputs subprofile. Thus, for example, Oxford's 2008 RAE score is calculated as 37.0+49.5/3=53.5. This scoring formula is essentially that used since 2010 to determine funding council allocations; we have considered also various other possibilities, such as simply the percentage at $4^{*}$, or the percentage at $3^{*}$ or higher, and found that the results below are not sensitive to this choice.

For each of the journal ranking methods listed in Table 3, a bibliometrics‐based comparator score per department is then constructed in a natural way as follows. Each RAE‐submitted journal article is scored individually, by for example the impact factor of the journal in which it appeared; and those individual article scores are then averaged across all of a department's RAE‐submitted journal articles. For the averaging, we use the simple arithmetic mean of scores; an exception is that Stigler model export scores are exponentiated before averaging, so that they are positive valued like the scores for the other methods considered. Use of the median was considered as an alternative to the mean; it was found to produce very similar results, which accordingly will not be reported here.

A complicating factor for the simple scoring scheme just described is that journal scores were not readily available for all the journals named in the RAE submissions. For the various ‘global’ ranking measures (see Table 3), scores were available for the 110 journals in the JCR ‘Statistics and probability’ category, which covers approximately 70% of the RAE‐submitted journal articles to be scored. For the Stigler model as used in this paper, though, only the subset of 47 statistics journals that are listed in Table 1 are scored; and this subset accounts for just under half of the RAE‐submitted journal articles. In what follows we have ignored all articles that appeared in unscored journals, and used the rest. To enable a more direct comparison with the use of Stigler model scores, for each of the global indices we computed also a restricted version of its mean score for each department, i.e. restricted to using scores for only the 47 statistics journals from Table 1.

Of the 30 departments submitting work in ‘Statistics and operational research’ to the 2008 RAE, four turned out to have substantially less than 50% of their submitted journal articles in the JCR ‘Statistics and probability’ category of journals. The data from those four departments, which were relatively small groups and whose RAE‐submitted work was mainly in operational research, have been omitted from the following analysis.

The statistical methods that are used below to examine department level relationships between the RAE scores and journal‐based scores are simply correlation coefficients and scatter plots. Given the arbitrary nature of data availability for this particular exercise, anything more sophisticated would seem inappropriate.

### 6.3 Results

Table 6 shows, for bibliometrics‐based mean scores based on each of the various journal ranking measures discussed in this paper, the computed correlation with departmental RAE score. The main features of Table 6 are as follows.

**Table 6:** 2008 RAE score for research outputs in 26 UK ‘Statistics and operational research’ departments: Pearson correlation with departmental mean scores derived from the various journal rating indices based on the 2010 JCR

space0pt10pt*Journals scored*	*Results for the following journal scoring methods:*						
	*II*	*IF*	*IFno*	*IF5*	*AI*	*SM*	*SMgrouped*
All of the JCR ‘Statistics	0.34	0.47	0.49	0.50	0.73	—	—
and probability' category							
Only the 47 statistics	0.34	0.69	0.70	0.73	0.79	0.81	0.82
journals listed in Table 2

- The article influence and Stigler model scores correlate more strongly with RAE results than do scores based on the other journal ranking measures.
- The various global measures show stronger correlation with the RAE results when they are used only to score articles from the 47 statistics journals of Table 1, rather than to score everything from the larger set of journals in the JCR ‘Statistics and probability’ category.

The first of these findings unsurprisingly gives clear support to the notion that the use of bivariate citation counts, which take account of the source of each citation and hence lead to measures of journal ‘prestige’ rather than ‘popularity’, is important if a resultant ranking of journals should relate strongly to the perceived quality of published articles. The second finding is more interesting: for good agreement with departmental RAE ratings; it can be substantially better to score only those journals that are in a relatively homogeneous subset than to use all the scores that might be available for a larger set of journals. In the present context, for example, citation patterns for research in probability are known to differ appreciably from those in statistics, and global scoring of journals across these disciplines would tend not to rate highly even the very best work in probability.

**Figure 6:**
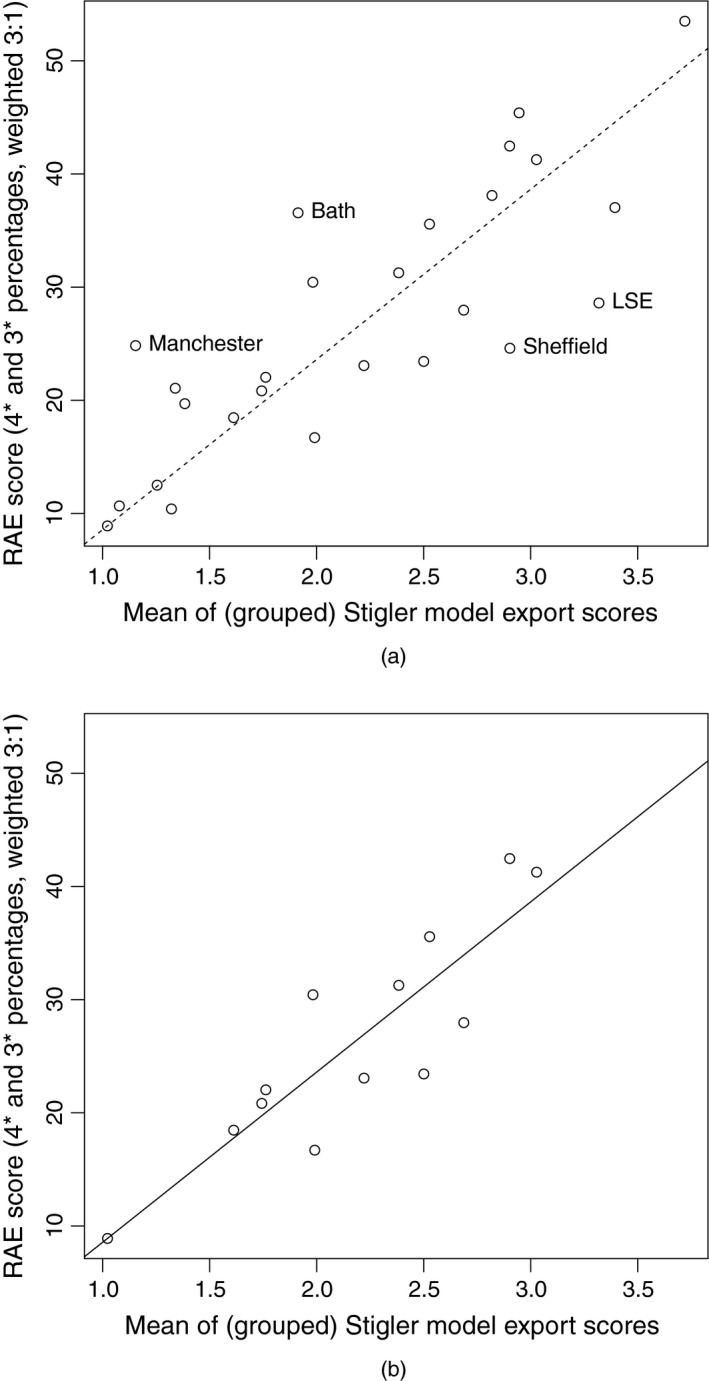
(a) Scatter plot of the 2008 RAE outcome (scores derived from the published RAE ‘outputs’ subprofiles) against averaged Stigler model journal export scores for RAE‐submitted papers (the 26 plotted points are the main ‘Statistics and operational research’ groups at UK universities; four outliers from a straight line fit are highlighted) and (b) a subset of the same scatter plot: just the 13 research groups for which papers published in the 47 journals in Table 1 formed the majority of their RAE‐submitted research outputs; the straight line shown in both panels is the least squares fit to these 13 points

The strongest correlations found in Table 6 are those based on journal export scores from the Stigler model, from columns ‘SM’ and ‘SM grouped’ of Table 5. The departmental means of grouped export scores from the ranking lasso method correlate most strongly with RAE scores, which is a finding that supports the notion that small estimated differences between journals are likely to be spurious. Fig. 6(a) shows the relationship between RAE score and the mean of ‘SM‐grouped’ exponentiated journal export scores, for the 26 departments whose RAE‐submitted journal articles were predominantly in the JCR ‘Statistics and probability’ category; the correlation as reported in Table 6 is 0.82. The four largest outliers from a straight line relationship are identified in the plot, and it is notable that all of those four departments are such that the ratio

(9) $$\frac{\text{number of RAE outputs in the 47 statistics journals of Table 1}}{\text{total number of RAE‐submitted journal articles}}$$

is less than $\frac{1}{2}$. Thus the largest outliers are all departments for which the majority of RAE‐submitted journal articles are not actually scored by our application of the Stigler model, and this seems entirely to be expected. Fig. 6(b) plots the same scores but now omitting all the 13 departments whose ratio (9) is less than $\frac{1}{2}$. The result is, as expected, much closer to a straight line relationship; the correlation in this restricted set of the most ‘statistical’ departments increases to 0.88.

Some brief remarks on interpretation of these findings appear in Section 7.5 below. The data and R language code for this comparison are included in this paper's supplementary Web materials.

## 7 Concluding remarks

### 7.1 The role of statistical modelling in citation analysis

In his Presidential address at the 2011 Institute of Mathematical Statistics Annual Meeting about controversial aspects of measuring research performance through bibliometrics, Professor Peter Hall concluded that

‘As statisticians we should become more involved in these matters than we are. We are often the subject of the analyses discussed above, and almost alone we have the skills to respond to them, for example by developing new methodologies or by pointing out that existing approaches are challenged. To illustrate the fact that issues that are obvious to statisticians are often ignored in bibliometric analysis, I mention that many proponents of impact factors, and other aspects of citation analysis, have little concept of the problems caused by averaging very heavy tailed data. (Citation data are typically of this type.) We should definitely take a greater interest in this area' (Hall, 2011).

The model‐based approach to journal ranking that is discussed in this paper is a contribution in the direction that Professor Hall recommended. Explicit statistical modelling of citation data has two important merits: first, transparency, since model assumptions need to be clearly stated and can be assessed through standard diagnostic tools; secondly, the evaluation and reporting of uncertainty in statistical models can be based on well‐established methods.

### 7.2 The importance of reporting uncertainty in journal rankings

Many journals' Web sites report the latest journal impact factor and the journal's corresponding rank in its category. Very small differences in the reported impact factor often imply large differences in the corresponding rankings of statistics journals. Statisticians should naturally be concerned about whether such differences are significant. Our analyses conclude that many of the apparent differences between estimated export scores are insignificant, and thus differences in journal ranks are often not reliable. The clear difficulty of discriminating between journals on the basis of citation data is further evidence that the use of journal rankings for evaluation of individual researchers will often—and perhaps always—be inappropriate.

In view of the uncertainty in rankings, it makes sense to ask whether the use of ‘grouped’ ranks such as those that emerge from the lasso method of Section 5.5 should be universally advocated. If the rankings or associated scores are to be used for prediction, then the usual arguments for shrinkage methods apply and such grouping, to help to eliminate apparent but spurious differences between journals, is likely to be beneficial; predictions based on grouped ranks or scores are likely to be at least as good as those made without the grouping, as indeed we found in Section 6.3 in connection with the 2008 RAE outcomes. For presentational purposes, though, the key requirement is at least some indication of the amount of uncertainty, and ungrouped estimates coupled with realistically wide intervals, as in the centipede plot of Fig. 4, will often suffice.

### 7.3 A ‘read papers’ effect?

Discussion papers read to the Society at meetings organized by the Research Section of the Royal Statistical Society are a distinctive aspect of the *Journal of the Royal Statistical Society*, Series B. It is natural to ask whether there is a ‘read papers effect’ which might explain the prominence of that journal under the metric used in this paper. During the study period 2001–2010, the *Journal of the Royal Statistical Society*, Series B, published in total 446 articles, 36 of which were papers read to the Society. Half of these papers were published during the three years 2002–2004. The *Journal of the Royal Statistical Society*, Series B, received in total 2554 citations from papers published in 2010, with 1029 of those citations coming from other statistics journals in the list. Despite the fact that papers read to the Society were only 8.1% of all published *Journal of the Royal Statistical Society*, Series B, papers, they accounted for 25.4% (649/2554) of all citations received by the *Journal of the Royal Statistical Society*, Series B, in 2010, and 23.1% (238/1029) of the citations from the other statistics journals in the list.

Papers read to the Society are certainly an important aspect of the success of the *Journal of the Royal Statistical Society*, Series B. However, not all such papers contribute strongly to the citations received by the journal. In fact, a closer look at citation counts reveals that the distribution of the citations received by papers read to the Society is very skew, not differently from what happens for ‘standard’ papers. The most cited read paper published in 2001–2010 was Spiegelhalter *et al*. (2002), which alone received 11.9% of all *Journal of the Royal Statistical Society*, Series B, citations in 2010, and 7.4% of those received from other statistics journals in the list. About 75% of the remaining discussion papers published in the study period each received less than 0.5% of the 2010 *Journal of the Royal Statistical Society*, Series B, citations.

A precise quantification of the ‘read paper’ effect is difficult. Refitting the Stigler model dropping the citations that were received by these papers seems an unfair exercise. Proper evaluation of the effect would require removal also of the citations received by other papers derived from papers read to the Society and published either in the *Journal of the Royal Statistical Society*, Series B, or elsewhere.

### 7.4 Possible extensions

#### 7.4.1 Fractioned citations

The analyses that are discussed in this paper are based on the total numbers $c_{ij}$ of citations exchanged by pairs of journals in a given period and available through the JCRs. One potential drawback of this approach is that citations are all counted equally, irrespective of the number of references contained in the citing paper. Some recent papers in the bibliometric literature (e.g. Zitt and Small (2008), Moed (2010), Leydesdorff and Opthof (2010) and Leydesdorff and Bornmann (2011)) suggest that the impact factor and other citation indices should be recomputed by using fractional counting, in which each citation is counted as 1/*n* with *n* being the number of references in the citing paper. Fractional counting is a natural expedient to take account of varying lengths of reference lists in papers; for example, a typical review article contains many more references than does a short, technical research paper. The Stigler model extends easily to handle such fractional counting, e.g. through the quasi‐symmetry formulation (4); and the rest of the methodology described here would apply with straightforward modifications.

#### 7.4.2 Evolution of export scores

This paper discusses a ‘static’ Stigler model fitted to data extracted from a single JCR edition. A natural extension would be to study the evolution of citation exchange between pairs of journals over several years, through a dynamic version of the Stigler model. A general form for such a model is

$$\text{log‐odds}\left(\text{journal}\;\;i\;\;\text{is cited by journal}\;j\;\text{in year}\;\;t\right)=\mu_{i}\left(t\right)-\mu_{j}\left(t\right),$$

where each journal's time‐dependent export score $\mu_{i}\left(t\right)$ is assumed to be a separate smooth function of *t*. Such a model would not only facilitate the systematic study of time trends in the relative intellectual influence of journals; it would also ‘borrow strength’ across years to help to smooth out spurious variation, whether it be ‘random’ variation arising from the allocation of citing papers to a specific year's JCR edition, or variation caused by transient, idiosyncratic patterns of citation. A variety of such dynamic extensions of the Bradley–Terry model have been developed in other contexts, especially the modelling of sports data; see, for example, Fahrmeir and Tutz (1994), Glickman (1999), Knorr‐Held (2000) and Cattelan *et al*. (2013).

### 7.5 Citation‐based metrics and research assessment

From the strong correlations found in Section 6 between the 2008 RAE outcomes and journal ranking scores, it is tempting to conclude that the expert review element of such a research assessment might reasonably be replaced, mainly or entirely, by automated scoring of journal articles based on the journals in which they have appeared. Certainly Fig. 6 indicates that such scoring, when applied to the main journals of statistics, can perform quite well as a predictor of RAE outcomes for research groups whose publications have appeared mostly in those journals.

The following points should be noted, however.

- Even with correlation as high as 0.88, as in Fig. 6(b), there can be substantial differences between departments' positions based on RAE outcomes and on journal scores. For example, in Fig. 6(b) there are two departments whose mean scores based on our application of the Stigler model are between 1.9 and 2.0 and thus essentially equal, but their computed RAE scores, at 16.7 and 30.4, differ very substantially indeed.
- High correlation was achieved by scoring only a relatively homogeneous subset of all the journals in which the RAE‐submitted work appeared. Scoring a wider set of journals, to cover most or all of the journal articles appearing in the 2008 RAE ‘Statistics and operational research’ submissions, leads to much lower levels of agreement with RAE results.

In relation to point (a) it could of course be argued that, in cases such as the two departments mentioned, the 2008 RAE panel of experts were wrong, or it could be that the difference that was seen between those two departments in the RAE results is largely attributable to the 40% or so of journal articles for each department that were not scored because they were outside the list in Table 1. Point (b), in contrast, seems more clearly to be a severe limitation on the potential use of journal scores in place of expert review. The use of cluster analysis as in Section 3, in conjunction with expert judgements about which journals are ‘core’ to disciplines and subdisciplines, can help to establish relatively homogeneous subsets of journals that might reasonably be ranked together; but comparison across the boundaries of such subsets is much more problematic.

The analysis that is described in this paper concerns journals. It says nothing directly about the possible use of citation data on individual research outputs, as were made available to several of the review panels in the 2014 research excellence framework for example. For research in mathematics or statistics it seems clear that such data on recent publications carry little information, mainly because of long and widely varying times taken for good research to achieve ‘impact’ through citations; indeed, the mathematical sciences subpanel in the 2014 research excellence framework chose not to use such data at all. Our analysis does, however, indicate that any counting of citations to inform assessment of research quality should at least take account of the source of each citation.

## Discussion on the paper by Varin, Cattelan and Firth

**David Colquhoun** (*University College London*)

It is a pleasure to propose the vote of thanks for a paper that puts yet another nail in the coffin of the journal impact factor (JIF).

There are two classes of reasons to deplore JIFs. One is that they are statistically dubious, and that is what Varin and his colleagues develop. It has been obvious for a long time that it is statistically illiterate to characterize very skew distributions by their mean. And it is statistically illiterate to present point estimates with no indication of their uncertainty. The existence of so many different methods for ranking journals, each of which gives different answers, renders them useless.

There are many other reasons for deploring the use of the JIF for assessment of individuals. For a start, it seems self‐evident that individuals should be assessed by what they have written, not via citations of other papers that have appeared in the journals where their papers have appeared. Seglen (1997) pointed out that there is no detectable correlation between the number of citations that a paper receives and the impact factor of the journal in which it appears. That shows that assessing an author on the basis of citations of their papers (itself a dubious process) will give quite a different assessment from assessing them on the basis of the JIF. Recall that Andrew Wakefield's notorious (and fraudulent) 1998 paper has been cited over 760 times.

**Figure 7:**
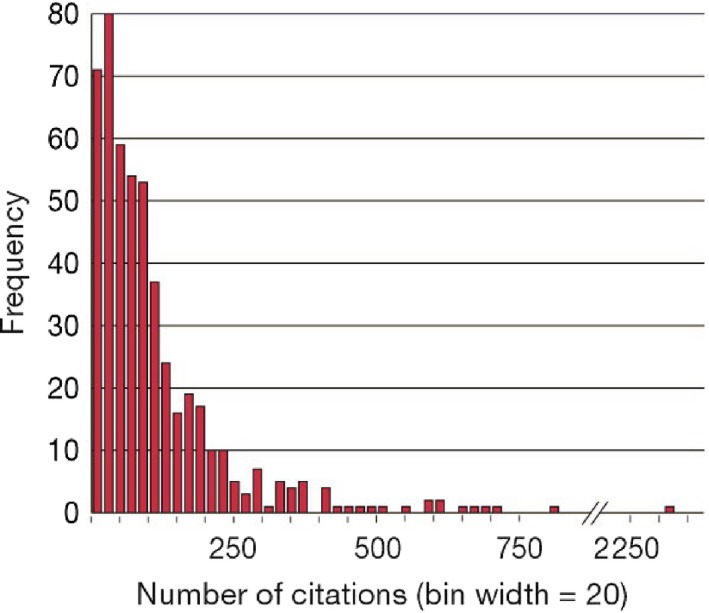
Distribution of the number of citations in 5 years for 500 biomedical papers published in *Nature*: 100 papers published in each of 1981, 1984, 1988, 1992 and 1996 were chosen at random, and for each paper the number of citations in the subsequent 5 years was counted (reproduced from Colquhoun (2003)); the mean number of citations is 114, but the median is only 65; almost 70% of papers have fewer citations than the mean; one paper has 2364 citations but 35 have 10 or fewer; the skewness of the distribution is 4.1 (which is far more skewed than an exponential distribution, which has a skewness of 2) (the data were provided by Grant Lewison, Department of Information Science, City University, London, UK)

Fig. 7 shows a distribution of the number of citations received by 500 biomedical papers that were published in *Nature* (Colquhoun, 2003). Of these 500 papers, 35 were cited fewer than 10 times. It is patently absurd that the papers that were rarely cited should be given credit because a different paper in the same journal was cited 2364 times.

The errors in journal rankings are enormous, as is obvious from Fig. 4, but of course estimates of error are never published. It would have been interesting to see a method like Benjamini and Hochberg's (1995) applied to the 1081 pairwise comparisons between 47 journals that were included in this study.

It is fair to ask what useful facts about journals have been revealed by Varin and his colleagues? We learn that the *Journal of the Royal Statistical Society*, Series B, is quite a good journal. Who would have thought it?

Similar criticisms apply to rankings of universities. The research excellence framework told us, at enormous expense, that Oxford and Cambridge are rather good universities. Many outcomes are measured, so (almost) any university could claim that it was near the top on one of them. But the rankings are based on totally arbitrary weighting of quite different sorts of input to derive a single number to be ranked (Times Higher Education, 2015).

Whoever could imagine that something as complicated as a university could be characterized by a single number? Yet belief in that obvious fallacy has made a fortune for citation companies and purveyors of rankings. And the fact that Vice‐Chancellors seem to fall for the confidence trick has led to the corruption of science by imposition of perverse incentives (Colquhoun, 2014a). It has even led to the occasional death (Colquhoun, 2014b).

The problems become really serious when things like the JIF are used to assess individuals. Only three universities in the UK have signed the San Francisco Declaration on Research Assessment (American Society for Cell Biology, 2015), and there is a widespread belief that even those who have signed it ignore it in practice. Other universities are quite shameless about it. Academics in the Department of Medicine at Imperial College London were told (in 2007) that they are expected to ‘publish three papers per annum, at least one in a prestigious journal with an impact factor of at least five’ (Colquhoun, 2007).

Both journal rankings and university rankings suffer from trying to characterize complex and heterogeneous phenomena in a single number.

The use of the ranking lasso method by Varin and his colleagues shows that even the 47 statistics journals in their study fall into more‐or‐less clear groups. Even in among 47 statistics journals there is heterogeneity.

Normalization of different fields is impossible. I am a pharmacologist–biophysicist and amateur statistician. I would be submitted to the research excellence framework under biology, but quite a few of my papers are mathematical (e.g. Colquhoun *et al*. (1996)). The more mathematical they are, the fewer the citations they receive, despite the fact that they underlie subsequent work.

**Figure 8:**
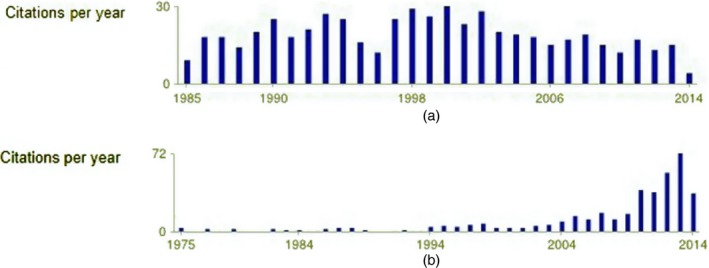
Annual citation rates from Google Scholar for (a) Colquhoun and Hawkes (1995) (cited by 579 since the first edition, 1985) and (b) Hawkes (1971)

The time course of citations varies enormously. Fig. 8 shows two extreme examples. Fig. 8(a) shows citations of a paper about the stochastic theory of the behaviour of single molecules. It has been cited at an almost constant rate since it was published in 1985 (it was published in a book, so it does not count as a publication—the *Web of Science* cannot even measure citations properly). Fig. 8(b) shows citations of a paper which was cited rarely for 40 years after it was published in 1971 (Hawkes, 1971), but which has recently been discovered, though its author no longer needs promotion.

How do you compare citation patterns like these? You cannot!

One can do no better than to repeat the words of Bruce Alberts (Alberts, 2013):

‘… the leaders of the scientific enterprise must accept full responsibility for thoughtfully analyzing the scientific contributions of other researchers. To do so in a meaningful way requires the actual reading of a small selected set of each researcher's publications, a task that must not be passed by default to journal editors.’

### Conclusions

Varin and his colleagues have done a thorough and thoughtful analyis of the many methods for ranking journals. The results amount to yet another demolition of them.

My conclusion would be that more research in the area cannot be justified. Future efforts should be concentrated on abolishing journal rankings, removing perverse incentives to publish too much and making sure that the declaration on research assessment agreement is implemented.

JIFs are of no interest to anybody but librarians (and not of much interest to them). Publishing is undergoing rapid changes at the moment. Nobody knows where we shall be in 10 years' time. Traditional journals may well wither, to be replaced by open access repositories and post‐publication peer review. That will not only bring to an end the harmful competition between journals; it will also save a large amount of money.

**John A. D. Aston** (*University of Cambridge*)

I firstly congratulate Varin, Cattelan and Firth on a very fine piece of work. This is an excellent example of statisticians doing what they do best: attempting to address issues where data are being used in a way that is not fit for purpose, and not only pointing out the deficiencies but also coming up with informative solutions. The analysis is of a very high quality, with a model that appears to fit the data well, and indeed the assumptions behind the model carefully checked. Possibly the most important aspect of the work is that it allows the assessment of uncertainty in these rankings in bibliometrics, which is something that is often inherently ignored. The work is also highly reproducible, with the authors going to considerable lengths to ensure that this is so (I certainly appreciated the ready access to the data and code).

However, I would like to raise some issues which might warrant further attention. Firstly, the analysis in the paper is restricted to journals in statistics. This would not be an inherent problem if the journals themselves were a closed connected component, but this is very much not so. The network of citations between journals is well known to be a dense graph (for example, the connection density of the top 1000 nodes in the largest connected component in the ‘Journal citation reports’ graph is about 32% (Franceschet, 2012)), and as such considerable information is lost when removing links to journals outside statistics. However, statistics is an outward looking discipline. As shown in Table 2, even a journal which would be considered as an archetypal statistics journal, the *Annals of Statistics*, has less than 50% of its outgoing and incoming citations from statistics journals (a fact that somewhat surprised me). By the standards of Facebook nodes, for example, the citation network is small, so it should be possible to consider considerably larger networks, even with appropriate covariates to control for different citations across different disciplines. Ignoring these outside journals implies certain assumptions on the marginalization within the model, and it is not clear that these assumptions are valid.

**Figure 9:**
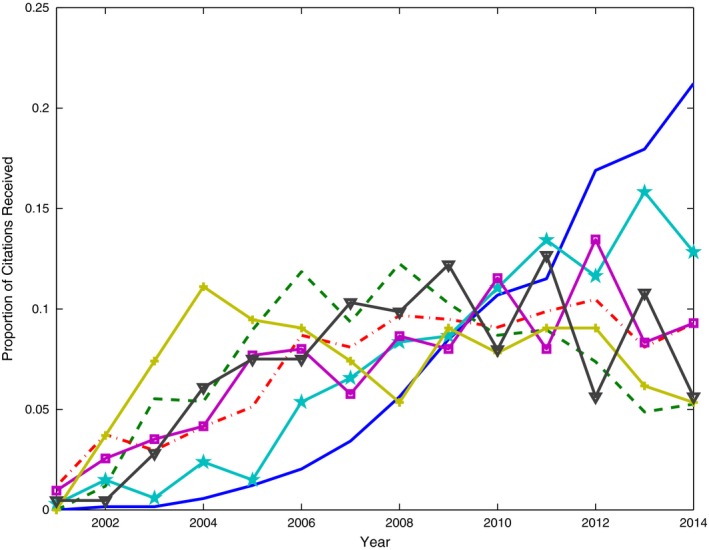
Citation profiles (normalized over time for the top seven papers published in the *Journal of the American Statistical Association* in 2001 (as can be seen, the profile of the top paper is almost exponential, whereas the second paper's influence could be argued to be on the wane, whereas most are slowly growing or flat (after the initial jump) over the time period): 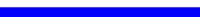, 1; 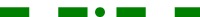, 2; 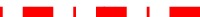, 3; 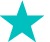, 4; 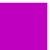, 5; 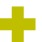, 6; 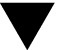, 7

Secondly, the issue of which time window to use to determine the effects would seem to be critical. Varin and his colleagues, quite justifiably, argue that statistics journals need a long time window to see the influence of published work through citations, and hence the 10‐year window was chosen. However, papers are notoriously inhomogeneous even within the same journal, and so a predefined timing window could allow certain effects to be masked. Take the *Journal of the American Statistical Association* for example, and, in particular, the top seven cited papers (so far) published in 2001. As can be seen in Fig. 9, the profiles are remarkably different in some cases (because of the differing applied and methodological nature of the papers), indicating that the choice of a time window will probably affect interpretations within a tournament analysis.

There are, of course, discussions to be had over the methodological choices made. It is not clear that journals really compete to take papers (it often appears to be the contrary!), so the appropriateness of a tournament model for ranking the journals is certainly debatable. However, it is not immediately clear what other type of statistical approach would be more suited to a journal ranking. As to the choice of the model itself, the fact that overdispersion was included in the model was commendable but, given the highly complex microstructure of journal citations (including the variability added because of some of the assumptions discussed above), it is not clear that a one‐parameter model is really adequate (although the diagnostics suggest that it broadly is). The addition of the lasso penalty to the model has both pluses and minuses, in that it creates some nice (if somewhat debatable) groupings, but it also ranks many journals as equal. Academics can debate endlessly the importance of one journal over another and, although there may not be the information to separate them, the use of the lasso can create the illusion of true equality. It would be of interest to consider other penalized techniques (such as ridge regression) and their effects on the analysis.

However, the final comment is more a comment on the field of bibliometrics as a whole. As shown in the paper, used in an average analysis, as in the comparison with the 2008 research assessment exercise, bibliometrics do not do too badly, although there are definitely differences and inconsistencies. However, increasingly, these citation figures are not being used on average, but rather to evaluate and assess individual researchers. Such an oversimplification is, of course, inherently dangerous if weight is given to such metrics in crucial decisions such as grant awards or promotion and tenure cases. The authors have gone to some pains to explain that their models should not be used in this way, but possibly more importantly, by showing the inherent uncertainty in all these rankings, it may help to convince others of the limitations of such bibliometrics.

The vote of thanks was passed by acclamation.

**Francesco Bartolucci** (*University of Perugia*)

I really appreciated the paper for the relevance of the theme dealt with, which is testified by the introduction in several countries of evaluation systems partially based on journal rankings (Bartolucci *et al*., 2015), and also for the use of well‐principled statistical methods.

Regarding possible themes of discussion, I would like to focus on the following four.

- *Model extensions*: I see some connections between the Stigler (1994) model and the Rasch (1961) model, which is routinely applied for the analysis of item response data. It is well known that the Rasch model can be easily generalized to include discrimination coefficients which measure the degree of dependence of each item response on the underlying ability. I was wondering whether a similar generalization is possible here; this would amount to assuming that
  $$\text{log‐odds}\left(\text{journal}\;i\;\text{is cited by journal}\;j\right)=\alpha_{ij}\left(\mu_{i}-\mu_{j}\right),$$
  where each discrimination parameter $\alpha_{ij}$ can be interpreted as a measure of dependence of the result of the ‘match’ between journals *i* and *j* on their export scores. A possible strategy is formulating the $\alpha_{ij}$‐parameters as proportional to a measure of ‘closeness’ between the journals of the same type as used by the authors in the preliminary clustering. In this way, it would be also less important to start from a strictly homogeneous set of journals, as non‐homogeneous comparisons may be corrected for by these parameters.
- *Lasso penalization*: in the recent literature about the lasso and related estimation techniques, there have been recent advances about the choice of the type of penalization when the final aim is clustering. I am referring, in particular, to the truncated lasso and minimax concave penalty functions (Pan *et al*., 2013; Marchetti and Zhou, 2014), which have advantages in terms of clustering, as clusters are formed without a shrinkage towards 0 of their centroids.
- *Fractioned citations*: I really appreciated the suggestion of weighting citations on the basis of the length of the list of references in the citing paper. I was also wondering whether it is possible to weight for the number of authors of the cited paper, as there is a tendency in certain fields to overcite papers with many authors (Batista *et al*., 2006).
- *Self‐citations*: the number of self‐citations is ignored at journal level although the authors seem not to consider self‐citations as always unfair. Then, I was wondering whether self‐citations can be somehow accounted for in the model so that they affect the $\mu_{i}$‐parameter estimates. I admit that this point is controversial as editorial boards of certain journals put pressure on authors to cite papers that have already been published in the same journal.

Overall, I thank the authors for their contribution and I hope that these points can represent suggestions for further developments of the approach proposed.

**Jason Wyse and Arthur White** (*Trinity College Dublin*)

We congratulate Varin, Cattelan and Firth for robustly discussing the many issues associated with ranking journals, issues which are in danger of being ignored, in this timely and important paper. As early career statisticians ourselves, we wonder whether the use of impact factors tells us to aspire to a career of quantity over quality.

Our comments focus on the possibility of clustering journals by using a hybrid of the Stigler model and stochastic block model (Nowicki and Snijders, 2001) seen in network analysis. This model makes it possible to organize journals explicitly into groups having equivalent levels of influence. In our application these groups can reasonably be ranked in order of importance. This procedure bears some relation to that of grouping rankings by the lasso. Attaching a latent group variable *z* to each journal we can assume that

$$\text{Pr}\left(\text{journal}\;j\;\text{cites journal}\;i|z_{i}=k,z_{j}=l\right)=\theta_{l\to k}$$

where *k*,*l* ∈ {1,…,*K*}. We can think of the parameter $\theta_{l\to k}$ as in some ways analogous to the export score of the Stigler model so, the closer the value to 1, the greater the propensity to export intellectual influence over journals belonging to group *l*. In this hybrid model the probability that journals *i* and *j* exchange citations depends on the latent variables $z_{i}$ and $z_{j}$ and as such is affected by the identity of the citing journal.

The probability that a journal *i* belongs to group *k* is $w_{k}$, with $\Sigma_{k}w_{k}=1$. The distribution of $C_{ij}$ conditional on the group labels $z_{i}$ and $z_{j}$ is binomial($t_{i_{j}},\theta_{z_{j}\to z_{i}}$). Several constraints are inherited from the Stigler model; in particular we must have $\theta_{k\to k}=0.5$ for *k*=1,…,*K*. In this paper journals with very similar export score after the grouped lasso analysis have $\mu_{i}-\mu_{j}\approx 0$ and are therefore roughly equally likely to cite each other.

Assuming standard priors on the hybrid model parameters we fitted the model using *K*=7 groups by using a Gibbs sampler. The *Journal of the Royal Statistical Society*, Series B, was placed in a singleton group 1 with the *Annals of Statistics*,* Biometrika* and the *Journal of the American Statistical Association* in group 2.

**Figure 10:**
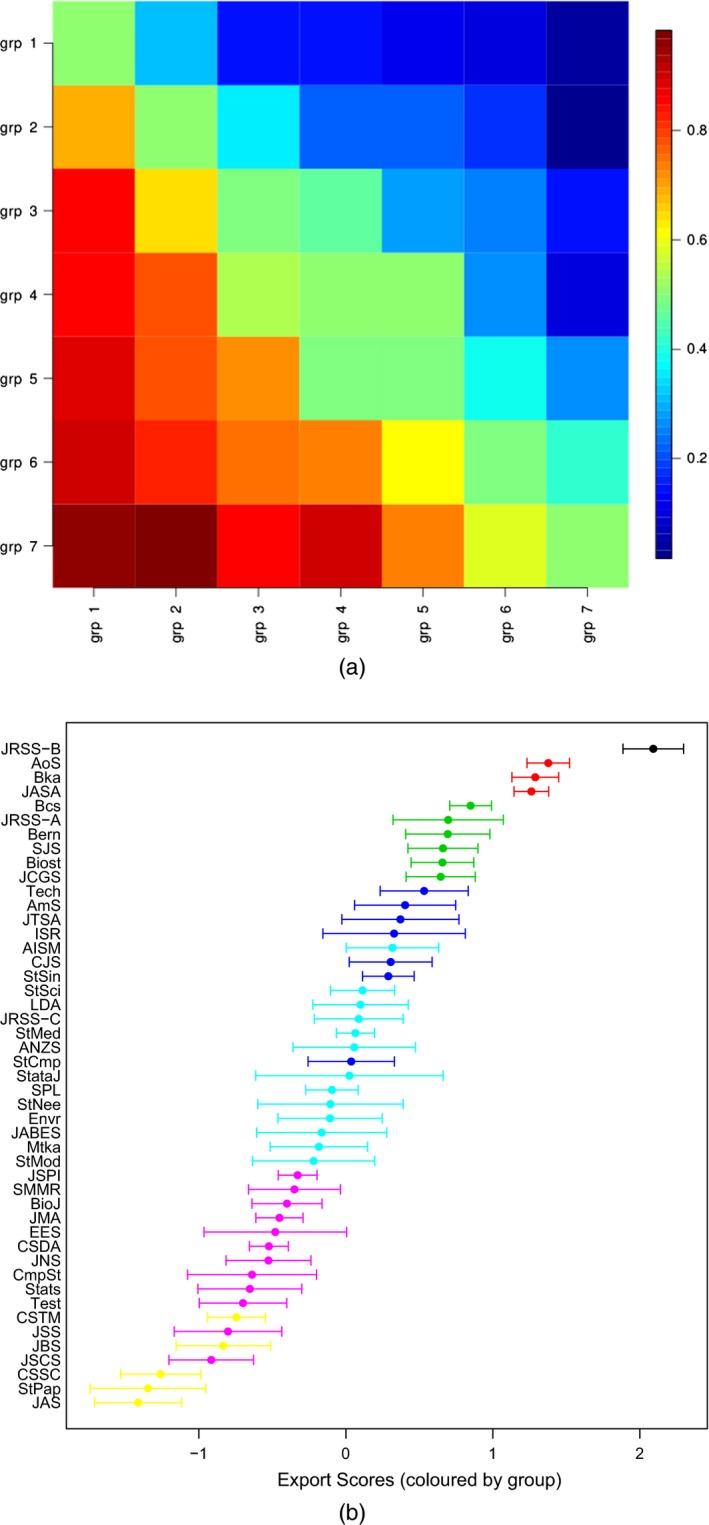
(a) Heat map of posterior means of $\theta_{l\to k}$ and $\theta_{k\to l}=1-\theta_{l\to k},l<k$, and (b) Fig. 4 showing the groupings found from the hybrid model

Although we do not obtain journal‐specific export scores from the hybrid model, we can draw parallels with Table 5. Fig. 10(a) shows the posterior means of the $\theta_{l\to k},\;l<k$. Note that $\theta_{k\to l}=1-\theta_{l\to k}$. The first column shows the probability that the *Journal of the Royal Statistical Society*, Series B, is cited by journals in the other groups, rather than vice versa. The probabilities for this group are much closer to 1 (the bottom left portion of Fig. 10(a)) than 0 (the top right portion of Fig. 10(a)). Moving from left to right along columns, there is a clear decline in the probabilities, suggesting a natural ranking of the groups. Fig. 10(b) reproduces Fig. 4 of the paper with colour coding for the groups found from the hybrid model (detailed in Table 7). It is intersting to note how closely the rankings correspond to the groupings.

**Table 7:** Comparison of inferred groupings from the hybrid model and ranks derived from a quasi‐Stigler model

*Rank*	*Group*	*Journal*	*Rank*	*Group*	*Journal*
1	1	JRSS‐B	25	5	SPL
2	2	AoS	26	5	StNee
3	2	Bka	27	5	Envr
4	2	JASA	28	5	JABES
5	3	Bcs	29	5	Mtka
6	3	JRSS‐A	30	5	StMod
7	3	Bern	31	6	JSPI
8	3	SJS	32	6	SMMR
9	3	Biost	33	6	BioJ
10	3	JCGS	34	6	JMA
11	4	Tech	35	6	EES
12	4	AmS	36	6	CSDA
13	4	JTSA	37	6	JNS
14	4	ISR	38	6	CmpSt
15	5	AISM	39	6	Stats
16	4	CJS	40	6	Test
17	4	StSin	41	7	CSTM
18	5	StSci	42	6	JSS
19	5	LDA	43	7	JBS
20	5	JRSS‐C	44	6	JSCS
21	5	StMed	45	7	CSSC
22	5	ANZS	46	7	StPap
23	4	StCmp	47	7	JAS
24	5	StataJ			

**Fionn Murtagh** (*University of Derby, and Goldsmiths University of London*)

The importance of statistical modelling and analytics of citations covers contemporary scholarly publishing, and other domains. Among these other domains are

- the work of research funding agencies,
- evaluation processes leading to resource allocation, at institutional level and at national level,
- journal and conference editorial processes and
- personal promotion and related career procedures.

Citation data, and derived journal rankings, are used to evaluate the scientific influence of researchers. As noted, however,

‘the short time period that is used for computation of the impact factor, … can be completely inappropriate for some fields, in particular for mathematics and statistics’.

The authors question cross‐field comparisons. This is on the grounds of different distributional characteristics for the data. One point made about the mean being a completely invalid summary statistic for long‐tailed distributions needs to be known and understood by everyone involved in decision making and policy making, given the implications for resource allocation.

Contemporary citation counting leads to ‘popularity’ being to the fore, which may or may not be related to deep and consequential influence. Influence can and should come from all of the content, with full account taken of the semantics, and context.

In regard to trends and tendencies using citations, Mustafee *et al*. (2013) used co‐citations (i.e. articles cited by the one citing article) to study turning point articles and authors. Other work (by A. Casey, S. Ahmadi and me) is seeking to evaluate citations, contextually and semantically, in terms of positive and negative roles, and also playing no role.

In Murtagh and Kurz (2016), we analysed over 93000 bibliographic records and found a clear transition from the years 1994–2003 where disciplines that dominated in terms of publications in the area of clustering and classification included mathematics, psychology and biology (especially numerical taxonomy). In the years 2004–2012, the dominant disciplines were management, medicine and engineering among other fields.

Citation analytics through statistical modelling play an important role in the general context of mapping narratives of scholarly disciplines. Ultimately, we need to take full account of content and of semantics. Our aims can include the determining of, and the tracking of, turning points, consolidation of scholarship and learning *versus* new directions to be followed and cross‐disciplinary movements. This is all for application in contexts that encompass social, economic impact, education and training. Ultimate applications for this work are journal editorial processes, funding agency processes, socio‐economic impact assessment, scholarship and learning.

The following contributions were received in writing after the meeting.

**Alan Agresti** (*University of Florida, Gainesville*)

I congratulate Varin, Cattelan and Firth on an interesting and potentially very important paper. With all the current interest in ranking journals, institutions and departments, the impact of this paper could and should be substantial.

My comments are minor and concern a few technical questions. Are the standard errors and quasi‐variances for $\left\{{\hat{\mu }}_{i}\right\}$ robust to the inflated variance quasi‐likelihood approach? For instance, would similar results occur with a beta–binomial‐type variance that results from equally correlated Bernoulli trials? Perhaps so, but the two variance structures are quite different when the $\left\{t_{ij}\right\}$ vary greatly, and the simple inflated variance approach breaks down when $t_{ij}=1$, though this is not a concern here. For any variance structure, perhaps journal residuals would be closer to standard normal distributions (under the model) if they employed standardized residuals instead of Pearson residuals. For logistic and log‐linear modelling, Pearson residuals can appear overly optimistic because of not adjusting for the fitted values themselves being estimates.

The lasso seems a very sensible way to discourage overly fine interpretation of league table results. For a particular shrinkage choice *s* for the lasso or adaptive lasso, how can one construct an analogue of the centipede plot in Fig. 4? Presumably a bootstrap would yield a non‐symmetric appearance of intervals around the lasso estimates, as seems natural with lasso estimates of 0.

**Julyan Arbel** (*Collegio Carlo Alberto, Moncalieri*) **and Christian P. Robert** (*University of Warwick, Coventry, and Université Paris‐Dauphine*)

Although we commend the authors on a scholarly and statistical approach to the issue of citations and impact factors, we remain sceptical of such modelling in that it facilitates the bibliometric short cuts in the analysis of researchers’ record and impact.

**Figure 11:**
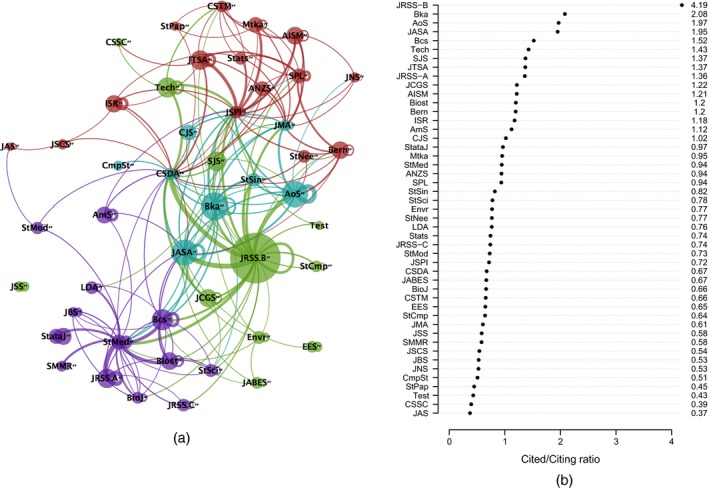
(a) Graph of the matrix ${\tilde{C}}_{ij}$ of citations normalized by the total of citations made by each journal, to account as a proxy for the size (number of papers published) of the journal (the node size represents the (total of citations received)/(total of citations made) ratio; the edge width represents ${\tilde{C}}_{ij}$ (for visibility, only the first deciles of heaviest edges are represented); the four clusters identified are modularity classes obtained by the Louvain method) and (b) ratio (total of citations received)/(total of citations made for each journal) (note the similarity to Fig. 4, which illustrates export scores from Stigler's model)

The first feature of interest in the analysis of the data is that all the 47 journals have a majority of citations from and to journals outside statistics or at least outside the list. This property is not exploited further, whereas we find it remarkable: the most influential statistics papers should be those that result in new methodologies adopted by all fields, but the restriction of the modelling to a closed universe of other statistical journals misses this dimension. A lesser feature is that both the *Journal of Computational and Graphical Statistics* and *Statistics and Computing* escape the computational cluster to end up in theory and methods along with the *Annals of Statistics* and Series B, which may signal that papers published in those journals are more focused on the theory of computational statistics than on developing computing products for a wider audience. The paper does not report a graph of the data, even though it is an informative piece of information here; see Fig. 11(a).

In addition to the unsavoury impact factor, a ranking method discussed therein is the eigenfactor score that starts with a Markov exploration of articles by going at random to one of the papers in the reference list. Althought this modelling is both mathematically and behaviourally compelling, it shares a drawback with the impact factor, namely that it does not account for the reason the paper is cited. Furthermore, it necessarily induces a bias towards more theoretical or methodological journals as application and implementation papers are unlikely to be quoted.

The major modelling input is based on Stigler's model. The ‘big four’ once again receive high scores, with Series B still far ahead. Note that the ratio (total citations received)/(total citations made) for each journal (Fig. 11(b)) mimics quite closely the scores of Stigler's model in Fig. 4. The authors later question the bias due to the ‘read paper effect’, but they cannot easily evaluate it. Although some such papers like Spiegelhalter *et al*. (2002) generate enormous citations, other journals like *Statistical Science* also favour discussion papers. The causality arrow of such successful papers is difficult to build as it may be argued that the popularity of the method led it to be discussed: this is clearly so for Spiegelhalter *et al*. (2002), with their deviance information criterion being much in use by the time that the paper was submitted.

We appreciate the perspective in the conclusion, namely that clusters of journals or researchers have very close indicators, which means that ranking and analyses should be conducted with more caution, and that reverting indices from journals to researchers and individual papers has no validation and provides little information.

**Mathieu Bray and Peter X. K. Song** (*University of Michigan, Ann Arbor*)

Varin, Cattelan and Firth should be congratulated for their interesting paper providing an alternative way to evaluate statistical journals through their ability to export intellectual influence.

Rigorous inclusion–exclusion criteria should be discussed for the selection of comparable journals. In particular, since the selection contains both theoretical and applied journals, we wonder how journal homogeneity is ensured. Is the purpose of the clustering analysis to provide an examination of journal homogeneity? Can the singleton journals be removed (the analysis seems to suggest that they are different from the remaining journals)?

The use of papers published only in a single year may lead to year‐specific results. Sensitivity analysis on the influence of single *versus* multiple years would be appealing. Sensitivity analysis on the number of years to look back may also be worthwhile (e.g. the previous 5 years, comparable with the article influence score).

A possible issue of the pairwise Pearson correlation approach (seen in Fig. 2) is that marginal correlation may be affected by the number of strongly connected journals. We may regard these 47 journals as a network, in which partial correlation is more appropriate to characterize a ‘genuine’ dependence between two journals, conditioned on all others (Zhao *et al*., 2011).

One shortcoming is the ignorance of confounding factors (e.g. journal age and Web accessibility). The top journals are generally the oldest in the statistical sciences (seen in Fig. 4), and emerging journals do not seem to be fairly evaluated.

Citation counts $C_{ij}$ are not independent; the use of diagonal covariance in the quasi‐likelihood estimation equation (6) did not account for such dependence, resulting in potentially wider confidence intervals in Fig. 4. This may also affect results in Table 5.

The main conclusion from Section 5.5 that many estimated scores are not distinguishable from one another is an argument for ranking by levels instead of on a continuous scale. Since the export scores are naturally ordered (seen in Fig. 4), a simpler penalty may be set up by considering only differences between adjacent pairs. As shown in Ke *et al*. (2015) and Wang (2012), this penalty, based on consistently estimated ordering, can achieve lower estimation error and faster, more stable numerical performance.

The use of homogeneous journals loses the opportunity to assess the broader influence of statistical journals. With the integration of statistics into interdisciplinary studies accelerating, it is important to understand how publications in statistical journals impact research in subject matter sciences.

**Jane Carlen and Mark S. Handcock** (*University of California, Los Angeles*)

Citation data can be fruitfully thought of from a networks perspective. Visualizing the network of journals via graphical layout algorithms offers a summary of relationships, clustering and centrality but is coupled with artefact.

The Stigler model estimates ‘export scores’ $u_{i}$ such that $c_{ij}$ is assumed binomially distributed with $E\left(c_{ij}\right)=t_{ij}\text{exp}\left(\alpha_{i}+\beta_{j}\right)$ and $u_{i}=\alpha_{i}-\beta_{i}$, as in ‘quasi‐symmetry’ formulation (4). We can place these assumptions in the context of an exponential family random graph model on a valued network, where edge weights are directed citation counts (Krivitsky, 2012). A direct extension of the Stigler model would retain the assumption of binomially distributed citations. Here we consider a Poisson model with canonical link and mean modelled with sender and receiver effects. The corresponding estimates of export scores (i.e. journal‐specific receiver minus sender coefficient) are highly correlated (0.95) with those of the Stigler model reported in Table 5.

A benefit of the network model is extensibility, both theoretically and computationally. To illustrate, consider the two‐dimensional latent space model with sender and receiver effects (Krivitsky *et al*., 2009). This model posits distances between journals as latent variables that effect edge weights (citation counts) (see Hoff *et al*. (2002) and Krivitsky and Handcock (2008, 2015)). The corresponding estimates of export scores are very highly correlated (0.99) with those of the Stigler model.

**Figure 12:**
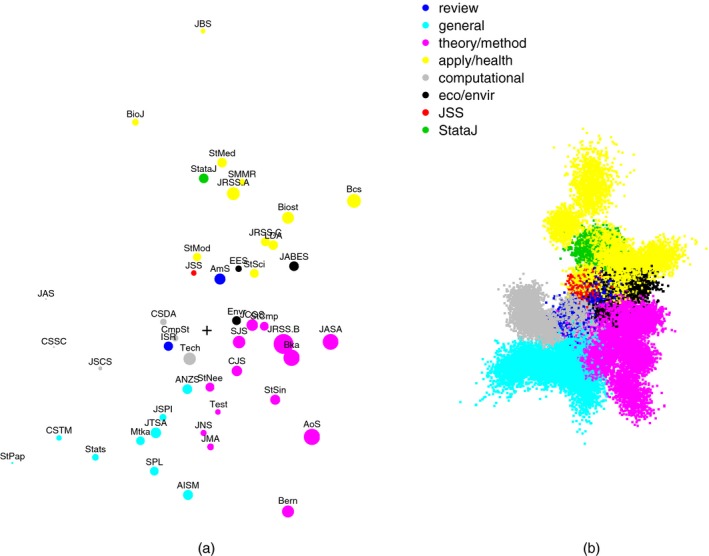
Estimated journal positions from the two‐dimensional latent space model: (a) point estimates with node size scaled to the receiver minus sender coefficient; (b) sample of positions from the model (the shades are due to the hierarchical clustering of the authors (Section 3))

Model‐based estimates of journal positions are shown in Fig. 12. Although there is no clustering term in the latent space model, the clustering presented in the paper (Section 3) is fairly well captured. The plots illustrate how individual journals and clusters fit together. However, we should be careful not to reify these point estimates of positions. Fig. 12(b) displays the uncertainty in the positions by using a sample of draws from the model.

Fig. 12 offers a visual aid to the observation (Section 7.2) that many journals are not significantly different in rank, and therefore grouped rankings are often more appropriate than traditional ordering. We see a periphery of low ranked journals on the left and a small cluster of leading journals around the *Journal of the Royal Statistical Society*, Series B, but beyond that a widely dispersed middle. Centrality does not equate to rank or prestige, as shown by the *Annals of Statistics* and *Bernoulli* in the bottom right.

The models are readily fitted with the latentnet package (Krivitsky and Handcock, 2015) and the code is available from http://www.stat.ucla.edu/~jane.carlen/.

**Miguel de Carvalho** (*Pontificia Universidad Católica de Chile, Santiago*)

I congratulate Varin, Cattelan and Firth for this magnificent paper. Scientific reputation is perhaps the most valuable asset a scholarly journal can hold. Reputation has a temporal aspect, but the current analysis—although extremely enlightening and thought provoking—only provides a snapshot of the ‘prestige’ of statistics journals. The authors acknowledge this in Section 7.4.2, where they discuss the insights that a dynamic Bradley–Terry model could offer. A dynamic analysis would pose new challenges, such as the reliability of realtime estimates of export scores. Suppose that we estimate ${\left\{\mu_{i}^{2015}\left(t\right)\right\}}_{i=1}^{n}$. using data until 2015, and that on 2016 we estimate ${\left\{\mu_{i}^{2016}\left(t\right)\right\}}_{i=1}^{n}$. Ideally, the estimate ${\hat{\mu }}_{i}^{2016}\left(2015\right)$ should not differ too much from ${\hat{\mu }}_{i}^{2015}\left(2015\right)$—otherwise the estimation method ‘regrets’ too much the estimate that it produced earlier—but different estimation methods should have different *revision* properties. Some revision is acceptable and desirable, but it seems difficult to trust an inference method that revises substantially its estimates for previous years.

**Figure 13:**
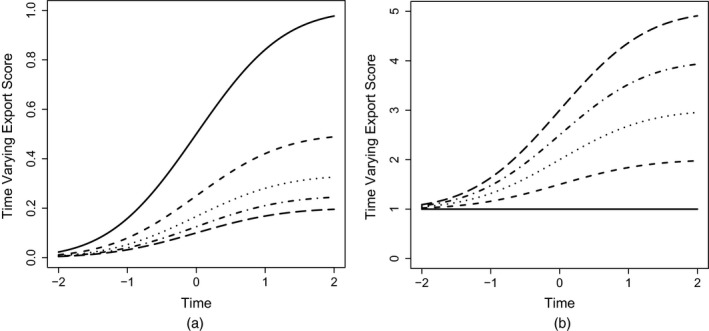
(a) $\mu_{i}\left(t\right)=1/\left\{i\Phi \left(t\right)\right\}$, so that the long‐run export scores are ${\overset{¯}{\mu }}_{i}=1/i$, for *i*=1,…,5, and (b) $\mu_{i}\left(t\right)=1+\left(i-1\right)\Phi \left(t\right)$, so that the long‐run export scores are ${\overset{¯}{\mu }}_{i}=i$, for *i*=1,…,5

If we had a sufficiently long span of data, the question of extrapolating—out of the observation period—into the long run could arise. But, for this, it would be desirable that $\mu_{i}\left(t\right)$ and ${\hat{\mu }}_{i}\left(t\right)$ had finite limits when *t*→∞, so that we could compute long‐run export scores ${\overset{¯}{\mu }}_{i}:={\text{lim}}_{t\to \infty }\mu_{i}\left(t\right)$, and ${\overset{¯}{\pi }}_{ij}:=\text{exp}\left({\overset{¯}{\mu }}_{i}-{\overset{¯}{\mu }}_{j}\right)/\left\{1+\text{exp}\left({\overset{¯}{\mu }}_{i}-{\overset{¯}{\mu }}_{j}\right)\right\}$. Interpretation of these quantities would warrant care, but could it provide some insights? For instance if the true time varying export scores are $\mu_{i}\left(t\right)={\underline{\mu }}_{i}+\left({\overset{¯}{\mu }}_{i}-{\underline{\mu }}_{i}\right)\Phi \left(t\right)$, with ${\underline{\mu }}_{i}\leq {\overset{¯}{\mu }}_{i}$, then ${\overset{¯}{\mu }}_{i}$ would represent the corresponding long‐run export scores. See Fig. 13 for examples.

Related to Section 7.4.2 is also the possibility of defining predictor‐dependent export scores $\mu_{i}\left(x_{i}\right)$ extending naturally the set‐up discussed in the paper. This could be done with the structured model $\text{logit}\left\{\pi_{i,\;j}\left(x_{i},x_{j}\right)\right\}=\mu_{i}\left(x_{i}\right)-\mu_{j}\left(x_{j}\right)$. For example, one could be interested in such a covariate‐adjusted version of the export score to assess how it could evolve over covariates such as a society‐sponsored journal (dummy) and the number of associate editors; a related proposal is discussed in Firth (2009), section 2.

The current comparison does not take into account econometrics journals. Although the argument of ‘retaining those [journals], which communicate more’ is compelling, and well justified by the authors, it raises the question ‘Do we want each community to be ranked separately, or for subject‐related topics to be ranked together?’. *Econometrica* is definitely special in this respect, because it is a prominent wide scope journal in economics, and nowadays it certainly publishes more on game theory than on statistics and econometrics: but what about the *Journal of Business and Economic Statistics* or, say, the *International Journal of Forecasting*? I definitely think that these—and other theory and methods journals in psychometrics and machine learning—are still in the ‘domain of attraction’ of our profession.

**Daniela Cocchi** (*Università degli Studi di Bologna*)

Statisticians need to be more influential in scientific journals ranking procedures. To pursue that, treatment of uncertainty should be a primary topic, even if dealing with differences and variability is equally important. The work of this nice paper, although addressing all these topics, induces some thoughts, more related to the role and perception of statistical methods, rather than to specific technical points. The paper points out, however, that the results that are obtained under a strictly restrictive data collection match those obtained with less formal methods applied to larger data sets. The connection found makes this approach quite appealing for its robustness, witnessed by the similarity of its results with results obtained in different contexts.

The paper starts its analysis from an initial well‐defined set, which is the basis for any further analysis. This starting point is restrictive and intended to exclude rather than to include. This is a somewhat typical approach in statistics, where, before focusing on the theoretical developments, particular care is dedicated to state precise definitions to isolate the reference data set. The motivation to consider a relatively small set of clearly ‘statistical’ journals is acceptable, since the focus of the paper is citation exchange. However, in my experience, the definition of a statistical journal in its broadest sense is difficult. For example, in my department, to obtain a consensus on the set of scientific journals that could be a reference point, we have finally settled for the union of different lists, some published by the Ministry of Universities and others by scientific Societies, rather than for their intersection.

Moreover, since many are the journals where statisticians have the opportunity to publish, our choice has a huge spectrum, covering a very large number of scientific categories (in both the *Web of Science* and Scopus). We had to deal with two different problems. The first was the remarkable differences in the indicators along categories, whereas the second was the fact that a journal, and its specific set of indicators, can enter more than a single category and is differently ranked depending on each of them. As a first step, we proposed simple descriptive normalizing and averaging procedures (Cocchi *et al*., 2014).

I would appreciate the opinion of the authors on

- the duality between a restricted rather than extended initial data set,
- their ideas on normalization and
- their suggestions concerning non‐trivial but understandable (for non‐statisticians) ways of averaging results obtained separately in each category.

**Peter Darroch** (*Elsevier BV, Amsterdam*)

This excellent paper presents interesting and thought‐provoking analyses and raises many important points including

- the care which should be exercised when using journal metrics, especially when evaluating the scientific influence of individuals,
- the difficulty in defining fields of research and so, in this case, clusters of journals, for meaningful analysis and
- the limited involvement of statisticians in the field of bibliometrics.

I believe that the paper should stimulate welcome discussion and I commend the authors on the transparency and completeness of the analysis presented. I have some brief comments to share. The first is regarding the creation of a homogeneous journal set and what this means for any analysis. Many new journals are launched each year and there is a large amount of interconnectedness across journals. Research is also becoming more heterogeneous and interdisciplinary, not least in the field of statistics as noted by the authors. Therefore, a model that is scalable and can encompass the whole network of journals is ideal.

Secondly, given the need for a scalable and encompassing approach, any selection of a time window or exclusion of journals should be done with care, related to the specific purpose and also highlighted. Indeed, analysis shows that, for a broad database, a 3‐year window is the most appropriate (Lancho‐Barrantes *et al*., 2010), which is reflected in the implementation of journal metrics within the tools that Elsevier supply.

Thirdly, related to the comparison with the UK research assessment exercise scores, I would like to reiterate the profound implications that an inappropriate use of any type of measurement can have on individuals, especially the inappropriate use of journal rankings to judge individuals. The use of metrics is currently under scrutiny in the UK through the Higher Education Funding Council for England's review of the role of metrics in research assessment (http://www.hefce.ac.uk/rsrch/metrics/). As highlighted in Elsevier's response to this review (http://bit.ly/hefceresponse) and Hicks *et al*. (2015) (http://www.nature.com/news/bibliometrics-the-leiden-manifesto-for-research-metrics-1.17351), there are some basic guidelines which should be employed, along with a good dose of common sense, to ensure that metrics and other types of measurement provide valuable, beneficial and appropriate insight. Two basic principles, to be applied without exception, are to include input from multiple types of measurement be it peer review, expert opinion or relevant metrics, and that when using metrics more than one relevant metric is used.

**Karin S. Dorman and Ranjan Maitra** (*Iowa State University, Ames*)

We congratulate the authors on a stimulating and thought‐provoking paper. Appealingly, the Bradley–Terry (BT) model treats journal pairs (*i*,*j*), conditional on $T_{ij}$ (using the authors’ notation), independently, but limitations exist. The BT model ignores variability in $T_{ij}$s and assumes quasi‐symmetry (Agresti, 2013), which, when violated, can yield incorrect rankings. We find the Poisson model (PM) a flexible and potentially more natural representation of the generative process for citation counts $C_{ij}$.

Let $C_{ij}$ be Poisson distributed with log‐linear mean $\alpha_{i}+\beta_{j}+\gamma_{ij},\;i\neq j$, where $\alpha_{i}$ reflects the ability of journal *i* to attract citations, $\beta_{j}$ the citation output of journal *j* and $\gamma_{ij}$ any boost or depression in citations of journal *i* by *j*. Under quasi‐symmetry, $\gamma_{ij}=\gamma_{ji}$ and both models yield similar estimates of scaled export scores ($\mu_{i}$ for the BT model or $\alpha_{i}-\beta_{i}$ for the PM). Although $\alpha_{i}$ and $\beta_{j}$ become inestimable in the full asymmetric model, assuming sparse asymmetry using a PM with interactions improves recovery of journal rankings in simulation experiments (Table 8). The estimated interactions may be intrinsically interesting, indicating collusion between journals (van Noorden, 2013) or self‐citation activity when $C_{ii}$s are also modelled. Further, the PM estimates both $\alpha_{i}$ and $\beta_{j}$, making it possible to scale export scores by something beyond import activity, as forced by the BT model: for example, $\alpha_{i}$ ranks journal by impact regardless of size. After all, a large egalitarian journal can achieve as much total scientific impact as a tiny elite journal.

**Table 8:** Journal interactions and rank estimation†

*μ*	*Results for n*=500				*Results for n*=5000				*Results for n*=15080			
	*BT*	*PM*	$PM_{\gamma }$	$PM_{\hat{\gamma }}$	*BT*	*PM*	$PM_{\gamma }$	$PM_{\hat{\gamma }}$	*BT*	*PM*	$PM_{\gamma }$	$PM_{\hat{\gamma }}$
*Asymmetric interactions*												
0	0.84	0.88	0.88	0.88	0.98	0.98	0.98	0.98	0.99	0.99	0.99	0.99
1	0.84	0.88	0.88	0.88	0.97	0.98	0.98	0.98	0.99	0.99	0.99	0.99
2	0.81	0.86	0.86	0.86	0.96	0.97	0.98	0.98	0.97	0.97	0.99	0.99
3	0.79	0.82	0.86	0.84	0.93	0.92	0.98	0.98	0.94	0.93	0.99	0.99
*Symmetric interactions*												
0	0.84	0.88	0.88	0.88	0.98	0.98	0.98	0.98	0.99	0.99	0.99	0.99
1	0.83	0.88	0.88	0.87	0.98	0.98	0.98	0.98	0.99	0.99	0.99	0.99
2	0.82	0.86	0.87	0.86	0.98	0.98	0.98	0.98	0.99	0.98	0.99	0.99
3	0.83	0.86	0.87	0.85	0.98	0.96	0.98	0.98	0.99	0.97	0.99	0.99

†Average Spearman correlations between the true and estimated ranks for 100 replicates of Poisson‐simulated data with log‐linear mean $\alpha_{i}+\beta_{j}+\gamma_{ij}$. Effects were simulated as independent standard normal for $\alpha_{i}$ and $\beta_{j}$, and *N*(*μ*,1) with specified mean *μ* for 20 randomly selected (*i,j*) under the asymmetric model and 10 randomly selected (*i, j*) with $\gamma_{ij}=\gamma_{ji}$ under the symmetric model. All other interactions were 0. The methods of estimation are the BT model, the PM without interactions, the PM estimating the true non‐zero interactions, ${\text{PM}}_{\gamma }$, and the PM estimating self‐selected interactions, ${\text{PM}}_{\hat{\gamma }}$. For ${\text{PM}}_{\hat{\gamma }}$, we recursively added the interaction for the pair of journals with highest Studentized residual as long as the Bayesian information criterion improved substantially (Kass and Raftery, 1927). Here, but not for the real data, we limited the number of interactions to 20. The total mean number of citations, $\Sigma_{i\neq j}\alpha_{i}+\beta_{j}+\gamma_{ij}$, across all journal pairs is specified as *n*.

**Figure 14:**
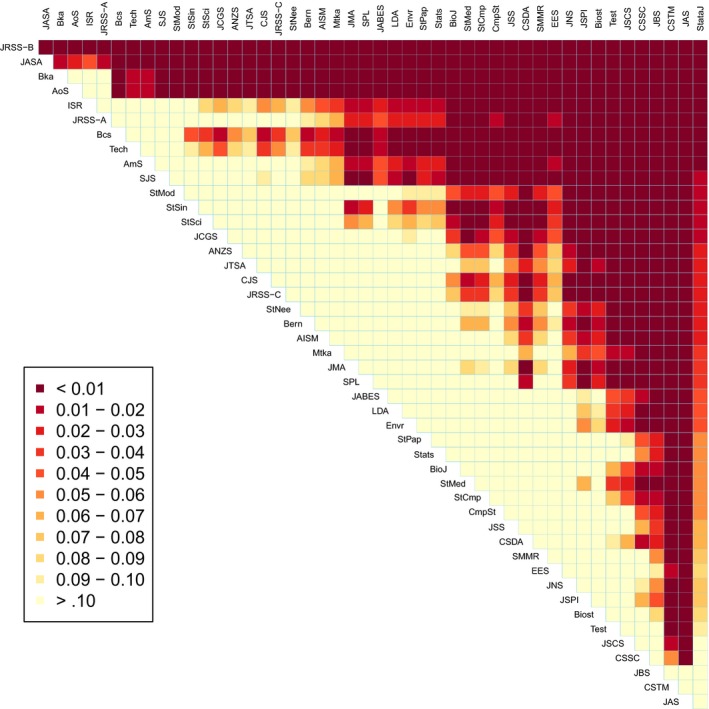
False discovery rates (*q*‐values) (Benjamini and Hochberg, 1995) obtained from testing whether the journal in the *i*th row is significantly better at exporting journal citations than the journal in the *j*th column: the journals are ordered in the sequence of the highest to lowest estimated scaled citation export scores by using the PM with interactions (Table 9) (at a threshold of 5%, both JRSS‐B and JASA are each significantly higher than their competitors; JRSS‐B has a significantly higher citation export score than JASA; among Bka, AoS, ISR or JRSS‐A, there is no significant difference, although Bka and AoS are significantly better than all journals ranked lower than JRSS‐A; ISR and JRSS‐A also join another, overlapping, group (with Bcs, Tech, AmS, SJS and StMod) of mutually indistinguishable journals, in terms of ranking; there are several other such overlapping groups among the rest)

**Figure 15:**
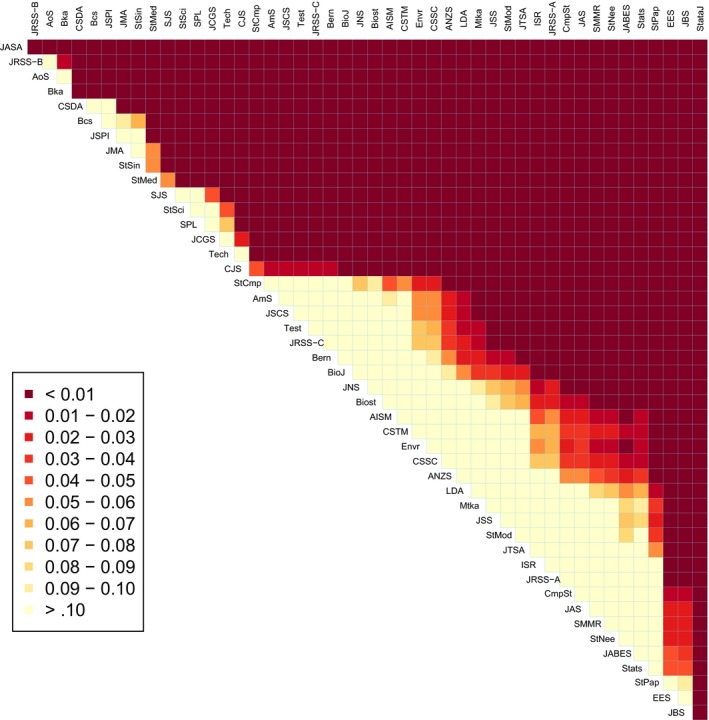
Significance map of the rankings based on unscaled export scores ${\hat{\alpha }}_{i}$ (by using methods similar to those used in Fig. 14): JASA is significantly better at exporting citations than anyother statistics journal; JRSS‐B is also significantly better than all remaining journals except AoS

Turning to the 2010 ‘Journal citation reports’ data set, we implement a simple approach for adding interactions to the PM (see the footnote to Table 8) and compare rankings based on ${\hat{\alpha }}_{i}-{\hat{\beta }}_{i}$ with those of the authors (Table 9). Fig. 14 displays significance of these ordered, pairwise rankings. The rankings largely agree, with a few striking differences. For example, Biost drops out of the top 10 into the lower third. Biost receives high numbers of citations from StMed, Bcs and 10 other journals but, apparently, it does not universally attract citations. Thus, on considering interactions, ${\hat{\alpha }}_{\mathrm{Biost}}$ and, consequently, its ranking drop substantially. In contrast, StMod cites StMed, CSDA and three other journals often, so ${\hat{\beta }}_{\mathrm{StMod}}$ is lowered, which increases the ranking of ${\hat{\alpha }}_{\mathrm{StMod}}-{\hat{\beta }}_{\mathrm{StMod}}$ in the PM with interactions. Table 10 and Fig. 15 show corresponding results for ranking by ${\hat{\alpha }}_{i}$, the unscaled measure of export ability.

**Table 9:** Journal rankings by Poisson‐estimated export score ${\hat{\mu }}_{i}={\hat{\alpha }}_{i}-{\hat{\beta }}_{i}$†

*PMR*	* BTR*	*IFR*	*Journal*	*BT*	*PM*	*PMR*	*BTR*	*IFR*	*Journal*	*BT*	*PM*
1	1	1	JRSS‐B	2.09	1.95	25	28	30	JABES	−0.16	−0.04
2	4	8	JASA	1.26	1.39	26	19	27	LDA	0.10	−0.06
3	3	11	Bka	1.29	1.09	27	27	23	Envr	−0.11	−0.07
4	2	2	AoS	1.38	1.08	28	46	37	StPap	−1.35	−0.16
5	14	28	ISR	0.33	0.82	29	39	39	Stats	−0.65	−0.19
6	6	5	JRSS‐A	0.70	0.70	30	33	16	BioJ	−0.40	−0.19
7	5	13	Bcs	0.85	0.68	31	21	7	StMed	0.06	−0.21
8	11	15	Tech	0.53	0.66	32	23	10	StCmp	0.04	−0.24
9	12	24	AmS	0.40	0.58	33	38	40	CmpSt	−0.64	−0.24
10	8	29	SJS	0.66	0.52	34	42	4	JSS	−0.80	−0.33
11	30	31	StMod	−0.22	0.39	35	36	18	CSDA	−0.52	−0.34
12	17	26	StSin	0.29	0.37	36	32	12	SMMR	−0.35	−0.38
13	18	6	StSci	0.11	0.32	37	35	14	EES	−0.48	−0.40
14	10	17	JCGS	0.64	0.27	38	37	42	JNS	−0.53	−0.42
15	22	36	ANZS	0.06	0.25	39	31	32	JSPI	−0.33	−0.45
16	13	34	JTSA	0.37	0.23	40	9	3	Biost	0.66	−0.50
17	16	33	CJS	0.30	0.23	41	40	20	Test	−0.70	−0.59
18	20	35	JRSS‐C	0.09	0.23	42	44	41	JSCS	−0.92	−0.65
19	26	46	StNee	−0.10	0.21	43	45	45	CSSC	−1.26	−0.80
20	7	22	Bern	0.69	0.17	44	43	19	JBS	−0.83	−1.08
21	15	25	AISM	0.32	0.14	45	41	44	CSTM	−0.74	−1.30
22	29	38	Mtka	−0.18	0.07	46	47	47	JAS	−1.41	−1.61
23	34	21	JMA	−0.45	0.03	47	24	9	StataJ	0.02	−2.13
24	25	43	SPL	−0.09	0.01						

†Rankings as estimated by using Poisson regression with interactions as described in the text. PMR is the Poisson model ranking, BTR is the BT ranking, IFR is the impact factor ranking, BT is the BT export score and PM is the Poisson model export score.

**Table 10:** Journal rankings by unscaled export score ${\hat{\alpha }}_{i}$, where the ability to influence other journals is not scaled by journal output or size (as measured by citations made)†

*Ranking*	*Journal*	$\hat{\alpha }$	$\hat{\mu }$	*Ranking*	*Journal*	$\hat{\alpha }$	$\hat{\mu }$
1	JASA	2.61	1.39	25	Biost	−0.21	0.66
2	JRSS‐B	2.11	1.95	26	AISM	−0.28	0.32
3	AoS	2.01	1.08	27	CSTM	−0.30	−0.74
4	Bka	1.93	1.09	28	Envr	−0.32	−0.11
5	CSDA	1.53	−0.34	29	CSSC	−0.34	−1.26
6	Bcs	1.43	0.68	30	ANZS	−0.40	0.06
7	JSPI	1.40	−0.45	31	LDA	−0.48	0.10
8	JMA	1.29	0.03	32	Mtka	−0.52	−0.18
9	StSin	1.28	0.37	33	JSS	−0.53	−0.80
10	StMed	1.09	−0.21	34	StMod	−0.55	−0.22
11	SJS	0.88	0.52	35	JTSA	−0.59	0.37
12	StSci	0.76	0.32	36	ISR	−0.70	0.33
13	SPL	0.74	0.01	37	JRSS‐A	−0.70	0.70
14	JCGS	0.65	0.27	38	CmpSt	−0.78	−0.64
15	Tech	0.53	0.66	39	JAS	−0.82	−1.41
16	CJS	0.36	0.23	40	SMMR	−0.85	−0.35
17	StCmp	0.07	−0.24	41	StNee	−0.85	−0.10
18	AmS	0.00	0.58	42	JABES	−0.91	−0.16
19	JSCS	−0.01	−0.65	43	Stats	−0.91	−0.65
20	Test	−0.03	−0.59	44	StPap	−1.06	−1.35
21	JRSS‐C	−0.03	0.23	45	EES	−1.51	−0.48
22	Bern	−0.05	0.17	46	JBS	−1.55	−0.83
23	BioJ	−0.10	−0.19	47	StataJ	−4.02	0.02
24	JNS	−0.21	−0.42				

†Rankings as estimated by using Poisson regression with interactions.

If quasi‐symmetry does not hold, the utility of the BT model is in question. Citation data are complex: the PM identified 209 interactions. Some of these interactions suggest heterogeneity among these journals. For example, StMed, Bes, BioJ, JBS, SMMR and Biost did not universally attract citations but cited each other quite often. We agree with the authors on the need for further statistical modelling in journal comparisons but caution that a universal ranking ignoring such heterogeneity may be inaccurate at best and misleading at worst.

**Abby Flynt** (*Bucknell University, Lewisburg*) **and Rebecca Nugent** (*Carnegie Mellon University, Pittsburgh*)

We enjoyed reading this paper and thank Varin, Cattelan and Firth for their response to Peter Hall's call for statisticians to model journal rankings better. Their successful application of the Stigler model highlights noteworthy issues in the current use of journal rankings to determine faculty research productivity, hiring practices, etc. Our comments are mostly focused on potential extensions and further incorporation of clustering information.

The authors discuss how small changes in the impact factor can cause large differences in journal rankings and that, for the Stigler model, incorporating uncertainty indicates that most of these differences are statistically insignificant. Although this is an excellent start, we suggest incorporating other knowledge in the Stigler model, possibly via covariates in modelling the export scores, such as the time reading the journal or the number of published articles (similarly to as done with the eigenfactor score). Two noted potential disadvantages of the Stigler model are that it is a ‘local’ ranking and that it does not allow for self‐citation. It would be worth devoting time to a follow‐up analysis to determine what we lose by using only the 47 selected journals.

With respect to the journals selected, the authors stress the importance of homogeneity in the group of journals for meaningful analysis of bivariate cross‐citation data. Their clustering results though seem to indicate that, although the 47 were chosen on the basis of expert knowledge, those journals are not necessarily homogeneous. What could we gain by incorporating cluster information in the Stigler model? Should we be applying the Stigler model separately to each cluster (of adequate size)? The lasso results seem to support the idea of grouped or clustered rankings, albeit with added complexity and tuning parameters. Could we do something similar but simpler with the journal cluster structure? Or, even more simply, would clustering the export scores produce similar group rankings?

Comparison of journal rankings with the research assessment exercise resulted in similar correlations for the two Stigler models and the article influence score. Computationally, the article influence score seems less sensitive to the selection of model parameters. Further, the article influence score may be more easily interpretable. Given the interdisciplinary nature of statistics, a metric that is communicable across disciplines seems vital. Moreover, since important methodological development is often published in non‐statistics journals, a cross‐discipline minimum requirement is essential if we are to represent the publishing behaviour and research influence structure in our field adequately.

**Piotr Fryzlewicz** (*London School of Economics and Political Science*)

I congratulate Varin, Cattelan and Firth on an interesting and thought‐provoking paper. My interest in the topic is partly related to my current role as Joint Editor of Series B of the *Journal of the Royal Statistical Society*; however, I have never done academic research in this area and therefore my comments are written from a non‐expert's perspective.

With the recent advances in text analysis, I believe we have the technology to analyse the ‘quality’ (‘weight’ or ‘temperature’) of citations, as opposed to their mere number. For example, arguably, the citation in ‘other recent contributions include Anon (2015)’ carries less weight, or has ‘lower temperature’ than that ‘this work is motivated by Anon (2015)’. In the same vein, a citation to a paper made once in a manuscript may be ‘less hot’ than a citation to another paper made twice.

I like the concept of ‘exporting intellectual influence’, but I do not think that analysing academic citations only is an adequate way of measuring its strength. Many statistics papers are read by data scientists outside academia, which does not lead to citations. My belief is that one way to capture part of these missing data on the intellectual influence of papers is to equip papers posted on line with discussion forums, permitting non‐academic users to discuss these pieces of work. I am particularly encouraged to make this comment in light of the conjecture made by the authors of the ‘read paper effect’—if it is true that such papers ‘export more intellectual influence’, then why not ‘make every paper a discussion paper’ by enabling an on‐line conversation about it?

In addition to citations within statistics, I believe that analysing citations between statistics and other journals could be an informative way of evaluating statistics's influence on other fields. Besides, I wonder to what extent the ‘health’ of the discipline of statistics can be measured by comparing citations to statistics journals with citations to journals in neighbouring fields such as computer science, electrical and electronic engineering or machine learning, and what lessons can be derived from such a comparison.

I shall end with a brief comment regarding the methodology used. The fused lasso is known not to be the best tool for sequence segmentation (see for example Cho and Fryzlewicz (2011) and Rojas and Wahlberg (2014)), which makes me wonder whether it is optimal or appropriate to use it to group rankings, as is done in Section 5.5 of the paper.

**Andrew Gelman** (*Columbia University, New York*)

For better or for worse, academics are fascinated by academic rankings, perhaps because most of us reached our present positions through a series of tournaments, starting with course grades and standardized tests and moving through struggles for the limited resource of publication space in top journals, peer‐reviewed grant funding and, finally, the unpredictable process of citation and reputation. As statisticians we are acutely aware of the failings of each step of the process and we find ourselves torn between the desire to scrap the whole system, arXiv style, or to reform it as suggested in the present paper. In this paper, Varin, Cattelan and Firth argue that quantitative assessment of scientific and scholarly publication is here to stay, so we might as well try to reduce the bias and variance of such assessments as much as possible.

As the above paragraph indicates, I have mixed feelings about this sort of effort and as a result I feel too paralysed to offer any serious comments on the modelling. Instead I shall offer some generic, but I hope still useful, graphics advice: Table 2 is essentially unreadable to me and is a (negative) demonstration of the principle that, just as we should not publish or include any sentences that we do not want to be read, we also should avoid publishing numbers that will not be of any use to a reader. Does anyone care, for example, that AoS has exactly 1663 citations? This sort of table cries out to be replaced by a graph (which it should be possible to construct taking up no more space than the original table; see Gelman *et al*. (2002). Table 4 represents one of the more important outputs of the research being discussed, but it too is difficult to read, requiring me to try to track different abbreviations across the page. It would be so natural to display these results as a plot with one line per journal.

I shall stop at this point and conclude by recognizing that these comments are trivial compared with the importance of the subject, but as noted above I was too torn by this topic to offer anything more.

**Amanda S. Hering** (*Colorado School of Mines, Golden*), **Emilio Porcu** (*University Federico Santa Maria, Valparaiso*) **and Moreno Bevilacqua** (*University of Valparaiso*)

We commend Varin, Cattelan and Firth for tackling the bibliometrics issue of assessing not only the metrics of statistics journals’ importance but also their uncertainty. Statisticians should clearly be involved in such work! Here, we propose additional metrics to describe important features of journals beyond scientific status.

The problems with the impact factor (IF) and its variations are well known. Employers use these indices to evaluate an employee's or potential employee's work but, to evaluate a researcher's contributions accurately, there is no substitute for reading a subset of the researcher's publications. Alternatively, the individual researcher often uses such metrics in conjunction with a variety of additional factors to decide where to submit a manuscript. First, a set of journals whose content and scope match that of the manuscript must be established. Given this subset, the importance of each journal in the field is considered. As shown clearly in Fig. 4, if these journals’ export scores do not differ significantly, other criteria must be used to make a decision. For early career statisticians, the speed of the review is particularly important. Review speed, measured in time from submission to acceptance of a manuscript, is also notoriously heavy tailed and can be manipulated. Within the discipline of statistics, many journals, particularly those with an applied theme, have sought to reduce this time (Davidian, 2013). Transparency in the distribution of review speed for journals may put more pressure on Editors to improve this feature.

The authors focus on statistics journals, but the field of statistics is unique in that it exists to advance science in other fields. Thus, we would encourage the authors also to consider metrics for the influence that statistics journals have outside the statistical community. For example, the top 100 cited articles of all time are discussed in van Noorden *et al*. (2014), and many of them describe statistical methodologies whose developments have advanced fundamental understanding in a diverse set of scientific communities. The audience that a journal typically reaches, either within or beyond the statistics community, is an important characteristic. In addition, a recent study on ‘sleeping beauties’, or papers with a sharp spike in citations later in their life, shows that not only are many of these papers in the statistics field but also many of them are multidisciplinary with a large proportion of its citations crossing from one field to another (Ke *et al*., 2015).

**Pengsheng Ji** (*University of Georgia, Athens*), **Jiashun Jin** (*Carnegie Mellon University, Pittsburgh*) **and Zheng Tracy Ke** (*University of Chicago*)

We congratulate Varin, Cattelan and Firth for a very stimulating paper. They use the Stigler model on cross‐citation data and provide a mode‐based method to rank statistical journals. Their approach allows for evaluation of uncertainty of rankings and sheds light on how to avoid overinterpretation of the insignificant difference between journal rankings.

In a related context, we study social networks for authors (instead of journals) with a data set that we collect (based on all papers in the *Annals of Statistics*,* Biometrika*, the *Journal of the American Statistical Association* and the *Journal of the Royal Statistical Society*, Series B, 2003–2012). The data set will be publicly available soon.

The data set provides a fertile ground for studying networks for statisticians. In Ji and Jin (2014) we have presented results including

- ‘hot’ authors and papers,
- many meaningful communities and
- research trends.

Here, we report results only on community detection of the citation network (for authors). Intuitively, network communities are groups of nodes that have more edges within than across (Jin, 2014). The goal of community detection is to identify such groups (i.e. clustering).

**Figure 16:**
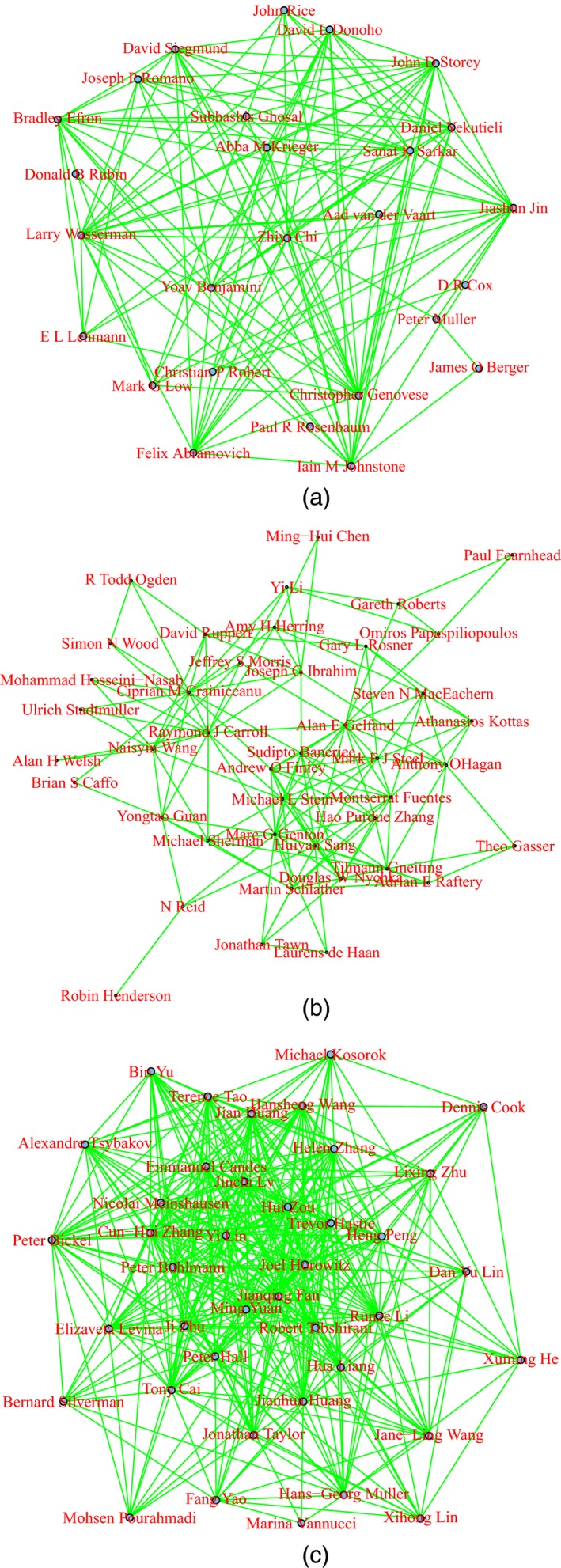
Communities found in the citation network: (a) ‘large‐scale multiple testing’ (359 nodes; only 26 nodes with 24 or more citers are shown); (b) ‘spatial statistics’ (1010 nodes; only 42 nodes with 24 or more citers are shown); (c) ‘variable selection’ community (1285 nodes; only 40 nodes with 54 or more citers are shown)

We have analysed the citation network with the method of directed scores (Ji and Jin, 2014; Jin, 2015) and identified three meaningful communities; Fig. 16. The first community is ‘large‐scale multiple testing’, including

- a Bayesian group, James Berger and Peter Müller,
- a Carnegie Mellon group, Christopher Genovese, Jiashun Jin, Isabella Verdinelli and Larry Wasserman,
- a causal inference group, Donald Rubin and Paul Rosenbaum,
- three Berkeley–Stanford groups,
  - Bradley Efron, David Siegmund and John Storey,
  - David Donoho, Iain Johnstone, Mark Low (University of Pennsylvania) and John Rice and
  - Eric Lehmann and Joseph Romano, and
- a Tel Aviv group, Felix Abramovich, Yoav Benjamini, Abba Krieger (University of Pennsylvania) and Daniel Yekutieli.

The second community is ‘spatial statistics’ and can be further split into three subgroups:

- a non‐parametric spatial statistics subgroup, including David Blei, Alan Gelfand, Yi Li and Trivellore Raghunathan;
- a parametric spatial statistics subgroup, including Tilmann Gneiting, Douglas Nychka, Anthony O'Hagan, Adrian Raftery, Nancy Reid and Michael Stein;
- a semiparametric–non‐parametric statistics (subgroup), including Raymond Carroll, Ciprian Craniceanu, David Ruppert and Naisyin Wang.

The third community is ‘variable selection’ including researchers on dimension reduction (Dennis Cook), quantile regression (Xuming He), variable selection (Peter Bickel, Peter Bühlmann, Emmanuel Candes, Jianqing Fan, Peter Hall, Trevor Hastie, Runze Li, Terrence Tao, Robert Tibshirani, Alexandre Tsybakov, Ming Yuan, Cun‐Hui Zhang, Ji Zhu and Hui Zou).

Our results must be interpreted with caution, for the scope of the data set is limited. Also, it is not our intention to rank authors or papers.

**Jon R. Kettenring** (*Drew University, Madison*)

This paper provides an excellent comprehensive discussion of citation analysis with emphasis on ranking journals. Citations are a weak form of data. The hope is that the data will nevertheless be sufficiently rich to produce useful insights.

With this in mind, my comments focus on Section 3, clustering journals, which the authors suggest ‘can help to establish relatively homogeneous subsets of journals that might reasonably be ranked together’.

A complete linkage hierarchical clustering algorithm is used to produce the dendrogram shown in Fig. 2. Six clusters and two singletons are identified by cutting the tree. How well determined are they? For comparison, I repeated the analysis by using the average and minimax (Bien and Tibshirani, 2011) linkage algorithms. The results for the average method match up especially well. The shaded branches in Fig. 2 are all evident as are the singletons, with *StataJ* joining at the very last step. The minimax dendrogram has two major branches and somewhat different details. The 11‐journal cluster (applications, health) in Fig. 2 is embedded in one of them. Its ‘prototype member’ Bcs does not reflect the diversity of the other 10. In the companion main branch, there are neighbouring subbranches consisting of (Bern, Test, AoS and StSin) and (JCGS, StCmp, CJS, SJS, JRSS‐B, Bka and JASA).

Non‐metric multi‐dimensional scaling provides another way to view the journals, without forcing any clustering. A scatter plot of the two‐dimensional multi‐dimensional scaling solution (14.27% stress) suggests clusters that only partially match Fig. 2 but confirms visually the two singletons. Some of the close neighbours make intuitive sense (e.g. Bka, JASA and JRSS‐B). The journal JRSS‐A is closest to StNee but why? The three‐dimensional solution (9.21% stress) provides similar insights with ISR and JABES straying from the crowd.

All of these analyses hinge on $\rho_{ij}$ which is problematic, even though it is a popular approach (Boyack *et al*., 2005). The underlying bivariate scatter plots are heavily skewed with a pile‐up of points near the origin and large outliers in some cases. These are not natural situations to summarize by using Pearson correlation coefficients.

Another drawback is that these analyses do not differentiate between exported and imported citations since it is the total numbers exchanged‐between pairs of journals that are used.

Even granting these limitations, the clustering approach may still meet the authors’ stated objective of identifying groupings of journals that are sufficiently good for developing rankings.

**Chenlei Leng** (*University of Warwick, Coventry*) **and Ting Yan** (*Central China Normal University, Wuhan*)

We congratulate Varin, Cattelan and Firth for a thought‐provoking paper. We discuss two issues related to the methodology used to analyse the exchange of citations.

This paper proposed an interesting ‘quasi‐Stigler’ model to rank scientific journals, in which each journal is assigned a merit parameter. In contrast with the ‘Stigler’ model by Stigler (1994) that used the Bradley–Terry model to rank statistical journals, the new method does not make the independence assumption among exchanged citations for all pairwise journals. This quasi‐Stigler model without assuming independence can potentially be used in a more general context, e.g. to rank teams in sport tournaments.

If all exchanged citations between journals, $t_{ij}$s, go to ∞, and the number of journals, *n*, is fixed, then the consistency and asymptotic normality of the quasi‐likelihood estimators in the quasi‐Stigler model are expected. These assumptions, however, may not hold in the exchanged citations data, as the citation counts between some weak journals are small. For this reason, it is not clear whether the alleged asymptotic properties of the quasi‐estimators hold. When $t_{ij}$s are bounded and *n* →∞, the asymptotic properties of the maximum likelihood estimate in the Bradley–Terry model (Simons and Yao, 1999) and some network models (Chatterjee *et al*., 2011; Yan and Xu, 2013; Yan *et al*., 2015) are now understood. It will be interesting to see whether similar properties can be established for the quasi‐estimators in this different asymptotic regime.

The ranking lasso for clustering journals would be useful if the asymptotic results hold. However, the solution paths in Fig. 5 do not cross over, suggesting that a simpler alternative to the lasso may be sufficient for grouping journals. In particular, we can aggregate journals into groups by using a procedure akin to hierarchical clustering. For the first step of this procedure, we can group the two journals with the closest merit parameters and re‐estimate the quasi‐Stigler model with one fewer parameters. Then we keep aggregating groups with the closest merit parameters. In the end, we have a sequence of models and one can use the Takeuchi information criterion in the paper for choosing the final model. A different criterion is to use a *t*‐statistic between neighbouring journals according to the estimated merit parameters and to aggregate the two journals that have the smallest *t*‐statistic. This simpler procedure may also provide an easier way for statistical inference.

**Han Liu** (*Princeton University*)

We congratulate Varin, Cattelan and Firth for a thought‐provoking contribution on analysing citation data between statistical journals. Here I make three comments. The first suggests a more comprehensive analysis by including more journals from related fields. The second suggests that more information should be incorporated in the analysis in addition to the citation exchange data. The third suggests a possible approach to conduct uncertainty assessment for the fitted Stigler model with regularization.

First, the current analysis includes only 47 statistics journals. It would be interesting to see how the results change if we include the citation data from more journals in related fields. Since statistics is becoming increasingly important in the scientific community, there is a much wider venue to publish statistics papers than before. Taking myself as an example, in addition to publishing in statistics journals (e.g. the *Journal of the Royal Statistical Society*, Series B, the *Annals of Statistics*, the *Journal of the American Statistical Association*,* Biometrika*,* Bernoulli* and the *Journal of Computational and Graphical Statistics*), I also publish statistics papers in machine learning journals (e.g. the *Journal of Machine Learning* and the *IEEE Transactions on Pattern Recognition and Machine Intelligence*), optimization journals (the *SIAM Journal on Optimization and Mathematical Programming*) and electrical engineering journals (e.g. the *IEEE Transactions on Information Theory* and the *IEEE Transactions on Automatic Control*). A more thorough analysis with these related journals included may provide new insight. In addition, many machine learning researchers publish their major results in conference proceedings (e.g. Neural Information Processing Systems, the International Conferences on Machine Learning and the Conferences on Artificial Intelligence and Statistics). These conference publications have much shorter review periods and bigger capacity than the statistical journals. It is interesting to build more sophisticated models that naturally handle both conference and journal data.

Second, the current analysis exploits only the citation exchange data. In addition to the citation data, the raw texts of the papers are also easily accessible. It would be interesting to see how the unstructured raw text can be incorporated in the existing models to provide more refined analysis of journal influence. This essentially requires natural language understanding and artificial intelligence. The current deep learning technique (Socher, 2014) has shed some light in this direction.

Third, there is no uncertainty assessment (e.g. constructing confidence intervals and testing hypotheses) for the fitted Stigler model using the ranking lasso penalty. There are two challenges:

- we need to handle penalized peudolikelihood instead of penalized likelihood;
- the fused lasso penalty introduces a non‐negligible estimation bias.

A recently proposed decorrelated score inference method in Ning and Liu (2014) seems to be applicable in this setting.

**Nicholas T. Longford** (*SNTL, Barcelona*)

The paper implies a worrying future for statistics as a scientific field. Networking and ‘conspiracy’ in publication on similar subjects will gradually take over from the original scientific and academic priorities. As soon as a metric has been adopted for classifying an individual's or a department's performance, we adjust our research and publication strategies, first not to be disadvantaged by the metric, and then to exploit the surrogate nature of the metric. The result will be an inflation of the metric and soon we shall all be above average. (Overheard at the coffee break: ‘This is a good paper. Why don’t you ask a few people to cite it?’)

The metrics studied by Varin, Cattelan and Firth appear to be appropriate at present, or in the recent past, but the entire methodology presented by them ignores the ‘human’ factor or interference—the likely systematric effort to publish ‘for the metric’, and not in pursuit of core academic priorities. When a metric has been adopted the (presumed) close association of the indices studied in this paper with the quality of our output will be undermined. This change will not be observed because we shall have no other practical means of assessing the quality of such output, other than the index.

The assessment of the four top journals in Fig. 4 confuses magnitude with significance. The qualifier ‘outstanding’ may be interpreted as ‘far ahead of the others’. The nominal significance in the diagram is a poor argument for this, given the short confidence intervals. Suppose that the underlying quantities $\mu_{i}$ are a realization of a random sample from a normal distribution with known variance $\sigma^{2}$. Then the difference of the maximum and the second highest value in this sample has expectation 0.40*σ* and standard deviation 0.36*σ*, estimated by simulations. The 95th percentile of its distribution is 1.12*σ*. So, the *Journal of the Royal Statistical Society*, Series B, is very likely to have the highest value of the metric but, given that it is the highest, it is not exceptional. The ‘outstanding’ nature of the first four journals can be studied similarly.

**Jorge Mateu** (*University Jaume I, Castellón*)

Varin, Cattelan and Firth are to be congratulated on a valuable contribution and thought‐provoking paper in this timely topic of ranking scholarly journals. This clearly involves a twofold problem: an economical one for librarians, and a scientific one for researchers. I prefer to focus my comments on the latter case.

The Stigler model is at the core of the statistical principles followed in this paper. This is a good model proved to be valid in practice, but it has some open directions for improvement.

It is quite obvious that the binomial variables $C_{ij}$ are not independent of each other and dependence is expected through both components of the expected value. $t_{ij}$ are clearly biased within journals of the same group as suggested by the cluster analysis. Indeed we expect that journals ranking from middle to low positions of the ‘Journals citation reports’ will tend to cite top position journals, but not vice versa. This bias is not completely accounted for by using a quasi‐likelihood method over an added overdispersion factor. A more robust and adapted Stigler method could be defined.

Along this line, I argue that the strategy of considering discussion and review papers enlarges the counts $t_{ij}$, and I wonder whether these types of contribution should be downweighted in their contribution to the $C_{ij}$.

It is highly important that differences in the individual journal ranks are not reliable nor significant. A way to discriminate between non‐significant rank‐based position in the ‘Journals citation reports’ list and those who are really reliable is urgently needed. Group‐based ranks could be a solution. In this case, a journal leaving a particular group to its more immediate lower group is more revealing than just shifting one position in any direction. The implementation of this option is simple within the Stigler model framework.

Finally, we advocate the use of a more dynamic model to favour the evolution in time of the rankings. Much more information is contained in a 10‐year period of the rankings than in one particular year. The evolution reports facts over journals which are hidden in particular snapshots. Along this line, the extension of the Stigler method goes through not only considering the dependence on time, but also past dependences in an auto‐regressive structure. This is an interesting model to consider which can be more realistic.

**Weining Shen** (*University of California at Irvine and University of Texas MD Anderson Cancer Center, Houston*) **and Dehan Kong** (*University of North Carolina at Chapel Hill*)

We congratulate Varin, Cattelan and Firth for their thought‐provoking work in journal citation data analysis. They have made a substantial contribution by modelling the cross‐citation table such that journals are evaluated (ranked or clustered) on an objective scale, and by considering a flexible inference procedure (a quasi‐Stigler model) that allows uncertainty quantification and shrinkage estimation for grouping. We would like to discuss some possible extensions of the current work. First, removing journal self‐citations may lead to unfavourable evaluations for ‘top’ journals because publications in good journals are assumed to have higher impacts and hence are more likely to be cited. For example, in Table 2, the self‐citation proportion is 17% for the *Annals of Statistics*, which is higher than that of a few other theory‐oriented journals. It might be useful to consider positive diagonal loadings (related to the ranks) of matrix **C**, or by only removing these self‐citations made by the same authors. Secondly, it is unclear how good the approximation is when calculating the quasi‐standard errors of export scores. Comparison with alternative approaches such as the bootstrap or a beta–binomial model under the Bayesian framework may be helpful. Thirdly, the authors used the *z*‐statistic to test the statistical difference between different journals. It would be interesting to develop a multiple‐testing procedure for these tests. Finally, as pointed out by the authors in Section 7.4.2, a dynamic evaluation of export scores and journal rankings over time will be helpful. A look at the 5‐year citation data will bring in some recently established journals of high quality such as the *Annals of Applied Statistics*,* Bayesian Analysis* and the *Electronic Journal of Statistics*.

**Arthur Spirling** (*New York University*)

### Motivation and measurement

Like any measurement problem, ranking journals involves judgements about the reliability and validity of the metric. On the former, more information about how rankings might change (suddenly?) over time and how sensitive they are to a few ‘big’ outlier articles in a given year is warranted. On validity, the fact that the authors’ measurement strategy is deemed successful in part because it returns the ‘big four’ statistics journals at the head of the rank order should be interpreted with caution to ensure that we do not reward methods for simply reproducing our priors with the data.

The Bradley–Terry model seems straightforward to fit and has obvious benefits; but the decision over *which* journals to compare is consequential, disputable and requires domain expertise. There is a danger that the original problem of ‘*how* should we compare journals?’ is replaced with ‘*what* journals should we compare?’—which may be a very thorny issue! Related to this, is there not a way to use the generalized linear model linear predictor, or indeed a random effect, to ‘control’ for discipline or subject matter in estimating the influence of a journal?

The authors’ motivation is partly ‘economic’ in so far as librarians must make choices about the journals they subscribe to. As more journals move towards an ‘open access’ model, this justification seems less important, although perhaps the information will still be helpful for submitting authors.

### Extensions

The authors’ efforts can certainly be used in other fields: Carter and Spirling (2008) attempted something similar a few years ago. In that case, we noted that discipline‐specific practices—such as a focus on books along with articles as a venue for information transmission—can make ranking journals less helpful as a way to assess research output. Furthermore, journal‐specific practices in some disciplines—including the encouragement away from long literature reviews for certain outlets—can cause obvious problems for an ‘import–export’ metric that assumes ‘fair trade’ across journals.

On the statistical side, a more explicitly Bayesian approach might allow for

- a cost function to penalize ‘incorrect’ decisions by for example librarians, given the ranking uncertainty,
- the addition of discipline random effects without the need for numerical integration methods and
- a natural way to incorporate (expert) prior information on the relative prestige of outlets.

**Stephen M. Stigler** (*University of Chicago*)

Varin, Cattelan and Firth raise model‐based citation analyses to a new high level, with a judicious mix of convincing arguments for the method's strengths and realistic assessments of problem areas. Theirs is an exemplary study. The paper is data rich so I may be excused in offering as additional evidence an anecdote.

I wrote my first paper on a citation analysis about 40 years ago: a study of the citations in the work of Pierre Simon Laplace (1749–1827) and the intellectual influences and changes over time in his work that could be deduced from them (Stigler, 1978). Lacking a database I resorted to handwork. I went through all 14 volumes of his collected works and noted every mention of a name that could be taken as an implicit reference (citation practices were quite different then). I sent a draft manuscript to my friend Fred Mosteller. He replied politely on January 28th, 1975, with no evident enthusiasm:

‘I note that in my own case I have rarely cited S. S. Wilks (except in writings about his life) in my own technical works. Still, he was absolutely fundamental to my development. This suggests to me that one can't put much faith in the frequency of citations for assessing intellectual leadership. Still, probably better than nothing.’

On February 3rd I replied,

‘The more I've looked at citations, the more I’ve come to trust them. Like all methods of measurement, they require cautious interpretation, and there is a question of exactly what it is they are measuring. But I think that they measure something *like* intellectual influences (plus bias plus individual effects, field effects, age effects, time effects, etc.), and are worth study. Just for fun, I counted the citations in your “On Some Useful “‘Inefficient”’ “Statistics,” using the same approach I used with Laplace: each time a *name* is mentioned counts as a citation. I was unsure of how to deal with the “References” list, as Laplace had none, so I did the count with and without it. Without the reference list included, Wilks is tied for third place, one behind Fisher and Karl Pearson! Not a bad group to be in with! Also of interest are the names not on the list—Neyman, E. S. Pearson, Cramér, Wald.’

In reply, Fred said he regretted missing Wald.

I hope that this study will be greeted with the enthusiasm it deserves, and inspire even more exploration in this fascinating topic.

**Peter F. Thall** (*University of Texas MD Anderson Cancer Center*,* Houston*)

Varin, Cattelan and Firth have provided a thoughtful and informative analysis of journal citation data in the form of a cross‐citation count matrix **C** of citations exchanged between pairs of journals in a set selected to be homogeneous. Their decisions about what should be included when constructing **C** make practical sense, but recent rapid development and advances in areas such as bioinformatics, machine learning and Bayesian non‐parametric statistics make me wonder what an analysis like that given here, but including important newer journals, might show. Much of the recent scientific and societal influence of statistics seems to be coming from newer areas. Historically, new developments in statistics often have been driven by needs in other areas of science and, more recently, by computational advances that make what previously was impossible now feasible. The noted Thomson Reuters ‘immediacy index’ seems to address this issue, but its 1‐year time period seems too limited.

For article influence scores, the probability *λ* is used as a tuning parameter to mix the normalized citation matrix and the matrix having identical columns that are the normalized numbers of articles, to form the matrix *P* and to compute its eigenvalues. Since this plays a central role in defining the article influences rather than simply setting *λ*=0.85, I wonder what a sensitivity analysis with the article influences varying as functions of *λ* might show. Table 4 is very informative, but I wonder what the posteriors of the ranks would look like, after doing either Bayesian model averaging or model selection for the set of approaches, under a suitable Bayesian or empirical Bayes formulation, possibly with a hierarchical structure to induce shrinkage among journals. This might help to quantify uncertainty about models and ranks. It would be interesting to see whether, for example, such an analysis recapitulates Fig. 4. But this is just a different methodological perspective. The authors’ analyses not only give useful insights into how the journals selected may be clustered and ranked, but they also provide an instructive case‐study of how one goes about practical application of quasi‐likelihood methods in general.

The **authors** replied later, in writing, as follows.

It is pleasing to see so much interest in this work, and we thank all the discussants for thoughtful comments. Our reply here is necessarily brief, and we are sorry not to be able to respond directly to all contributors. We shall make a few general comments before we identify some main themes and make a few remarks on each.

We hope that our paper is sufficiently clear in regard to the limitations of citation analysis, and in particular why journal rankings should not be used to evaluate the work of individual researchers. Various discussants (Colquhoun, Aston, Arbel and Robert, Darroch, and Hering, Porcu and Bevilacqua) have echoed our concerns about this aspect of ’bibliometrics’. Conclusions drawn from these concerns are rather polarized, ranging from Colquhoun's ‘more research in this area cannot be justified’, through to opinions in line with our own view that good statistical methodology can and should inform potential users of journal rankings about the limitations. We disagree completely with Longford's view that our paper implies a worrying future for statistics as a scientific field; his point about publishing ‘for the metric’ is well taken, though, and seems especially relevant where metrics are applied to individuals.

With such limitations always in mind, we find ourselves in agreement with Stigler, about citation data: ‘… they measure something *like* intellectual influences (plus bias plus individual effects, field effects, age effects, time effects, etc.), and are worth study’. The Stigler model's export scores are best viewed as reflecting the ‘balance of trade’ among journals (Stigler, 1994) rather than the more difficult (as mentioned by Aston) notion that each interjournal citation is the outcome of a ‘contest’ between two journals. The export scores measure intellectual influence, and we believe that this accounts for the Stigler model's success. The appearance of prestigious journals at the top of the Stigler model ranking is thus, as remarked by Colquhoun, not surprising—indeed, it is reassuring! Not all journal ranking methods have such a clear rationale. For example the ‘new kid on the block’, a Google Scholar ranking of journals announced in June 2015 (see http://googlescholar.blogspot.co.uk/2015/06/2015-scholar-metrics-released.html), has eight other journals ahead of the *Journal of the Royal Statistical Society*, Series B, in its ‘Probability and statistics’ category, but it is unclear *in what way* those eight should be thought of as ‘better’ (although it does seem that journal size is quite important there).

We are especially happy that at least five of the discussion contributions used the data set that we made available (with the kind permission of Thomson Reuters), to study extended models and alternative approaches. Preparing the data for publication, with associated code for reproducibility of results, was a substantial aspect of our work on this paper, and we hope that it proves a useful resource for future researchers. Already the new analyses that were reported by Wyse and White (a stochastic block model), Carlen and Handcock (a network Poisson model), Dorman and Maitra (sparse modelling of quasi‐symmetry departures), Arbel and Robert (graphs of the citation network) and Kettenring (alternative clustering methods) have amply rewarded our efforts to make the data accessible.

### Journal selection and field normalization

Many discussants (Arbel and Robert, Aston, Bray and Song, de Carvalho, Cocchi, Darroch, Flynt and Nugent, Fryzlewicz, Hering, Porcu and Bevilacqua, Liu, Spirling and Thall) question the selection of journals. Aston makes the good point that considerable information is lost through *any* selection of journals, given the high density of the network of interjournal citations.

The list of selected journals was motivated by our wish to provide a coherent illustration of the Stigler modelling approach within the ‘Journal citation reports’ category of ‘Statistics and probability’. However, we agree with discussants about the limitations of our exercise. The value of statistical research is undoubtedly measured also, and perhaps indeed primarily, by its influence in other disciplines; and this is not directly measured by our application of the Stigler model to statistics journals only.

Although we understand the convenience for administrators of having available a global ranking of journals from all disciplines, we continue to be sceptical about the value of such a ranking. We agree with Colquhoun that proper normalization of citation data across all fields is impossible, though one might perhaps attempt some form of model‐based normalization for sets of journals that are sufficiently homogeneous to be coherently analysed together. In this direction, we appreciated the proposed extensions of the Stigler model from Bartolucci and Spirling. The addition of ‘discrimination parameters’ (Bartolucci) or discipline‐specific random effects (Spirling) seems a sensible way to incorportate a measure of journal heterogeneity in the Stigler model. This appears likely to be a fruitful area for further research, which we plan to pursue.

### How many years?

An issue that was raised in several contributions (Colquhoun, Aston, Bray and Song, Darroch, and Shen and Kong) concerns the time period used in the analyses. We note that there are two different time windows involved in citation data analysis: the time period of publication of articles whose references are collected—in our data, 1 year—and the time period of publication of articles that are cited (either 1, 2, 5 or 10 years). It seems that the 1‐year time period for collecting references is not questioned as much as the years of publication of cited papers. However, as pointed out by Bray and Song, it may lead to ‘year‐specific results’. Enlarging this time window might perhaps lead to more stable results, but it would also require either, at least partially, to overlap the period of publication of citing and cited papers, or to consider references to papers further back in time, thus losing citations to papers published in the previous year(s).

A larger debate regards the number of years for cited papers considered in the analyses. The available data allow to compute citations for time windows ranging from 1 to 10 years, or to use all‐years citations. As mentioned in the paper, some authors have shown that a 2‐year time period fails to capture the citation behaviour of statistical and mathematical papers. Indeed, the current main competitor of the impact factor, the article influence score, is computed on a 5‐year time period, and the impact factor also now has a 5‐year version. We used the 10‐year time period to capture the influence of journals over a substantial period, considering also that statistical papers typically have long‐term citation behaviours, as evidenced by the median age of citations given and received by statistical papers, which exceeds 5 years for most statistics journals.

We note also that different disciplines require different time windows to reflect properly the citation behaviour of their area. The expected substantial differences between appropriate time windows for different disciplines seem to us a major hindrance to any ‘global’ analysis that involves journals from multiple research fields.

### Role of negative or redundant citations

A few discussants (Arbel and Robert, Fryzlewicz, Liu and Murtagh) mention that it could be desirable to account for what is commonly termed ‘citation behaviour’ in the bibliometrics literature (e.g. Case and Higgins (2000)). Currently available studies mostly indicate that papers receiving negative citations are relatively infrequent and are characterized by a ‘rapid rate of decay’ in citations (Hull, 1988). More concern should be perhaps addressed to the frequent redundant and/or cursory citations that are often seen in the introductory sections of papers (Bornmann and Daniel, 2008).

Although it is clear that not all citations made in a paper have the same weight or meaning, we think it unlikely that the distribution of different citation types (negative, redundant or positive), across journals, would appreciably impact statistical modelling at the aggregated level of whole journals. The distribution is currently unclear, though. For example, prestigious journals might be thought to publish fewer papers that attract negative citations; however, papers published in prestigious journals are more visible and thus perhaps more prone to being negatively cited.

As suggested by Fryzlewicz, Liu and Murtagh, full‐text access to papers would allow evaluation of the content and the semantics of citations, and thus assessment of our (implicit) assumption that citation types are uniformly distributed among journals. Without that assumption, any bias due to citation behaviour should be accounted for through suitable modification of the Stigler model or indeed any other statistical model of citation exchange.

### The Stigler model

Agresti observes that standard errors based on quasi‐likelihood may not be robust if the citations exchanged between two journals follow a beta–binomial model constructed from equicorrelated Bernoulli variables. The same concern has been mentioned to us, in a private communication, by Dr P. M. E. Altham. Indeed, under the equicorrelated beta–binomial model the variance of $C_{ij}$ is

$$\text{var}\left(C_{ij}\right)=t_{ij}\pi_{ij}\left(1-\pi_{ij}\right)\left\{1+\left(t_{ij}-1\right)\gamma_{ij}\right\},$$

with $\gamma_{ij}$ being the correlation between single citations from journal *j* to journal *i*. Agresti and Altham correctly say that the simple inflated variance of our paper may not be appropriate because totals $t_{ij}$ vary considerably across pairs of journals. However, as noted also by Altham (private communication) the issue may not be relevant if the correlation $\gamma_{ij}$ decreases as $t_{ij}$ increases. Indeed, we would expect that, the larger the degree of communication between two journals, the smaller the correlation between single citations exchanged by the journals. Unfortunately, we do not have data at the level of detail that would allow estimation of the various $\gamma_{ij}$ and thus validation of our model for the variance. Along the same lines, Aston suggests considering multiple dispersion parameters in the quasi‐likelihood, whereas Shen and Kong propose comparison of our quasi‐standard errors with a beta–binomial model fitted in a Bayesian framework. The multiple‐dispersion model could perhaps be estimated with the available journal level data under suitable constraints designed to identify clusters of journal heterogeneity.

Bray and Song question the use of a diagonal covariance matrix in the quasi‐likelihood estimating equation, stating that citation counts $C_{ij}$ are not independent. We do not think that the issue has an appreciable effect on our analysis: the Stigler model supposes that the ratios $C_{ij}/t_{ij}$ are conditionally independent between pairs of journals, which seems a reasonable modelling assumption; and the dependence between the single citations composing $C_{ij}$ is accounted for through the dispersion parameter. Moreover, the analysis of residuals does not indicate any particular discrepancy with the assumptions made (although we did not check for this specific departure). However, dependence between the $C_{ij}$ may become relevant in a ‘structured’ Stigler model where the export scores $\mu_{i}$ are described as a function of covariates chosen to account for confounder effects, as suggested by Bray and Song, de Carvalho, and Flynt and Nugent. See Cattelan and Varin (2013) for an illustration of quasi‐likelihood estimation, with a non‐diagonal variance, in a structured Bradley–Terry model.

Proper modelling of the dependence structure is certainly crucial in dynamic extensions of the Stigler model, as mentioned by discussants Mateu, de Carvalho, and Shen and Kong, In this context, we agree with de Carvalho that temporal variations in export scores might be modelled interestingly in terms of suitable covariates, which perhaps attempt to capture time varying aspects of journals’ editorial policies, etc.

There are several interesting suggestions about extension or modification of the Stigler model. We agree with Carlen and Handcock that their Poisson graph model is attractive in terms of interpretation and extensibility. Bartolucci, and Dorman and Maitra propose additional parameters that depend on the specific pairwise comparison, to capture departures from the assumed quasi‐symmetry model. Although the data analysed in our paper appear to satisfy quite well the quasi‐symmetry assumption, it seems likely that with larger data sets and/or more heterogeneity such extensions would become more relevant.

The ‘journal residuals’ that are defined in the paper, designed to detect a particular type of departure from the Bradley–Terry model, undoubtedly have potential for improvement. One possibility is the use of standardized residuals suggested by Agresti, but we note that the leverages in an unstructured Bradley–Terry model such as ours are not prone to high variability so the effect of such a change would often be quite small.

Shen and Kong are concerned about the effect of ‘removing journal self‐citations’ which, in their view, may lead to unfair evaluation of the most prestigious journals. We wish to emphasize that journal self‐citations are *not* removed from the data: they simply play no role in the Stigler model that describes the ‘balance of trade’ (Stigler, 1994) between journals. However, we do agree with Shen and Kong that it would be interesting to account for *author* self‐citations. Refitting the Stigler model with author self‐citations omitted would allow the stability of estimated export scores to be checked, though it might then be necessary also to account for the effect of networks of authors who cite one another (as mentioned privately to us by Professor R. J. Carroll). We note that the effect of citations exchanged within networks of authors is likely to be critical when comparing fields characterized by different typical numbers of authors per paper.

Leng and Yan raise an interesting point about the possibility of using a different asymptotic framework to derive the approximate distribution of estimated export scores. We note here only that the quasi‐likelihood estimator solves the same linear estimating equations as the standard maximum likelihood method, and it would be surprising if there are not fairly mild conditions under which the results of Simons and Yao (1999), for example, continue to hold with only simple modification for overdispersion.

### Lasso and other penalties

Reflecting the strong current interest in sparse methods, many discussants (Agresti, Aston, Bartolucci, Bray and Song, Flynt and Nugent, Fryzlewicz, Leng and Yan, Liu and Thall) comment on shrinkage estimation of the Stigler model. Our lasso exercise was designed to illustrate that many apparent differences between estimated journal export scores are indeed negligible in predictive terms. In this sense, the lasso fitting of the Stigler model may be interpreted as an alternative to, but perhaps not a substitute for, a multiple‐testing approach as suggested by Colquhoun, and Shen and Kong. We agree with Aston and Thall that other types of shrinkage penalty, such as ridge regression or empirical Bayes, may be at least equally appealing for prediction.

Uncertainty quantification for lasso estimates is a very active topic of current research; see for example Tibshirani *et al*. (2015) and references therein. The decorrelated score approach suggested by Liu seems promising and we would be interested to see an application to the ranking lasso. Agresti suggests a bootstrap approach to derive non‐symmetric confidence intervals. One potential issue with a non‐parametric bootstrap is the difficulty of decomposing bivariate citation data into independent or pseudoindependent blocks. For that reason, Masarotto and Varin (2012) considered parametric bootstrap confidence intervals around the pairwise differences of export scores.

Fryzlewicz questions the optimality or appropriateness of the fused lasso penalty for grouping rankings, in view of available results in sequence segmentation. We are unsure whether these, otherwise sensible, concerns apply also to our context where the model is itself identified in relative terms through pairwise differences $\mu_{i}-\mu_{j}$. For the same reason, the suggestion by Bartolucci to consider alternative penalties that avoid shrinkage to 0 may not be suitable because the Stigler model is necessarily fitted under an arbitrary constraint, such as the sum constraint $\Sigma_{i=1}^{n}.6pt\mu_{i}=0$ that is employed in our paper.

Bray and Song, Flynt and Nugent, and Leng and Yan suggest hierarchical clustering of close export scores. We see this as a very sensible alternative to the adaptive ranking lasso method that is discussed in the paper. As observed by Leng and Yan, a crucial condition for conformity between this proposal and the ranking lasso is that the path of the latter is free of crossings. Simulation studies performed at the time of writing Masarotto and Varin (2012) indicate that in many instances the path does respect this assumption, although it is unclear to what extent this property is related to the amount of information that is available for estimation of each export score.

### Clustering journals

We thank discussants Kettenring, and Bray and Song for pointing out that measurement of interjournal distance through Pearson correlation may not be the most appropriate basis for a cluster analysis, because of the ubiquitous skew distribution of citations. We should emphasize, though, that the clusters that were identified were not at all central to our work and had no effect on the paper's main results. Clearly there are many other clustering approaches that could be used in such an exploratory way, perhaps leading to different insights on journal interrelationships.

### Presentation of graphs and tables

We are very grateful for Gelman's critique (private communication) of some of the plots and tables that appear in the paper. To our embarrassment, we find ourselves in broad agreement with his comments! We had drawn the dendrogram (Fig. 2) in a way that was unsuitable for the journal's page format, and regrettably it became almost unreadable in the preprints that were available to discussants; this has been corrected in the final version of the paper. In light of Gelman's comment, in the final version of the paper we also adjusted Fig. 1, to make at least some use of the vertical dimension. These changes, made at proof correction stage, are necessarily rather minimal. We should of course have paid closer attention to these aspects of the paper's presentation in advance; we agree completely that graphs and tables in statistical work should be crafted to be as readily informative as possible.

To conclude, we reiterate the aspiration that we expressed at the meeting, namely that this paper might succeed in getting a few more of our statistical research colleagues interested in this area of work. We earnestly hope that principled, statistical approaches to the whole topic area of ‘bibliometrics’ will quickly become the norm rather than the exception, and that the published journal rankings themselves will either improve substantially or become universally discredited.

## Supporting information

‘Supplement to “Statistical modelling of citation exchange between statistics journals”’.
