# Supplementary material for: Statistical modelling of citation exchange between statistics journals
Source: J R Stat Soc Ser A Stat Soc. 2015 Nov 3;179(1):1–63. doi: 10.1111/rssa.12124 (PMC4755202; doi:10.1111/rssa.12124)
Supplement: Supplementary file 1 — ‘Supplement to “Statistical modelling of citation exchange between statistics journals”’. [file RSSA-179-1-s001.zip › Varin_Cattelan_Firth_supplement.pdf]

Supplement to 'Statistical Modelling of Citation Exchange Between Statistics Journals'

Cristiano Varin, Manuela Cattelan and David Firth

This document illustrates the R (R Core Team, 2015) code accompanying Varin, Cattelan and Firth (2015). The figures and tables in the paper differ in minor respects from those produced in this document due to some manual editing for inclusion in the paper.

1 Cross-citation data

The 47×47 cross-citation matrix $\mathbf{C} = [c_{ij}]$ is in file `cross-citation-matrix.csv`:

```
Cmatrix <- as.matrix(read.csv("Data/cross-citation-matrix.csv",
                              row.names = 1))
```

Journals are identified in $\mathbf{C}$ through the journal abbreviations listed in Table 1 of the paper:

```
journal.abbr <- rownames(Cmatrix)
journal.abbr

##  [1] "AmS"    "AISM"   "AoS"    "ANZS"   "Bern"   "BioJ"   "Bcs"
##  [8] "Bka"    "Biost"  "CJS"    "CSSC"   "CSTM"   "CmpSt"  "CSDA"
## [15] "EES"    "Envr"   "ISR"    "JABES"  "JASA"   "JAS"    "JBS"
## [22] "JCGS"   "JMA"    "JNS"    "JRSS-A" "JRSS-B" "JRSS-C" "JSCS"
## [29] "JSPI"   "JSS"    "JTSA"   "LDA"    "Mtka"   "SJS"    "StataJ"
## [36] "StCmp"  "Stats"  "StMed"  "SMMR"   "StMod"  "StNee"  "StPap"
## [43] "SPL"    "StSci"  "StSin"  "Tech"   "Test"
```

2 Cluster analysis

Computation of the matrix of the total number of citations exchanged between pairs of journals $\mathbf{T} = [t_{ij}]$ defined in formula (1) of the paper:

```
Tmatrix <- Cmatrix + t(Cmatrix)
diag(Tmatrix) <- diag(Cmatrix)
```

Hierchical clustering of journals with complete linkage using distance $d_{ij} = 1 - \rho_{ij}$, where ρ_{ij} is the Pearson correlation between journals i and j:

```
journals.cluster <- hclust(d = as.dist(1 - cor(Tmatrix)))
```

Dendrogram (Figure 1 of this document):

```
plot(journals.cluster, sub = "", xlab = "")
```

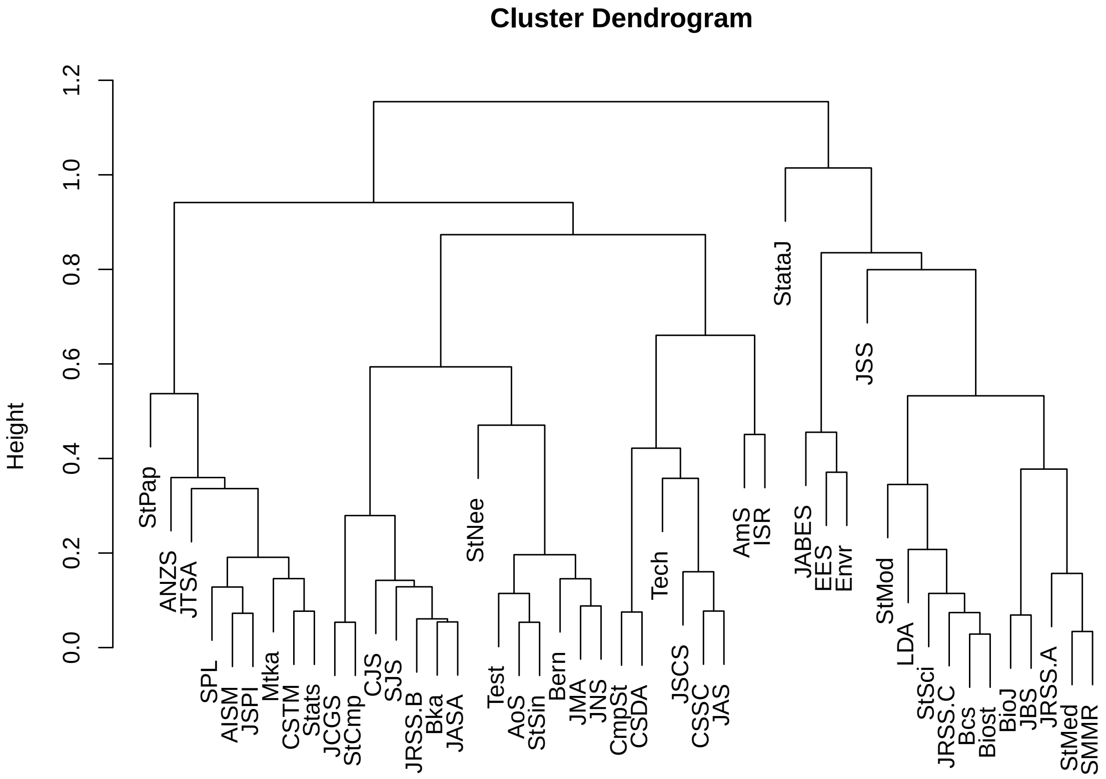

Figure 1: Dendrogram of the hierarchical cluster analysis of journals.

3 Quasi-Stigler model

The quasi-Stigler model is fitted with the BradleyTerry2 package (Turner and Firth, 2012):

```
require(BradleyTerry2)
```

Re-arrange data in a form suitable for the BradleyTerry2 package:

```
Cdata <- countsToBinomial(Cmatrix)
```

Fit the model:

```
fit <- BTm(outcome = cbind(win1, win2),
           player1 = player1, player2 = player2, data = Cdata)
```

Estimation of the overdispersion parameter defined in formula (7) of the paper:

```
npairs <- NROW(Cdata)
njournals <- nlevels(Cdata$player1)
phi <- sum(residuals(fit, "pearson")^2) / (npairs - (njournals - 1))
phi

## [1] 1.759027
```

3.1 Journal residuals

Computation of the 'journal residuals' discussed in Section 5.2 of the paper:

```
journal.res <- rep(NA, njournals)
res <- residuals(fit, type = "pearson")
coefs <- c(0, coef(fit)) # 0 is the coefficient of the first journal
for(i in 1:njournals){
    A <- which(Cdata$player1 == journal.abbr[i])
    B <- which(Cdata$player2 == journal.abbr[i])
    y <- c(res[A], -res[B])
    x <- c(-coefs[Cdata$player2[A]], -coefs[Cdata$player1[B]])
    journal.res[i] <- sum(y * x) / sqrt(phi * sum(x ^ 2))
}
names(journal.res) <- journal.abbr
```

Normal probability plot of journal residuals with 95% envelope (Figure 2) computed with function qqPlot from package car (Fox and Weisberg, 2011):

```
require(car)
qqPlot(journal.res, ylab = "Sorted journal residuals",
       xlab = "Normal quantiles")
```

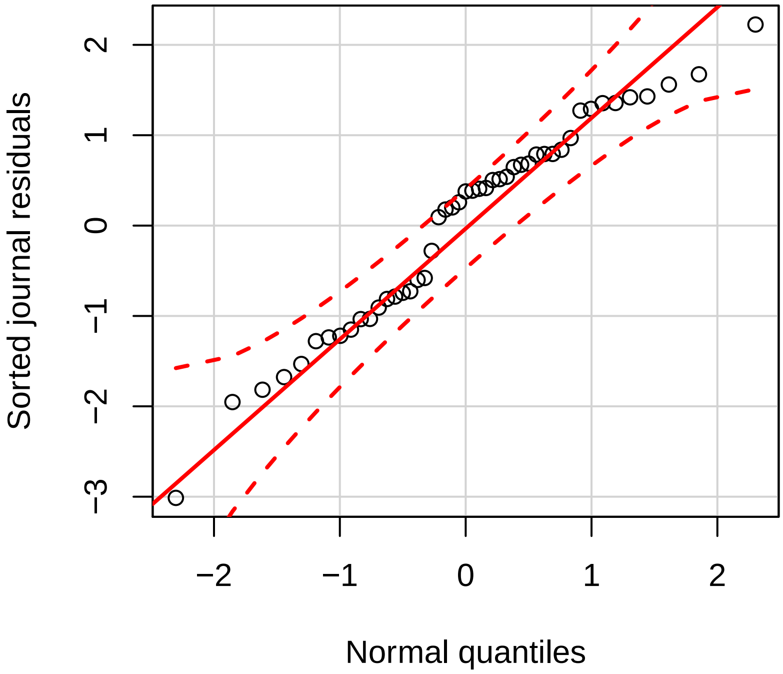

Figure 2: Normal probability plot of journal residuals with 95% envelope.

Scatterplot of journal residuals against estimated export scores (Figure 3 in this document):

```
plot(journal.res ~ coefs, ylab = "Journal residuals",
     xlab = "Export scores")
```

3.2 Quasi standard errors

Quasi standard errors discussed in Section 5.3 of the paper, computed with the `qvcalc` package (Firth, 2012):

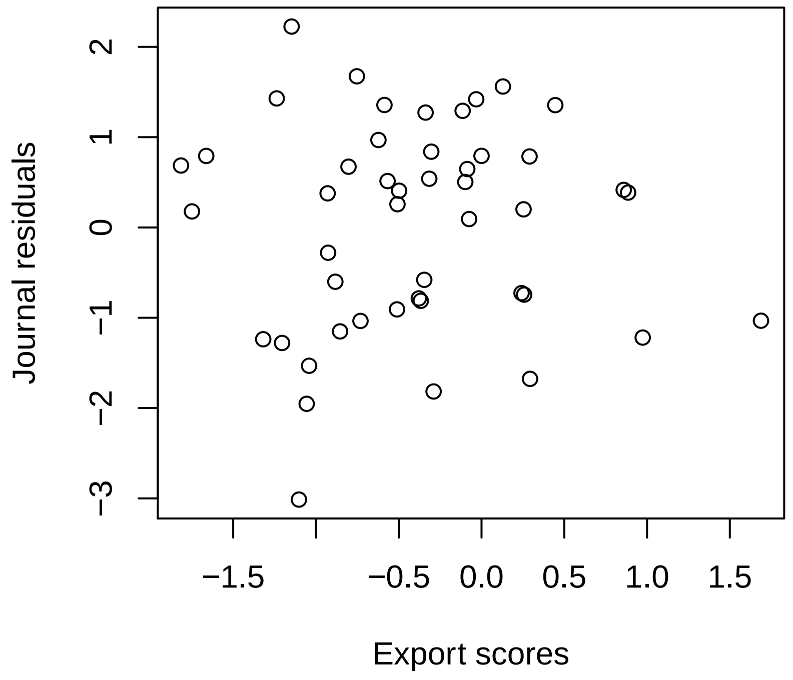

Figure 3: Scatterplot of journal residuals against estimated export scores.

```
require(qvcalc)
cov.matrix <- matrix(0, nrow = njournals, ncol = njournals)
cov.matrix[-1, -1] <- vcov(fit)
qse <- qvcalc(phi * cov.matrix , estimates = c(0, coef(fit)),
              labels = journal.abbr)
```

By default, the `BTm` function in the `BradleyTerry2` package fits the Bradley-Terry model with a 'corner constraint', *i.e.*, the export score of the first journal in alphabetic order is fixed to zero. In the paper, results are displayed with the 'more democratic' zero-sum parameterization:

```
export.scores <- qse$qvframe$estimate
export.scores <- export.scores - mean(export.scores)
names(export.scores) <- journal.abbr
```

Table of estimates and standard errors in decreasing order:

```
sort.id <- sort(export.scores, decreasing = TRUE,
                index.return = TRUE)$ix
fit.table <- data.frame(quasi = export.scores[sort.id],
                        qse = qse$qvframe$quasiSE[sort.id])
fit.table
```

```
##              quasi        qse
## JRSS-B  2.0911231 0.10513395
## AoS     1.3767352 0.07386382
## Bka     1.2884149 0.08119563
## JASA    1.2619488 0.06014319
## Bcs     0.8485257 0.07245316
## .               .          .
## .               .          .
## JAS    -1.4126066 0.15093299
```

Centipede plot (Figure 4) drawn with the `plotrix` package (Lemon, 2006):

```
require(plotrix)
segs <- apply(fit.table, 1, function(x) x[1] + c(0, -1.96, 1.96) * x[2])
centipede.plot(segs, left.labels = journal.abbr[sort.id],
               right.labels = round(export.scores[sort.id], 2),
               xlab = "Export Scores")
```

4 Ranking lasso

Read the ranking-lasso code (Masarotto and Varin, 2012):

```
source("R-code/ranking-lasso.R")
```

Computation of the complete path of the adaptive ranking lasso estimation[1]:

```
## time consuming
rlasso <- ranking.lasso(y = fit$model$Y, X = fit$model$X,
                        adaptive = TRUE)
```

The object `rlasso` returns a list containing the following components:

[1]**Warning**: The computation is relatively time-consuming, it takes about 70 seconds on a MacBook Air 1.8 GHz Intel Core i7 with 4 GB RAM. Function `ranking.lasso` is designed for moderate-size tournament data; the code can, and should, be re-designed for more efficient computation in larger applications.

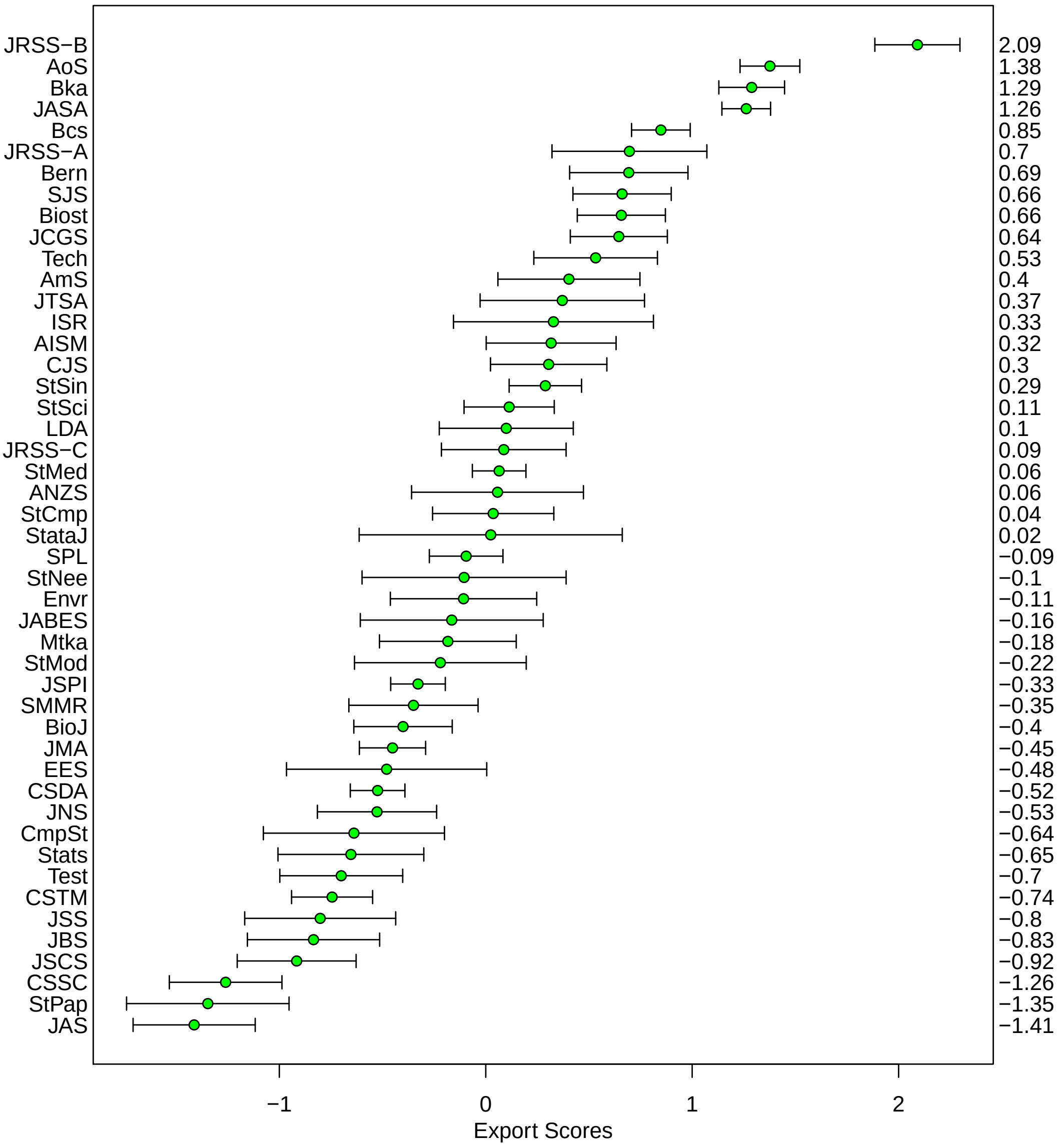

Figure 4: Centipede plot of estimated export scores with 95% comparison intervals.

`s` k-dimensional vector of standardized bounds $s/\max(s)$;

`beta` $k \times p$ matrix of ranking lasso estimates, where k is the number of bounds s and p is the number of model parameters;

`lik` k-dimensional vector of minus log-likelihoods computed at the various ranking lasso estimates;

`df` k-dimensional vector of the number of groups identified by the various ranking lasso estimates (degrees of freedom).

Zero-sum parameterization of lasso estimates:

```
lasso.scores <- cbind(0, rlasso$beta)
colnames(lasso.scores) <- journal.abbr
lasso.scores <- lasso.scores - rowMeans(lasso.scores)
```

Selection of best solution according to TIC defined in Section 5.5 of the paper:

```
tic <- 2 * rlasso$lik + 2 * phi * rlasso$df
best <- max(which.min(tic))
```

TIC identifies 11 groups, however the penultimate and the third to the last have grouped export scores that differ in the third decimal place only. Tables 4 and 5 of the paper are based upon results rounded to the second decimal, and thus the penultimate and the third-to-last groups are merged accordingly.
Update the summary fit table with the ranking lasso estimates:

```
fit.table <- data.frame(fit.table, lasso = lasso.scores[best, sort.id])
fit.table
```

```
##              quasi        qse      lasso
## JRSS-B   2.0911231 0.10513395  1.8696128
## AoS      1.3767352 0.07386382  1.1669128
## Bka      1.2884149 0.08119563  1.1061128
## JASA     1.2619488 0.06014319  1.1061128
## Bcs      0.8485257 0.07245316  0.6480128
## .                .          .          .
## .                .          .          .
## JAS     -1.4126066 0.15093299 -0.8826872
```

Ranking lasso path plot (Figure 5 in this document):

```
plot(x = c(0,rlasso$s,1), y = lasso.scores[, 1],
     ylim = range(lasso.scores), type = "l",
     xlab = "s/max(s)", ylab = "Export Scores")
for(i in 2:njournals)
        lines(x = c(0,rlasso$s,1), y = lasso.scores[,i] )
abline(v = rlasso$s[best], lty = "dashed")
abline(h = 0, lty = "dotted")
```

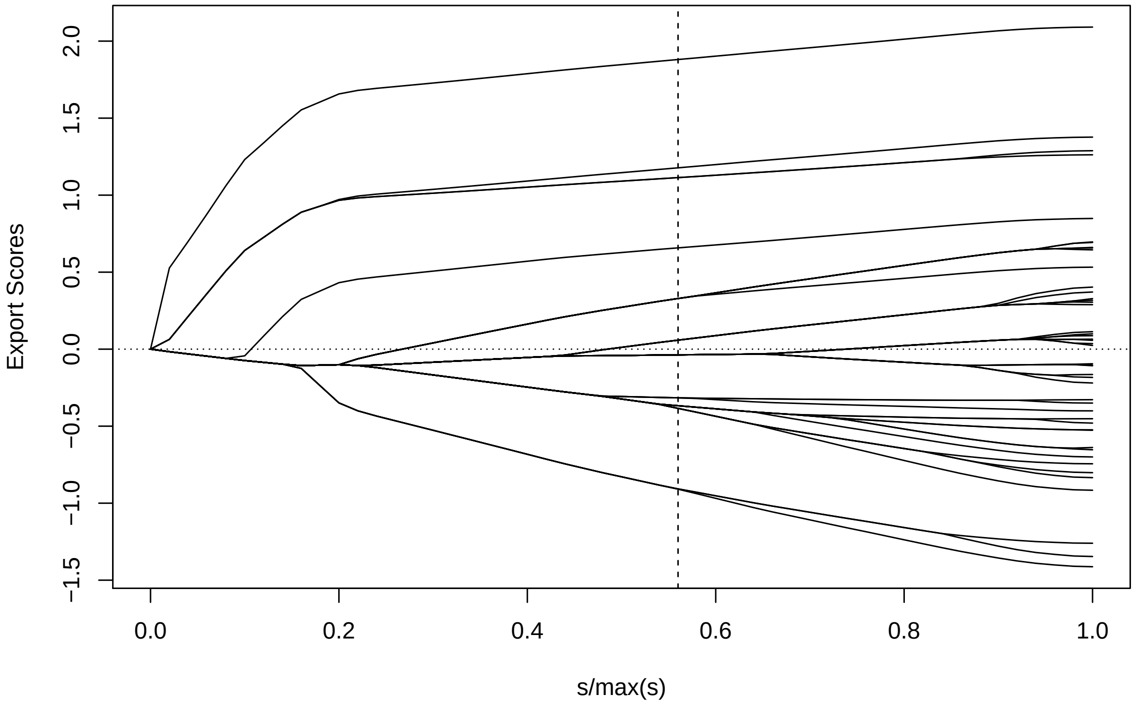

Figure 5: Path plot of the ranking lasso. The vertical dashed line corresponds to the best solution according to TIC.

5 Comparison with RAE 2008 results

5.1 Scoring the RAE submissions according to journal-ranking measures

The RAE 2008 submissions for Unit of Assessment 22 'Statistics and Operational Research' are online at
`http://www.rae.ac.uk/submissions/outstore/CSV-ANSI/ByUOA/22%20-%20Statistics%20and%20Operational%20Research.zip`
and from that source we use the two files `RA2.csv` and `Institution.csv`.

```
RA2 <- read.csv("Data/RAE-UoA22/RA2.csv", as.is = TRUE)
institutions <- read.csv("Data/RAE-UoA22/Institution.csv", as.is = TRUE)
```

The RA2 dataset contains details of all research outputs that were submitted for assessment.
Some minor data-tidying was needed, mainly to code coherently a joint submission that was made by Edinburgh and Heriot-Watt Universities, and to remove rows and columns that will not be used here:

```r
source("R-code/tidy-the-RAE-downloads.R")
```

The resulting data frame, named `RA2.ja`, contains only those RAE-submitted research outputs classified as ‘Journal Article’.
Now read in the file `RAE22-journals.csv` — the result of some rather tedious work! — which uniquely identifies each different representation of a journal name in the RA2 data. And use those unambiguous short names[2] in place of the text from the `Publisher` field of the RA2 data:

```r
journals <- read.csv("Data/RAE22-journals.csv", as.is = TRUE)
row.names(journals) <- journals$RAE.name
RA2.ja$Publisher <- journals[RA2.ja$Publisher, "shortName"]
```

Also a table of short names for the 30 departments of RAE sub-panel 22, ‘Statistics and Operational Research’, to use in the `Institution` field of the RA2 data:

```r
depts <- read.csv("Data/RAE22-depts.csv")
row.names(depts) <- as.character(depts$depts)
RA2.ja$Institution <- as.character(RA2.ja$Institution)
RA2.ja$Institution <- depts[RA2.ja$Institution, "shortName"]
```

Around 68% of the journal articles are in the JCR *Statistics and Probability* category. Let’s look at how that varies across the 30 departments:

```r
attach(RA2.ja)
tapply(Publisher, Institution, function(P) {1 - mean(P == "other")})

##          Bath      Bristol       Brunel    Cambridge       Durham
##     0.8750000    0.7000000    0.3666667    0.6610169    0.6111111
## Edinburgh+HW      Glasgow    Greenwich     Imperial         Kent
##     0.5607477    0.6428571    0.2500000    0.8400000    0.9069767
##     Lancaster        Leeds    Liverpool    LondonMet          LSE
##     0.7432432    0.7948718    0.7500000    0.5454545    0.7959184
##    Manchester    Newcastle   Nottingham           OU       Oxford
##     0.8666667    0.7073171    0.8787879    1.0000000    0.5888889
##      Plymouth         QMUL      Reading      Salford    Sheffield
##     0.6428571    0.8571429    0.6285714    0.2580645    0.5675676
##   Southampton    StAndrews  Strathclyde          UCL      Warwick
##     0.6857143    0.8636364    0.2045455    0.6250000    0.8115942

detach(RA2.ja)
```

[2]Note that the short names used here are different from the abbreviations defined in Table 1 of the paper.

Leave out Brunel, Greenwich, Salford and Strathclyde from the analysis, and eliminate their factor levels:

```
RA2.ja <- RA2.ja[!(RA2.ja$Institution %in%
                   c("Brunel", "Greenwich", "Salford", "Strathclyde")), ]
RA2.ja$Institution <- factor(as.character(RA2.ja$Institution))
attach(RA2.ja)
probstats.fraction.of.articles <- tapply(Publisher, Institution,
    function(P) {1 - mean(P == "other")})
detach(RA2.ja)
##  all of these remaining fractions are now > 0.5
probstats.fraction.of.articles

##         Bath      Bristol    Cambridge       Durham Edinburgh+HW
##    0.8750000    0.7000000    0.6610169    0.6111111    0.5607477
##      Glasgow     Imperial         Kent    Lancaster        Leeds
##    0.6428571    0.8400000    0.9069767    0.7432432    0.7948718
##    Liverpool    LondonMet          LSE   Manchester    Newcastle
##    0.7500000    0.5454545    0.7959184    0.8666667    0.7073171
##   Nottingham           OU       Oxford     Plymouth         QMUL
##    0.8787879    1.0000000    0.5888889    0.6428571    0.8571429
##      Reading    Sheffield  Southampton    StAndrews          UCL
##    0.6285714    0.5675676    0.6857143    0.8636364    0.6250000
##      Warwick
##    0.8115942
```

Now focus only on papers that appeared in the JCR *Statistics and Probability* journals. Around 72% of journal articles submitted by the remaining 26 departments are in that set:

```
RA2.ja.statprob <- RA2.ja[RA2.ja$Publisher != "other", ]
nrow(RA2.ja.statprob) / nrow(RA2.ja)

## [1] 0.7223587
```

The various journal-ranking scores — but only for those journals that appear in the RAE submissions — are collected in file `journal-scores.csv`:

```
journal.scores <- read.csv("Data/journal-scores.csv")
journal.scores$SM <- exp(journal.scores$SM)
journal.scores$SM.grouped <- exp(journal.scores$SM.grouped)
```

(The Stigler-model scores are exponentiated prior to the further analysis below.)
Next each journal article from the RA2 database is scored, as described in Section 6.2 of the paper:

```
row.names(journal.scores) <- journal.scores$shortName
RA2.ja.statprob$II <- journal.scores[RA2.ja.statprob$Publisher, "II"]
RA2.ja.statprob$I2 <- journal.scores[RA2.ja.statprob$Publisher, "I2"]
RA2.ja.statprob$I2no <- journal.scores[RA2.ja.statprob$Publisher, "I2no"]
RA2.ja.statprob$I5 <- journal.scores[RA2.ja.statprob$Publisher, "I5"]
RA2.ja.statprob$AI <- journal.scores[RA2.ja.statprob$Publisher, "AI"]
RA2.ja.statprob$SM <- journal.scores[RA2.ja.statprob$Publisher, "SM"]
RA2.ja.statprob$SM.grouped <- journal.scores[RA2.ja.statprob$Publisher,
                                             "SM.grouped"]
```

All of the 882 journal articles that remain here are scored by the 'global' measures `II`, `I2`, `I2no`, `I5` and `AI`, while around 65% of these articles are in the Statistics list from Table 1 of the paper and so are scored also by `SM` and `SM.grouped`. Let's look at how that fraction varies across the 26 departments:

```
attach(RA2.ja.statprob)
stats.fraction.of.probstats <- tapply(SM, Institution,
                                 function(x) {1 - mean(is.na(x))})
detach(RA2.ja.statprob)
stats.fraction.of.probstats

##          Bath      Bristol    Cambridge       Durham Edinburgh+HW
##     0.5476190    0.5714286    0.4358974    0.5454545    0.4166667
##       Glasgow     Imperial         Kent    Lancaster        Leeds
##     0.8888889    0.9523810    0.7692308    0.8545455    0.7096774
##     Liverpool    LondonMet          LSE   Manchester    Newcastle
##     0.2666667    0.8333333    0.4102564    0.3589744    0.7586207
##    Nottingham           OU       Oxford     Plymouth         QMUL
##     0.6551724    0.9615385    0.3773585    0.8888889    0.9666667
##       Reading    Sheffield  Southampton    StAndrews          UCL
##     0.7727273    0.5714286    0.9166667    0.8421053    0.8571429
##       Warwick
##     0.4821429
```

What fraction of articles are in the 47 Statistics journals, for each department?

```
stats.fraction.of.articles <- probstats.fraction.of.articles *
    stats.fraction.of.probstats
stats.fraction.of.articles

##          Bath      Bristol    Cambridge       Durham Edinburgh+HW
##     0.4791667    0.4000000    0.2881356    0.3333333    0.2336449
##       Glasgow     Imperial         Kent    Lancaster        Leeds
##     0.5714286    0.8000000    0.6976744    0.6351351    0.5641026
```

```
##     Liverpool     LondonMet           LSE    Manchester     Newcastle
##     0.2000000     0.4545455     0.3265306     0.3111111     0.5365854
##    Nottingham            OU        Oxford      Plymouth          QMUL
##     0.5757576     0.9615385     0.2222222     0.5714286     0.8285714
##       Reading     Sheffield   Southampton     StAndrews           UCL
##     0.4857143     0.3243243     0.6285714     0.7272727     0.5357143
##       Warwick
##     0.3913043
```

So thirteen of the 26 departments have less than half of their RAE-submitted journal articles in the identified 47 Statistics journals of Table 1 in the paper.

5.2 Journal-based mean scores for departments

Rate the departmental RAE submissions, by averaging over all journal articles scored:

```
attach(RA2.ja.statprob)
II.mean <- tapply(II, Institution, function(vec) mean(na.omit(vec)))
I2.mean <- tapply(I2, Institution, function(vec) mean(na.omit(vec)))
I2no.mean <- tapply(I2no, Institution, function(vec) mean(na.omit(vec)))
I5.mean <- tapply(I5, Institution, function(vec) mean(na.omit(vec)))
AI.mean <- tapply(AI, Institution, function(vec) mean(na.omit(vec)))
SM.mean <- tapply(SM, Institution, function(vec) mean(na.omit(vec)))
SM.grouped.mean <- tapply(SM.grouped, Institution,
                          function(vec) mean(na.omit(vec)))
detach(RA2.ja.statprob)
means <- data.frame(II.mean, I2.mean, I2no.mean, I5.mean, AI.mean,
                    SM.mean, SM.grouped.mean)
```

Do the same averaging but only using scores for the restricted set of 47 Statistics journals that were scored by the Stigler model:

```
RA2.ja.stat <- RA2.ja.statprob[!is.na(RA2.ja.statprob$SM), ]
attach(RA2.ja.stat)
II.mean.r <- tapply(II, Institution, function(vec) mean(na.omit(vec)))
I2.mean.r <- tapply(I2, Institution, function(vec) mean(na.omit(vec)))
I2no.mean.r <- tapply(I2no, Institution,
                      function(vec) mean(na.omit(vec)))
I5.mean.r <- tapply(I5, Institution, function(vec) mean(na.omit(vec)))
AI.mean.r <- tapply(AI, Institution, function(vec) mean(na.omit(vec)))
SM.mean.r <- tapply(SM, Institution, function(vec) mean(na.omit(vec)))
SM.grouped.mean.r <- tapply(SM.grouped, Institution,
                            function(vec) mean(na.omit(vec)))
```

```r
detach(RA2.ja.stat)
means.r <- data.frame(II.mean.r, I2.mean.r, I2no.mean.r,
              I5.mean.r, AI.mean.r, SM.mean.r, SM.grouped.mean.r)
```

Note that `SM.mean` and `SM.mean.r` are of course the same, as are `SM.grouped.mean` and `SM.grouped.mean.r`.

5.3 Comparison with the published RAE assessments

The file `RAE22-outputs-subprofiles.csv` is an extract, specific to the 26 departments of interest in RAE Unit of Assessment 22 'Statistics and Operational Research', from the full set of RAE-result 'sub-profiles' published online at `http://www.rae.ac.uk/pubs/2009/pro/#sub`. These sub-profiles are specific to the assessment of departments' *research outputs*:

```r
RAEprofiles <- read.csv("Data/RAE22-outputs-subprofiles.csv")
```

From that file can be constructed various candidate 'RAE score' values for the departments' research outputs:

```r
RAE.4star <- RAEprofiles$X4star
RAE.34star <- RAEprofiles$X4star + RAEprofiles$X3star
RAE.34star.wtd <- RAEprofiles$X4star + RAEprofiles$X3star/3
```

In what follows, as explained in the paper, we use `RAE.34star.wtd`.
We can now look at correlations between RAE score and the various journal-rating scores (as in Table 6 of the paper):

```r
cor(means, RAE.34star.wtd)

##                      [,1]
## II.mean         0.3409859
## I2.mean         0.4683247
## I2no.mean       0.4875652
## I5.mean         0.4978970
## AI.mean         0.7295643
## SM.mean         0.8140549
## SM.grouped.mean 0.8188923
```

The second row of Table 6 shows correlations based on scoring only the smaller subset of 47 Statistics journals:

```r
cor(means.r, RAE.34star.wtd)

##                        [,1]
## II.mean.r         0.3417413
## I2.mean.r         0.6878651
## I2no.mean.r       0.7030977
## I5.mean.r         0.7340262
## AI.mean.r         0.7919254
## SM.mean.r         0.8140549
## SM.grouped.mean.r 0.8188923
```

The graphs shown in Figure 6 of the paper are drawn as follows:

```r
## Left panel of Figure 6
the.line <- lm(RAE.34star.wtd ~ SM.grouped.mean,
               weights = as.numeric(stats.fraction.of.articles > 0.5))
plot(SM.grouped.mean, RAE.34star.wtd,
     xlab = "Mean of (grouped) Stigler-Model export scores",
     ylab = "RAE score (4* and 3* percentages, weighted 3:1)",
     main = "RAE 2008 results vs Stigler Model mean score")
abline(the.line, lty = "dashed")
```

The outlier-identifying labels seen in Figure 6 of the paper were added by hand, using the `identify` function.

```r
## Right panel of Figure 6
plotting.colours <- ifelse(stats.fraction.of.articles > 0.5,
                           "black", "white")
plot(SM.grouped.mean, RAE.34star.wtd,
     xlab = "Mean of (grouped) Stigler-Model export scores",
     ylab = "RAE score (4* and 3* percentages, weighted 3:1)",
     main = "RAE 2008 vs Stigler Model: Restricted to
the 13 most 'Statistical' departments",
     col = plotting.colours)
abline(the.line)
```

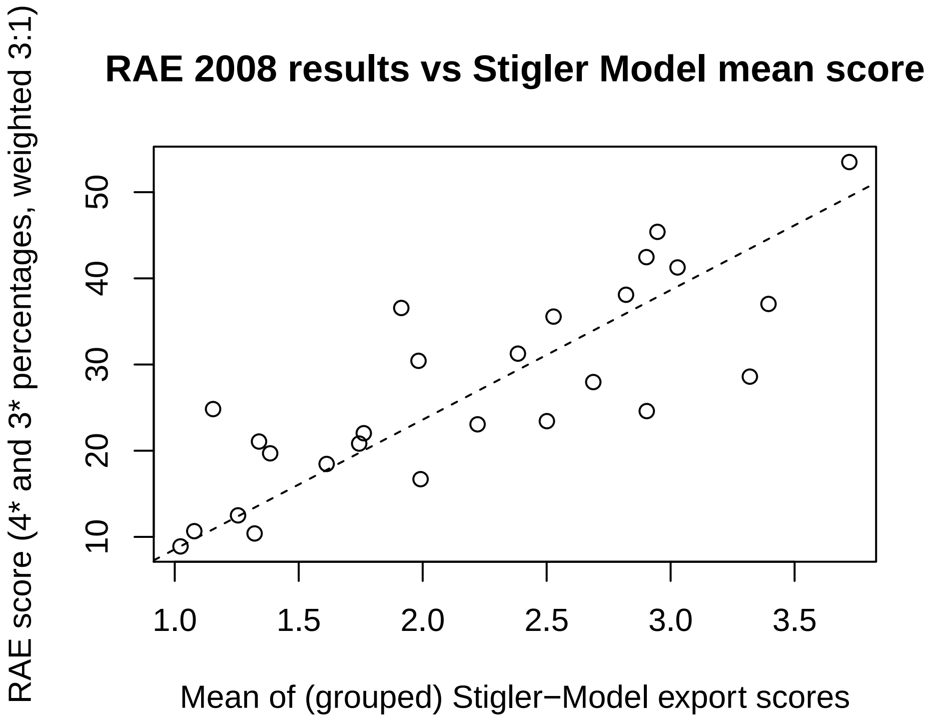
RAE 2008 results vs Stigler Model mean score
RAE score (4* and 3* percentages, weighted 3:1)
10
20
30
40
50
1.0
1.5
2.0
2.5
3.0
3.5
Mean of (grouped) Stigler−Model export scores

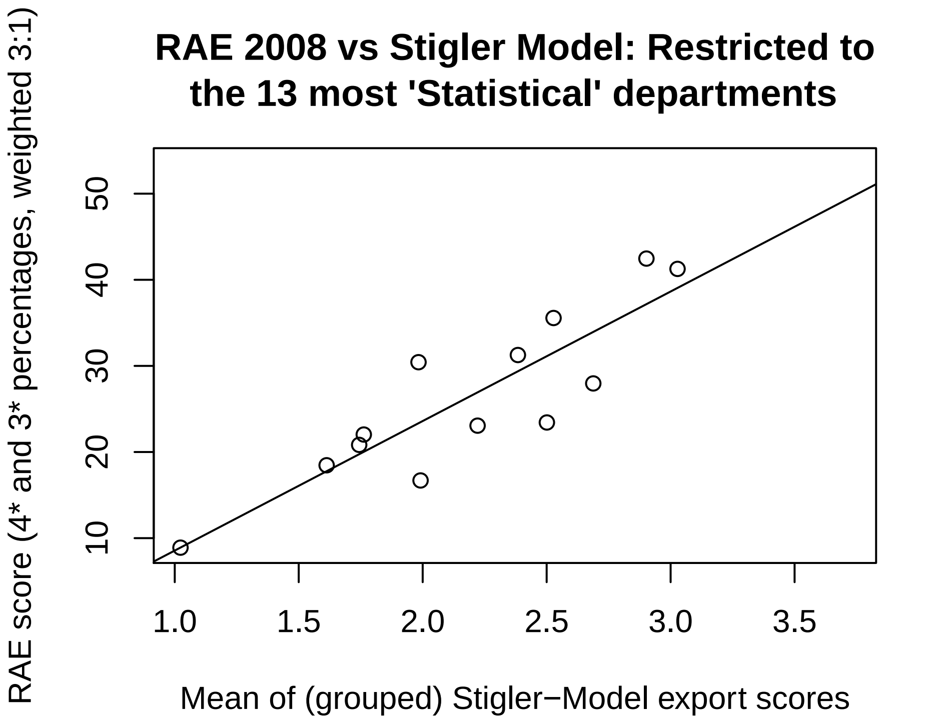
RAE 2008 vs Stigler Model: Restricted to the 13 most 'Statistical' departments
RAE score (4* and 3* percentages, weighted 3:1)
10
20
30
40
50
1.0
1.5
2.0
2.5
3.0
3.5
Mean of (grouped) Stigler−Model export scores

References

Firth, D. (2012). qvcalc: Quasi variances for factor effects in statistical models. R package version 0.8-8. URL http://CRAN.R-project.org/package=qvcalc

Fox, J. and Weisberg, S. (2011). *An R Companion to Applied Regression*, Second Edition. Thousand Oaks CA: Sage. URL http://socserv.socsci.mcmaster.ca/jfox/Books/Companion

Lemon, J. (2006) Plotrix: A package in the red light district of R. *R-News* **6** (4), 8–12.

Masarotto, G. and Varin, C. (2012). The ranking lasso and its application to sport tournaments. *Annals of Applied Statistics* **6**, 1949–1970.

R Core Team (2015). *R: A language and environment for statistical computing.* R Foundation for Statistical Computing, Vienna, Austria. URL http://www.R-project.org/

Turner, H. and Firth, D. (2012). Bradley-Terry models in R: The BradleyTerry2 package. *Journal of Statistical Software*, **48** (9), 1–21. URL http://www.jstatsoft.org/v48/i09/

Varin, C., Cattelan, M. and Firth, D. (2015). Statistical modelling of citation exchange between statistics journals. *Journal of the Royal Statistical Society Series A*, to appear.
